# New fluoroethyl phenylalanine analogues as potential LAT1-targeting PET tracers for glioblastoma

**DOI:** 10.1038/s41598-019-40013-x

**Published:** 2019-02-27

**Authors:** Jeroen Verhoeven, Fabian Hulpia, Ken Kersemans, Julie Bolcaen, Stef De Lombaerde, Jan Goeman, Benedicte Descamps, Giorgio Hallaert, Caroline Van den Broecke, Karel Deblaere, Christian Vanhove, Johan Van der Eycken, Serge Van Calenbergh, Ingeborg Goethals, Filip De Vos

**Affiliations:** 1 Laboratory of Radiopharmacy, Ghent University, Ghent, Belgium; 2 Laboratory for Medicinal Chemistry, Ghent University, Ghent, Belgium; 3 Ghent University Hospital, Department of Nuclear Medicine, Ghent, Belgium; 4 Laboratory for Organic and Bio-organic synthesis, Ghent University, Ghent, Belgium; 5 IBiTech-MEDISIP Ghent University, Department of Electronics and Information Systems, Ghent, Belgium; 6 Ghent University Hospital, Department of Neurosurgery, Ghent, Belgium; 7 Ghent University Hospital, Department of Pathology, Ghent, Belgium; 8 Ghent University Hospital, Department of Radiology and Medical Imaging, Ghent, Belgium

## Abstract

The use of *O*-(2-[^18^F]fluoroethyl)-l-tyrosine ([^18^F]FET) as a positron emission tomography (PET) tracer for brain tumor imaging might have some limitations because of the relatively low affinity for the L-type amino acid transporter 1 (LAT1). To assess the stereospecificity and evaluate the influence of aromatic ring modification of phenylalanine LAT1 targeting tracers, six different fluoroalkylated phenylalanine analogues were synthesized. After *in vitro* K_i_ determination, the most promising compound, 2-[^18^F]-2-fluoroethyl-l-phenylalanine (2-[^18^F]FELP), was selected for further evaluation and *in* vitro comparison with [^18^F]FET. Subsequently, 2-[^18^F]FELP was assessed *in vivo* and compared with [^18^F]FET and [^18^F]FDG in a F98 glioblastoma rat model. 2-[^18^F]FELP showed improved *in vitro* characteristics over [^18^F]FET, especially when the affinity and specificity for system L is concerned. Based on our results, 2-[^18^F]FELP is a promising new PET tracer for brain tumor imaging.

## Introduction

**Figure 1:**
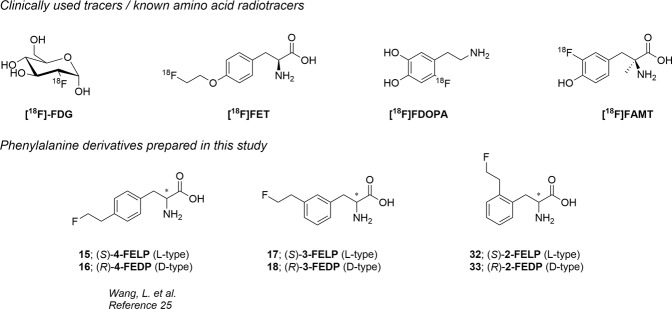
Structures of clinically used radiotracers or previously described amino acid tracers (upper part). Structures of the prepared modified phenylalanine analogs in this manuscript (lower part).

In neuro-oncology, the potential of conventional magnetic resonance imaging (MRI) to differentiate neoplastic tissue from non-specific changes induced by treatment may be limited after therapeutic interventions (e.g. radiation therapy, neurosurgical resection, and chemotherapy). The molecular imaging modality positron emission tomography (PET) provides additional information on tumor metabolism, which allows for more accurate diagnostics and therapy response assessment^1–3^. During the last decades, a variety of molecular targets have been assessed for brain tumor imaging by specific PET tracers. Especially amino acid (AA) based tracers received a lot of attention^2,4^. In contrast to [^18^F]fluoro-2-deoxy-d-glucose ([^18^F]FDG, Fig. 1), the uptake of radiolabeled AAs is low in normal gray matter of the brain which results in higher tumor-to-background ratios^5^. A considerable advantage of AA over nucleoside- or choline-based tracers is the ability to pass through the intact blood-brain barrier (BBB) due to the presence of AA transporters. The transport of AA into cells is mediated by specific membrane associated carrier proteins, which are classified into different transporter systems based on criteria such as sodium dependence, substrate specificity, kinetics, etc.^6^. Amongst the currently known AA transporters, system L and system ASC are overexpressed in most tumor tissues, rendering these convenient targets for metabolic imaging of tumor cells^7–9^. The most widespread ^18^F labeled tracers that have been developed are *O*-(2-[^18^F]fluoroethyl)-l-tyrosine ([^18^F]FET) and 3, 4-dihydroxy-6-[^18^F]-fluoro-l-phenylalanine ([^18^F]FDOPA; Fig. 1). Unfortunately, both are not specific for the L-type amino acid transporter 1 (LAT1) and the latter shows uptake in the striatum due to participation in the dopamine synthesis pathway^10–13^. Most of the amino acid PET tracers, including [^18^F]FET, still suffer from various levels of physiological background uptake and uptake in non-neoplastic lesions such as acute inflammatory lesions as a result from multiple sclerosis or brain abscesses^14–17^. An advantage of these non-natural amino acid tracers is the insusceptibility to *in vivo* metabolism resulting in the absence of radiolabeled metabolites^18^. Different PET and single-photon emission computed tomography (SPECT) tracers based on tyrosine, tryptophan and glutamine have been developed for LAT1 or ASCT2 targeting^19–22^, of which the most promising results have been reported for 3-fluoro-l-α-methyltyrosine ([^18^F]FAMT, Fig. 1). However, the existing synthetic methods for the preparation of [^18^F]FAMT suffer from low chemical yields, which limits the availability of this tracer for clinical use^23,24^. Based on the structure of [^18^F]FET, Wang *et al*. have developed *para*-fluoroethyl (**15** & **16**, Fig. 1) and -fluoropropyl alkylated phenylalanine derivatives, where the former showed appreciable uptake in the 9 L glioma tumor model^25^.

In this study we want to develop a practical synthesis for different phenylalanine derivatives and compare their affinity for the LAT1 transporter. As there are conflicting reports on the preferred stereochemistry of the phenylalanine/tyrosine analogues for the LAT1 transporter^26–28^, both enantiomers (*S*) and (*R*) of *ortho*- (**32** & **33**) and *meta*- (**17** & **18**) substituted 2-fluoroethyl phenylalanine derivatives (Fig. 1) were synthesized and their K_i_ was determined in LAT1 overexpressing F98 glioblastoma (GB) cells^29^. Previously reported *para*-substituted derivatives (**15** & **16**) were included to allow comparison with the novel analogs in our *in vitro* system. The *in vitro* uptake of the most promising compound was compared to [^18^F]FET and its uptake in a rat F98 GB model was evaluated.

## Results

### Chemistry

**Figure 2:**
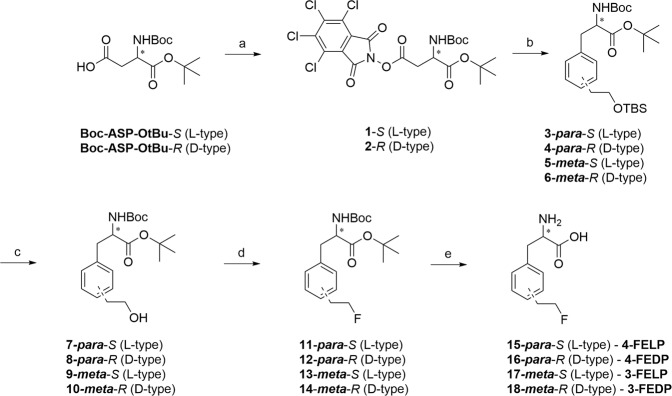
Reagents and conditions: a) TCNHPI, DIC, DCM, rt, 59% (**1**), 70% (**2**); b) Boronic acid (**21** or **22**), NiCl_2_.6H_2_O, BBBPY, Et_3_N, DMF/1, 4-dioxane (1/10), 75 °C, overnight, 46% (**3** & **4**), 59% (**5**), 34% (**6**); c) 1 M TBAF in THF/THF, rt, 83% (**7**), 77% (**8**), 71% (**9**), 73% (**10**); d) DAST, DCM, 0 °C–rt, 42% (**11**), 37% (**12**), 50% (**13**), 29% (**14**); e) TFA/DCM (1/1), rt, 39% (**4-FELP**, **15**), 35% (**4-FEDP**, **16**), 46% (**3-FELP**, **17**), 46% (**3-FEDP**, **18**).

Initially, we set out to employ a common starting material (**1** & **2**, Fig. 2) to gain access to all the different (*ortho*-, *meta*- and *para*-) substituted phenylalanine amino acids. This approach was inspired by recent work of the Baran group, which described arylation of activated glutamate esters with boronic acids under Ni-catalysis^30^. This route could enable access to all different phenylalanine derivatives from a single amino acid precursor.

**Figure 3:**
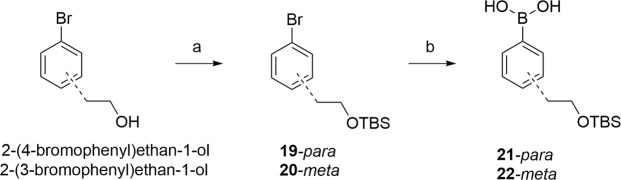
Reagents and conditions: a) TBSCl, imidazole, DMF, 98% (**19**), quant. (**20**); b) i. n-BuLi (1.6 M in hexanes), THF, −78 °C; ii. B(OMe)_3_, −78 °C–rt; iii. aq. HCl, 42% (**21**), 55% (**22**).

The synthesis started from suitably protected commercially available l- or d-aspartic acid derivatives (Fig. 2). After activation of the side chain carboxylic acid as a N-hydroxytetrachlorophtalimide ester, **1** or **2** was reacted with the corresponding boronic acids (**21** (*para*) or **22** (*meta*)) to give the substituted phenylalanine derivatives **3**–**6** in acceptable yields. The preparation of boronic acid derivatives is depicted in Fig. 3. Commercially available bromo-phenylethanol was protected with TBSCl and subsequently transformed into the corresponding boronic acid by Li/Br exchange and trapping with trimethylborate^31,32^.

With the boronic acids in hand, cross-coupling was found successful for the synthesis of both *para*- and *meta*- substituted derivatives (Fig. 2). Next, the primary alcohol was unmasked by TBAF in THF, and fluorinated with DAST and deprotected to give rise to the fluorinated amino acids **15**–**18**.

**Figure 4:**
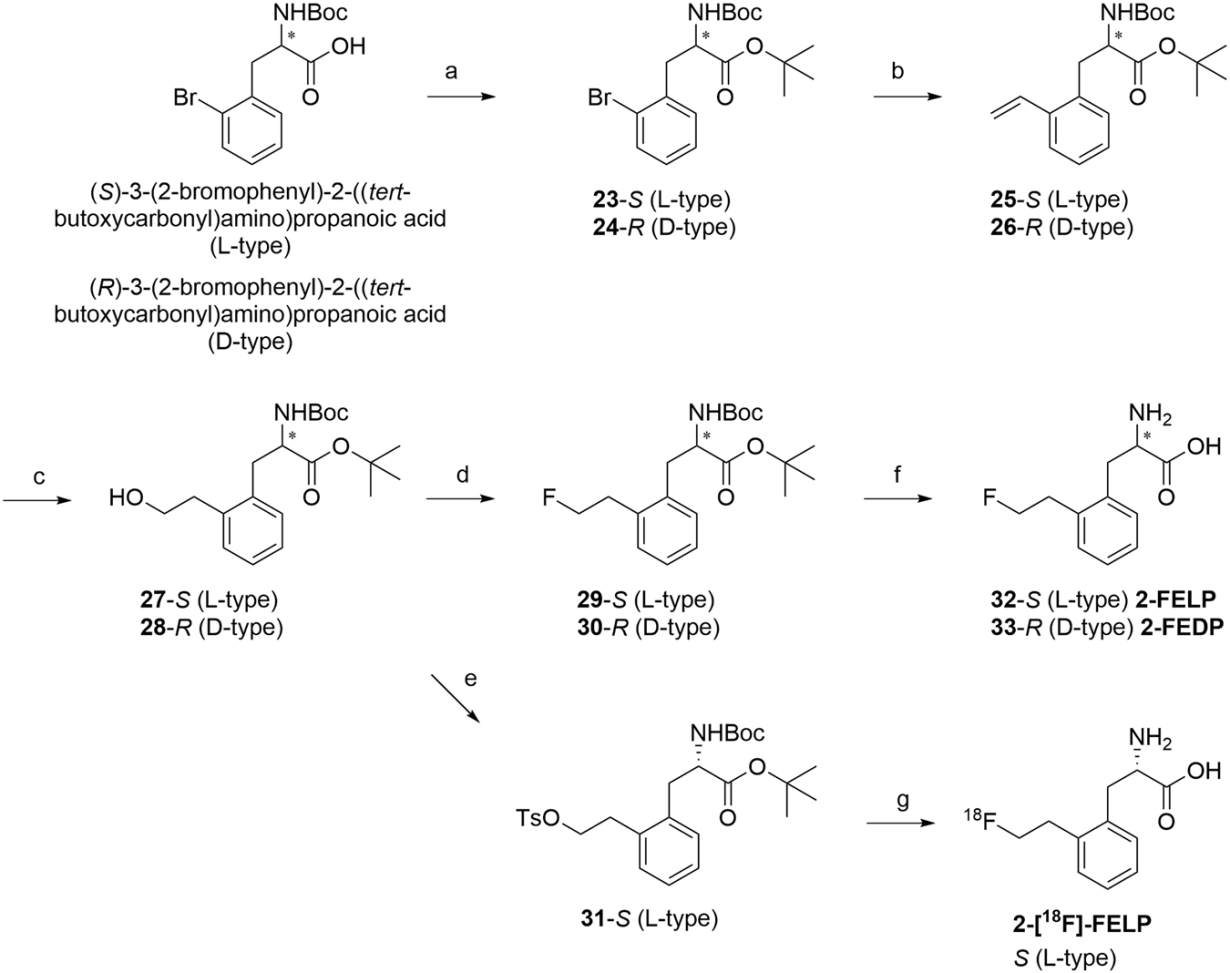
Reagents and conditions: a) TBTA, DCM, overnight, 90% (**23**), 72% (**24**); b) vinyl-Sn(nBu)_3_, LiCl, Pd(Ph_3_P)_2_Cl_2_, DMF, 70 °C, overnight, 51% (**25**), 67% (**26**); c) i. BH_3_^.^THF, THF, 0 °C–rt; ii. NaBO_3_.H_2_O, water, rt, 66% (**27**), 61% (**28**); d) DAST, DCM, 0 °C–rt, 45% (**29**), 54% (**30**); e) Tosylchloride, DMAP, Et_3_N, DCM, rt, 56% (**31**); f) TFA/DCM (1/1), rt, 23% (**2-FELP, 32**), 49% (**2-FEDP**, **33**); g) i. [^18^F]F^−^/Kryptofix®222/K^+^ complex, MeCN, 100 °C, 15 min, ii. 2 M aq. HCl, 15 min, iii. 4 M aq. NaOH, iv. HPLC purification.

Attempts were made to prepare the *ortho*-substituted derivatives (**32** & **33**, Fig. 4) with the same strategy, however, this was met with several issues. Firstly, the preparation of TBS-protected *ortho*-boronic acid was troublesome, as the major product observed was the corresponding oxaborole (data not shown). This could be circumvented by employing the corresponding TIPS-protected alcohol. Next, the cross-coupling reaction with the *ortho*-substituted boronic acid failed to give appreciable amount of the coupled product, probably due to steric hindrance. This was confirmed by attempting the same reaction with commercially available 2-ethylphenylboronic acid and activated amino acid ester **1** under the same reaction conditions without success. Then, attempts with altered reaction conditions (temperature, catalyst loading and/or catalytic system (bathophenanthroline^30^ instead of 4, 4′-di-tert-butyl-2, 2′-dipyridyl, BBBPY)) also did not deliver the desired product (data not shown). Therefore, it was decided to switch to another synthetic route, employing a pre-functionalized phenylalanine starting material (Fig. 4).

This synthetic approach was based on the literature route described for the synthesis of *para*-substituted phenylalanine amino acids^25,28^. Commercially available N-Boc-2-bromophenylalanine was protected as its *tert*-butyl ester with t-butyl 2, 2, 2-trichloroacetimidate (TBTA) and vinylated with a Stille coupling reaction to give **25** and **26**. Next, hydroboration introduced the primary alcohol, which was either fluorinated and deprotected to give the free amino acids 2-FELP (**32**) and 2-FEDP (**33**), or tosylated with tosylchloride to give **31** to serve as radiolabelling precursor.

### *In vitro* experiments

#### Flow cytometry

The expression of LAT1 in the F98 cells was confirmed by flow cytometry (Supplementary Fig. 4).

#### Concentration dependency using [2, 3, 4, 5, 6-^3^H]-l-phenylalanine

**Figure 5:**
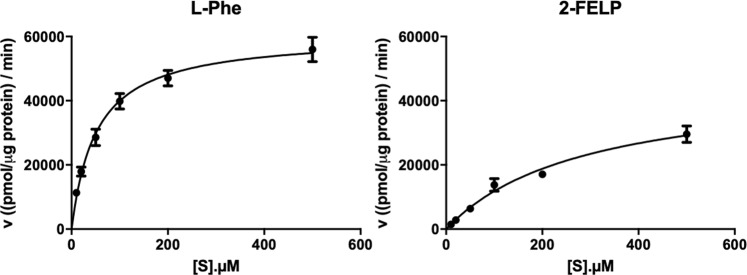
Left: Concentration dependence uptake of [^3^H]-l-Phe. Right: Concentration dependence of [^3^H]-l-Phe uptake in presence of **3-FELP**. A 1-min incubation time was used. Michaelis-Menten plots were obtained to calculate K_m_ (left) and K_m,app_ (right).

The affinity constant K_i_ of all compounds for the system L transporters was determined to identify the optimal derivatization position on the phenyl ring, as well as the preferred stereochemistry for system L targeting. The K_i_-values are shown in Table 1 and representable K_m_ and K_m,app_ charts are shown in Fig. 5 (see also Supplementary Information). Between all compounds a statistically significant difference was found (*p* = 0.002), except between **3FEDP**–**3FELP** and **3FEDP**–**4FELP** (*p* = 0.065 and 0.485 respectively).

**Table 1:** Ki values (expressed in µM, mean ± SD, *n* = 6) of the *ortho*-, *meta*- and *para*- substituted compounds, FET and l-phenylalanine.

Compound	K_i_ value (µM)	Compound	K_i_ value (µM)
	16.09 ± 3.74		359.40 ± 47.10
	48.95 ± 4.92		83.75 ± 28.10
	85.55 ± 4.40		>2000
	307.20 ± 4.29		51.53 ± 7.80

### Radiochemistry

[^18^F]FET and 2-[^18^F]FELP were prepared as described below. Enantiomerically pure (ee > 99%) 2-[^18^F]FELP was synthesized in 90 min with high radiochemical purity (>95% as measured by analytical HPLC and TLC), good yield (10–30% with respect to the starting activity at the end of F18 production, not corrected for decay), and high molar activity (243.7 ± 11.9 GBq/µmol). Chromatograms can be found in the Supplementary Information.

2-[^18^F]FELP exhibits a logP_oct_ value of −1.16 ± 0.011 which is in accordance with the logP_oct_ value of phenylalanine (logP_oct_ = −1.38)^33^ while a logP_oct_ value of −1.48 ± 0.047 was observed for [^18^F]FET.

#### Concentration and time dependency using 2-[^18^F]FELP or [^18^F]FET

**Figure 6:**
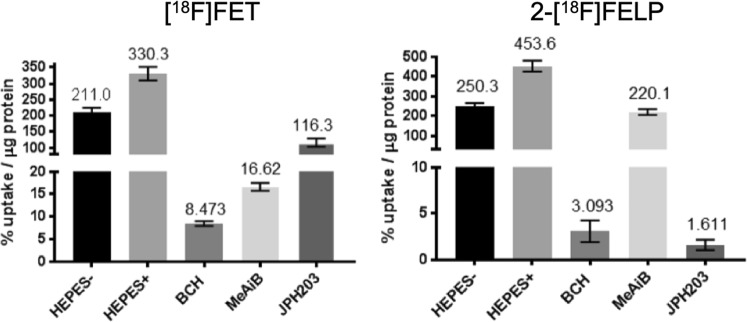
Maximum uptake of [^18^F]FET and 2-[^18^F]FELP in presence of sodium free (HEPES-) or sodium rich buffer (HEPES+) and supplemented with transporter specific inhibitors. Inhibitors: BCH for system L and b^0,+^, MeAiB for system A, JPH203 for LAT1 (expressed as % uptake/µg protein, mean ± SD, *n* = 6).

Since the lowest K_i_-value was obtained for **2-FELP**, the uptake characteristics of this tracer were investigated further and compared with the uptake characteristics of **FET** (Fig. 6). The maximum uptake of [^18^F]FET, used as a reference, and 2-[^18^F]FELP was studied in F98 cells. The latter compound seemed more sodium dependent than [^18^F]FET (44% vs 36% more uptake in presence of Na^+^). BCH, a system L and b^0,+^ inhibitor, blocked the 2-[^18^F]FELP or [^18^F]FET uptake by >99% and 97%, respectively. In the presence of the system A inhibitor, MeAiB, 2-[^18^F]FELP was inhibited less (51.5% less uptake) than that of [^18^F]FET (95% less uptake). The most interesting difference was observed by studying the effect of the recently developed LAT1 specific inhibitor JPH203, which inhibited the uptake of [^18^F]FET by 65% and that of 2-[^18^F]FELP by >99%. Statistical analysis showed a significant difference between the uptake in HEPES+ buffer and HEPES-/BCH/MeAiB/JPH203 (*p* = 0.002) and between BCH and MeAiB (*p* = 0.002) for both tracers. However, no difference could be found when the BCH and JPH203 uptake of 2-[^18^F]FELP (*p* = 0.065) was compared to [^18^F]FET (*p* = 0.002). These data indicate that LAT1 is more involved in the 2-[^18^F]FELP than in the [^18^F]FET uptake. The uptake in V_0_ conditions of both 2-[^18^F]FELP and [^18^F]FET as a function of the concentration of non-radioactive analogue (0.01–0.5 mM) was saturable following the Michaelis-Menten equation. The related Michaelis-Menten plot resulted in a K_m_ value (expressed in µM, mean ± SD, *n* = 6) of 12.54 ± 3.82 µM for 2-[^18^F]FELP and 312.9 ± 25.6 µM for [^18^F]FET. Moreover, the K_m_ values were not significantly different from their corresponding K_i_ values determined earlier (2-[^18^F]FELP: *p* = 0.165; [^18^F]FET: *p* = 0.558).

### *In vivo* experiments

**Figure 7:**
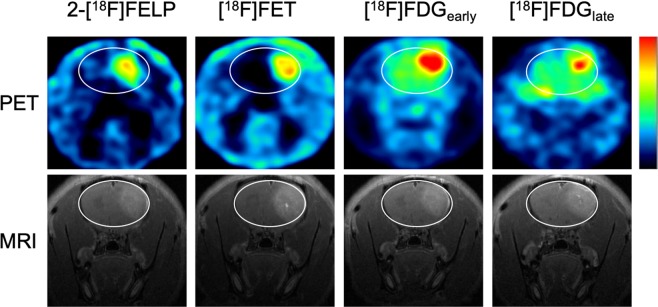
µPET images (upper row) and contrast-enhanced T1-weighted MRI (bottom row) of GB in rats. A high heterogeneous tumor uptake was visible on 2-[^18^F]FELP (A) and [^18^F]FET PET (B) (100–120 min post-injection) with a relative low uptake in healthy brain tissue. Both [^18^F]FDG PET scans, 60 min (C) and 240 min post-injection (D), showed a homogeneous intense uptake in the tumor, however, a higher uptake the in surrounding normal brain tissue can be noted. The latter is clearly lower on the delayed [^18^F]-FDG PET scan (D).

**Figure 8:**
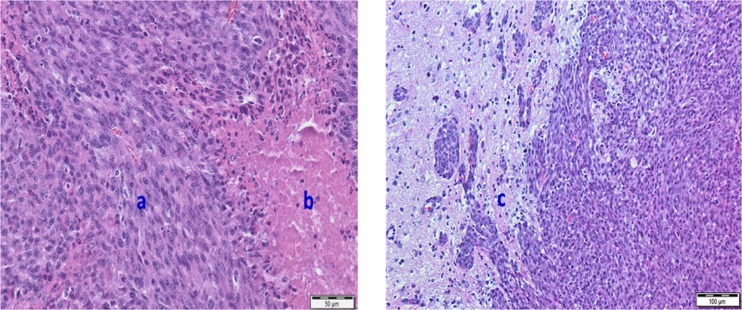
Hematoxylin and eosin stained paraffin sections of the rat brain confirming the presence of GB characteristics: High cellularity and nuclear pleiomorphism (a), microvascular proliferation and tumor necrosis (b), visible tumor infiltration with vessel recruitment at rim of tumor (c).

Two weeks after inoculation of F98 cells, tumors were visible in the right frontal region of rats on T2-weighted and contrast-enhanced T1-weighted MRI (Fig. 7). Hematoxylin and eosin stained paraffin sections revealed tumor necrosis, microvascular proliferation, nuclear atypia and increased mitosis, confirming the presence of GB (Fig. 8)^11,34^.

### Semi-quantitative analysis

**Figure 9:**
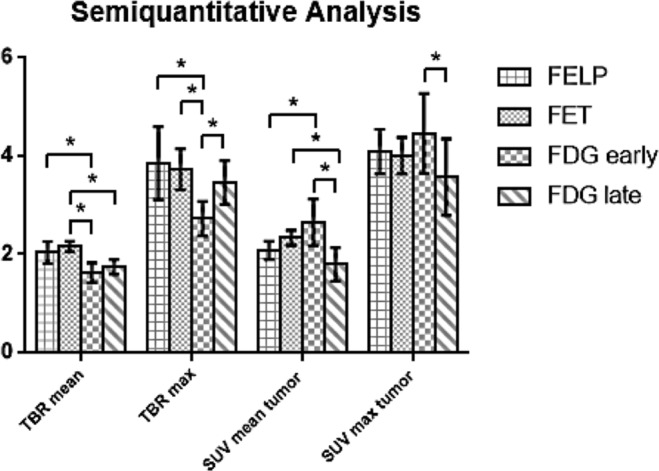
Comparison of the TBR_mean_, TBR_max_, SUV_mean_ and SUV_max_ in F98 GB tumor between 2-[^18^F]FELP, [^18^F]FET and [^18^F]FDG PET. *p value < 0.05.

**Figure 10:**
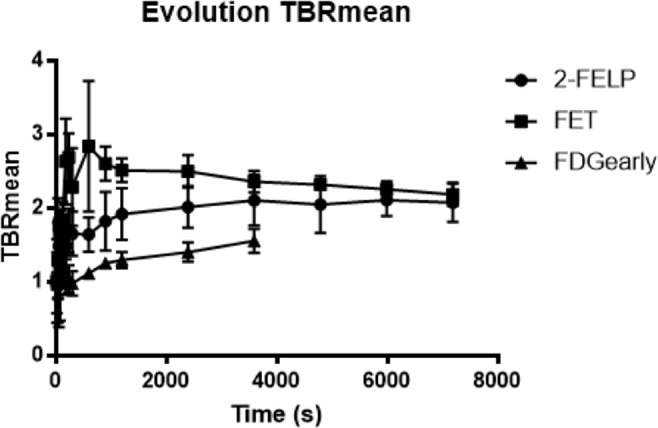
The TBR_mean_ values of the dynamic 2-[^18^F]FELP and [^18^F]FET PET scans were significantly higher in comparison with [^18^F]FDG. No significant differences were found between the AA PET scans.

The SUV_mean_, SUV_max_, TBR_mean_ and TBR_max_ were calculated for the different radiotracers (Fig. 9). No significantly different TBR and SUV values were found between 2-[^18^F]FELP and [^18^F]FET PET (TBR_mean_: *p* = 0.386; TBR_max_: p = 0.773; SUV_mean_: *p* = 0.0830; SUV_max_: *p* = 1.00). Significant differences were observed between 2-[^18^F]FELP and [^18^F]-FDG_early_ (TBR_mean_: *p* = 0.043; TBR_max_: *p = *0.021; SUV_mean_: *p* = 0.021), [^18^F]FET and [^18^F]FDG_early_ (TBR_mean_: *p* = 0.021; TBR_max_: *p* = 0.021), [^18^F]FET and [^18^F]FDG_late_ (TBR_mean_: *p* = 0.021; SUV_mean_: *p* = 0.043), [^18^F]FDG_early_ and [^18^F]FDG_late_ (TBR_max_: *p* = 0.043; SUV_mean_: *p* = 0.043). The TBR_mean_ values of the dynamic scans are shown in Fig. 10. The TBR_mean_ values of [^18^F]FDG PET scans were significantly lower in comparison with the 2-[^18^F]FELP and [^18^F]FET (*p* = 0.043 and 0.021 respectively). No significant differences were found between the AA PET scans (*p* = 0.386).

## Discussion

The L-type AA transporter 1 transports aromatic or branched AA that are essential for cellular proliferation and growth. Due to its low expression and distribution in non-pathological cells and upregulation in glioma cells, this AA transporter received a lot of attention as potential target for tumor imaging^1,13^. Molecular imaging using PET may provide relevant additional information on tumor metabolism and may be useful in case of ambiguous MRI findings following neuro-oncological treatment^35^. In this study, two recently developed fluoroalkyl phenylalanine analogues, **4-FELP** and **4-FEDP**^25^, together with four new compounds, **2-FELP**, **2-FEDP**, **3-FELP**, **3-FEDP** were successfully synthesized and compared *in vitro* to the reference compound FET, currently used in the clinic. The influence of the stereochemistry on the affinity for the LAT1 transporter was evaluated as there are some conflicting reports in literature. When comparing ^123/125^I-labelled 2-iodo-l-phenylalanine and 2-iodo-d-phenylalanine in R1M cells^26^, Kersemans *et al*. observed a high affinity of the LAT transporter for the d-enantiomer, while Bauwens *et al*.^27^ reported a higher K_i_ values for d-tyrosine than for 2-I-l-tyrosine. In this study, the *in vitro* affinity of the d-enantiomers for the LAT1 transporter in F98 GB cells was lower than the affinity of the l-enantiomers, except for the *meta*-substituted enantiomers. This promiscuity regarding the (stereochemical) substrate preference was observed in a recent publication as well^36^. When considering the K_i_ values of the l-enantiomers, the preference of the position of the 2-fluoroethyl substituent on the aromatic ring of phenylalanine was as follows: *ortho* > *meta* > *para*.

Based on earlier studies^36,37^, it has been shown that *meta*-substitution appears to be the most tolerant towards substitution. Therefore, it is quite remarkable that the *ortho*-substituted 2-FELP displays the highest *in vitro* affinity.

The radiofluorinated 2-[^18^F]FELP and [^18^F]FET were successfully prepared on a Synthra RN plus module using identical reaction and purification conditions. The relatively straightforward synthesis method, as described earlier, simplifies the availability for clinical use, which has been an issue for [^18^F]FAMT^38^.

In the present study, the *in vitro* influx of 2-[^18^F]FELP and [^18^F]FET was characterized in F98 GB cells by means of inhibition experiments, involving the recently identified selective LAT1 inhibitor JPH203, which has structural analogy to tyrosine. Moreover, this compound effectively inhibits the proliferation of various tumor cells injected into nude mice^39^. The uptake of 2-[^18^F]FELP was considerably lower in presence of the inhibitor JPH203 compared to the uptake of [^18^F]FET. This indicates that 2-[^18^F]FELP is predominantly transported by the LAT1 transporter. The results for [^18^F]FET are in conformity with the study conducted by Langen *et al*.^40^ and the limited inhibition by JPH203 could be explained by LAT2 non-specific uptake, which has been described earlier in F98 cells^41^. As LAT1 overexpression has been correlated with malignant phenotype and proliferation of gliomas, our in-house developed AA could possibly demonstrate value for guiding biopsy, diagnosing primary brain tumor, tumor grading, directing radiotherapy, or even for discriminating between tumor recurrence and radionecrosis post-therapy^42,43^.

In a second set of *in vitro* experiments, the K_m_ was determined by means of a concentration dependent assay. The results show a saturable transport consistent with Michaelis Menten kinetics. The K_i_ of 2-[^18^F]FELP and [^18^F]FET did not differ significantly from their K_m_ values. The K_i_ value of a competitive inhibitor of a transport process of a substrate is equal to the K_m_ value for transport of the inhibitor by the same process. Therefore, the determination of K_i_ values can be particularly useful if no radioactive labeled compound is available^44^.

Using an *in vivo* orthotopic tumor model, the uptake of different AA tracers were compared with [^18^F]FDG uptake characteristics. As described in literature, the AA tracers benefit from a lower background signal, resulting in increased tumor-to-background values^3^. However, no significant differences could be observed between 2-[^18^F]FELP and [^18^F]FET. The elevated uptake of both tracers in the GB model could be enhanced by BBB breakdown^45^. We hypothesize that the uptake in low grade glioma, where the BBB is intact and the LAT1 expression is less distinct, would give rise to different time activity curves as seen by Galldiks *et al*.^46^. Unfortunately, preclinical low grade glioma models are not widely available^47^.

In summary, 2-[^18^F]FELP has a better affinity and selectivity for the LAT1 transporter compared to [^18^F]FET, decreasing non-specific tumor uptake. As LAT1 overexpression has been correlated with malignant phenotype and proliferation of gliomas, this new AA tracer could possibly demonstrate value for guiding biopsy, diagnosing primary brain tumor, tumor grading, directing radiotherapy, or even for discriminating between tumor recurrence and radionecrosis after initial therapy^42,43^.

## Conclusion

In this study we synthesized six fluoroalkyl substituted phenylalanine analogues, of which four have not been described before. The affinity of the d-enantiomers for the LAT1 transporter in F98 GB cells was lower than the affinity of the l-enantiomers. **2-FELP** exhibits the best affinity for the F98 GB cells. Radiolabeling was performed on a Synthra RN plus module, following the same straightforward method as for [^18^F]FET. *In vitro* uptake and inhibition experiments showed that 2-[^18^F]FELP is predominantly transported by the LAT1 transporter, decreasing non-specific uptake. No significantly different TBR_mean_, TBR_max_, SUV_mean_ tumor and SUV_max_ tumor values could be observed in comparison to [^18^F]FET in the F98 GB rat model. However, in contrast to [^18^F]FDG PET, higher TBRs were found. Based on our results, 2-[^18^F]FELP is a promising new PET tracer for glioma or LAT1 imaging.

## Methods

### General

Reagents and solvents of analytical grade have been obtained from standard commercial sources and were used directly without further purification. Moisture sensitive reactions were performed under an argon atmosphere. Reactions were carried out at ambient temperature, unless specified in the text. For analytical TLC, pre-coated F254 aluminum plates (Machery-Nagel®) were employed, which have been visualized by UV and developed with sulfuric acid-anisaldehyde spray/charring. For purification, flash column chromatography was performed using Davisil® (40–63 µm) silica or a Reveleris X2 (Grace/Büchi) automated Flash unit with pre-packed cartridges. Exact mass was analyzed by means of a Waters LCT Premier XE™ Time of Flight (ToF) mass spectrometer with a standard electrospray (ESI) and modular Lockspray™ interface. Samples dissolved in an appropriate solvent system and infused in a MeCN/water (1:1) +0.1% formic acid mixture at 100 µL/min. NMR spectra were recorded on a Varian Mercury 300 MHz spectrometer. Chemical shifts (δ) are given in ppm and spectra are referenced to the residual solvent peak signal, with coupling constants (*J*) expressed in Hz. For ^19^F-NMR, signals were referenced to the lock resonance frequency according to IUPAC referencing with CFCl_3_ set to 0 ppm. Optical rotations were measured employing a Perkin Elmer 241 Polarimeter.

Chiral HPLC was performed using the specified chiral column (*vide infra*) from Diacel, 250 × 4.6 mm, particle size 5 µm. Column temperature was 35 °C. Runs were performed isocratically with a mixture of EtOH(abs.)/*n*-hexane as specified below, with a run length of 30 min at a flow rate of 1 mL/min.

Alternatively, chiral HPLC was performed (compounds **32** & **33**, including 2-[^18^F]FELP) employing an Astec® Chirobiotic-T column (5 µm, 125 × 4.6 mm), operated at ambient temperature, with a mobile phase consisting of EtOH/water (8/2) at a flow rate of 1 mL/min.

Chiral capillary electrophoresis (compounds **15** & **16** and **17** & **18**) was performed using a 64.5 cm capillary (50 µm diameter). The electrolyte used consisted of 25 mM phosphate buffer pH 2.5 + 5% HS-gammaCD chiral modifier. Analysis time was 40 min at −20 kV.

### Experimental procedures & compound characterization data

#### 1-(*tert*-butyl)-4-(4, 5, 6, 7-tetrachloro-1, 3-dioxoisoindolin-2-yl)-(*tert*-butoxycarbonyl)-l-aspartate (1)

**Boc-L-aspartic acid 4-*tert-*butyl ester** (0.29 g, 1.0 mmol, 1 eq.) was dissolved in anhydrous DCM (10 mL, 10 mL/mmol SM). Then, DMAP (0.0012 g, 0.10 mmol, 0.1 eq.) and *N*-hydroxytetrachlorophthalimide (0.31 g, 1.0 mmol, 1 eq.) were added, followed by DIC (0.175 mL, 1.10 mmol, 1.1 eq.). The resulting suspension was stirred under argon for approximately 2 h. Then, the mixture was filtered and evaporated. Purification by column chromatography (0 → 10% EA/PET) gave **1** (0.34 g, 0.59 mmol) as a white solid in 59% yield. ^1^H-NMR (300 MHz, CDCl_3_) δ: 1.46 (s, 9H, *t*Bu), 1.47 (s, 9H, *t*Bu), 3.23 (dd, *J* = 17.4, 4.8 Hz, 1H, CH_2_), 3.32 (dd, *J* = 17.4, 4.8 Hz, 1H, CH_2_), 4.57 (dt, *J* = 7.2, 4.8 Hz, 1H, CH), 5.47 (d, *J* = 7.5 Hz, 1H, NH). ^13^C NMR (75 MHz, CDCl_3_) δ: 27.9 (t-Bu, CH_3_), 28.4 (*t*Bu, CH_3_), 34.3 (CH_2_), 50.3 (CHNH), 80.5 (*t*Bu-C-(CH_3_)_3_), 83.6 (*t*Bu-C-(CH_3_)_3_), 124.8, 130.7, 141.3, 155.5, 157.3, 167.2, 168.7. HRMS (ESI): calculated for C_21_H_23_Cl_4_N_1_O_8_ ([M + H]^+^): 571.0203, found: 571.0184. [α]^20^_D_ = +37.76 (*c* 1.17, CHCl_3_).

#### Synthesis of 1-(*tert*-butyl) 4-(4, 5, 6, 7-tetrachloro-1, 3-dioxoisoindolin-2-yl) (*tert*-butoxycarbonyl)-d-aspartate (2)

According to the procedure described for compound **1**; **Boc-D-aspartic acid 4-*tert*-butyl ester** (0.87 g, 3.0 mmol) was transformed into **2** (1.2 g, 2.1 mmol) as a white solid in 70% yield. ^1^H-NMR (300 MHz, CDCl_3_) δ: 1.46 (s, 9H, *t*Bu), 1.47 (s, 9H, *t*Bu), 3.23 (dd, *J* = 17.4, 4.8 Hz, 1H, CH_2_), 3.32 (dd, *J* = 17.4, 4.8 Hz, 1H, CH_2_), 4.56 (dt, *J* = 7.2, 4.8 Hz, 1H, CH), 5.46 (d, *J* = 7.5 Hz, 1H, NH). ^13^C NMR (75 MHz, CDCl_3_) δ: 27.9 (*t*Bu, CH_3_), 28.4 (*t*Bu, CH_3_), 34.3 (CH_2_), 50.3 (CHNH), 80.5 (*t*Bu-C-(CH_3_)_3_), 83.6 (*t*Bu-C-(CH_3_)_3_), 124.8, 130.7, 141.3, 155.4, 157.3, 167.2, 168.7. HRMS (ESI): calculated for C_21_H_23_Cl_4_N_1_O_8_ ([M + H]^+^): 571.0203, found: 571.0234. [α]^20^_D_ = −39.07 (*c* 1.29, CHCl_3_).

#### *tert*-butyl-(S)-2-((*tert*-butoxycarbonyl)amino)-3-(4-(2-((*tert*-butyldimethylsilyl)oxy)ethyl)phenyl)propanoate (3 – l-type)

**1** (0.477 g, 0.833 mmol, 1 eq.) and **21** (0.7 g, 2.5 mmol, 3 eq.) were placed in a 100 mL round bottom flask, equipped with a stir bar. The flask was evacuated and refilled with argon, three times. Then, anhydrous 1, 4-dioxane (33.8 mL, 40 mL/mmol SM) and Et_3_N (1.16 mL, 8.33 mmol, 10 eq.) were added. The resulting mixture was stirred at ambient temperature until a homogeneous solution was obtained (~5 to 10 min). Then, the flask was transferred into a pre-heated oil bath at 75 °C and stirred for another 5 min, after which the catalyst solution (3.4 mL, 4 mL/mmol SM) was added. The resulting solution was stirred at 75 °C overnight. After cooling to ambient temperature, the solvents were removed *in vacuo*, and the residue taken up in EA/water. The layers were separated, and the water layer extracted twice with EA. The organic layers were combined, dried over Na_2_SO_4_, filtered and evaporated. The residue was purified by column chromatography (0 → 10% EA/PET) to give **3** (0.185 g, 0.386 mmol) as a yellowish oil in 46% yield. ^1^H-NMR (300 MHz, CDCl_3_) δ: −0.02 (s, 6H, Si-CH_3_), 0.87 (s, 9H, Si-*t*Bu-CH_3_), 1.42 (s, 18H, *t*Bu), 2.78 (t, *J* = 7.2 Hz, 2H, PhCH_2_CH_2_OTBS), 3.02 (d, *J* = 5.7 Hz, 2H, PhCH_2_CHNH), 3.77 (t, *J* = 7.2 Hz, 2H, PhCH_2_CH_2_OTBS), 4.39–4.45 (m, 1H, CHNH), 4.94 (d, *J* = 7.8 Hz, 1H, NH), 7.06–7.13 (m, 4H, H_Phe_). ^13^C-NMR (75 MHz, CDCl_3_) δ: −5.2 (2C, Si-CH_3_), 18.5 (Si-C-(CH_3_)_3_), 26.1 (3C, Si-C-(CH_3_)_3_), 28.1 (3C, *t*Bu), 28.5 (3C, *t*Bu), 38.1 (Ph-CH_2_CH), 39.4 (PhCH_2_CH_2_OTBS), 55.0 (CHNH), 64.7 (PhCH_2_CH_2_OTBS), 79.7 (*t*Bu-C-(CH_3_)_3_), 82.1 (*t*Bu-C-(CH_3_)_3_), 129.4 (2C), 129.5 (2C), 134.2, 137.9, 155.2 (C=O_Boc_), 171.1 (C=O_ester_). HRMS (ESI): calculated for C_26_H_46_NO_5_Si ([M + H]^+^): 480.3140, found: 480.3138. [α]^20^_D_ = +26.78 (*c* 0.30, CHCl_3_).

Chiral HPLC: Chiralpak IA, eluent: *n*-Hexane/EtOH(abs.) (97/3), retention time: 5.36 min. *ee*: 100%.

Preparation of the catalyst solution (NiCl_2_.6H_2_O; 4, 4-di-*t*-butyl-bipyridine 0.05 M in DMF) was performed as has been described in literature^30^.

#### tert-butyl-(R)-2-((tert-butoxycarbonyl)amino)-3-(4-(2-((tert-butyldimethylsilyl)oxy)ethyl)phenyl)propanoate (4 – d-type)

According to the procedure described for **3**; **2** (0.41 g, 0.71 mmol) was transformed into **4** (0.31 g, 0.65 mmol) as a colorless oil in 46% yield. ^1^H-NMR (300 MHz, CDCl_3_) δ: −0.02 (s, 6H, Si-CH_3_), 0.87 (s, 9H, Si-*t*Bu-CH_3_), 1.41 (s, 9H, *t*Bu), 1.42 (s, 9H, *t*Bu), 2.78 (t, *J* = 7.2 Hz, 2H, PhCH_2_CH_2_OTBS), 3.02 (d, *J* = 5.7 Hz, 2H, PhCH_2_CH), 3.77 (t, *J* = 7.2 Hz, 2H, PhCH_2_CH_2_OTBS), 4.42 (dd, *J* = 13.8, 6.3 Hz, 1H, CHNH), 4.94 (d, *J* = 8.1 Hz, 1H, NHBoc), 7.05–7.13 (m, 4H, H_Phe_). ^13^C-NMR (75 MHz, CDCl_3_) δ: − 5.3 (2C, Si-CH_3_), 18.5 (Si-C-(CH_3_)_3_), 26.1 (3C, Si-C-(CH_3_)_3_), 28.1 (3C, *t*Bu), 28.5 (3C, *t*Bu), 38.1 (Ph-CH_2_CH), 39.4 (PhCH_2_CH_2_OTBS), 55.0 (CHNH), 64.7 (PhCH_2_CH_2_OTBS), 79.7 (*t*Bu-C-(CH_3_)_3_), 82.1 (*t*Bu-C-(CH_3_)_3_), 129.3 (2C), 129.5 (2C), 134.2, 137.8, 155.2 (C=O_Boc_), 171.1 (C=O_ester_). HRMS (ESI): calculated for C_26_H_46_N_1_O_5_Si ([M + H]^+^): 480.3140, found: 480.3152. [α]^20^_D_ = − 25.00 (*c* 0.56, CHCl_3_).

Chiral HPLC: Chiralpak IA, eluent: *n*-Hexane/EtOH(abs.) (97/3), retention time: 4.95 min. *ee*: 96.3%.

#### *tert*-butyl-(S)-2-((tert-butoxycarbonyl)amino)-3-(3-(2-hydroxyethyl)phenyl)propanoate (5 – l-type)

According to the procedure as described for **3**; **1** (0.57 g, 1.0 mmol) was transformed into **5** (0.28 g, 0.59 mmol) as a colorless oil in 59% yield.

^1^H-NMR (300 MHz, CDCl_3_) δ: -0.01 (s, 6H, Si-(CH_3_)_2_), 0.87 (s, 9H, Si-*t*Bu-CH_3_), 1.40 (s, 9H, *t*Bu), 1.42 (s, 9H, *t*Bu), 2.78 (t, *J* = 7.2 Hz, 2H, PhCH_2_CH_2_OTBS), 3.02 (d, *J* = 6.0 Hz, 2H, PhCH_2_CH), 3.77 (t, *J* = 7.5 Hz, 2H, PhCH_2_CH_2_OTBS), 4.40–4.57 (m, 1H, CHNHBoc), 4.96 (d, *J* = 7.8 Hz, 1H, NHBoc), 6.99 (s, 1H, Ph-H), 7.02–7.09 (m, 2H, H_Phe_), 7.19 (t, *J* = 7.5 Hz, 1H, H_Phe_).^13^C-NMR (75 MHz, CDCl_3_) δ: −5.2 (2C, Si-CH_3_), 18.5 (Si-C-(CH_3_)_3_), 26.1 (3C, Si-C-(CH_3_)_3_), 28.1 (*t*Bu, CH_3_), 28.5 (*t*Bu, CH_3_), 38.4 (PhCH_2_CH), 39.7 (PhCH_2_CH_2_OTBS), 54.9 (CHNH), 64.7 (PhCH_2_CH_2_OTBS), 79.7 (*t*Bu-C-(CH_3_)_3_), 82.1 (*t*Bu-C-(CH_3_)_3_), 127.4, 127.8, 128.3, 130.5, 136.4, 139.2, 155.2 (C=O_Boc_), 171.1 (C=O_ester_). HRMS (ESI): calculated for C_26_H_46_NO_5_Si ([M + H]^+^): 480.3140, found: 480.3142. [α]^20^_D_ = +26.76 (*c* 0.36, CHCl_3_).

#### *tert*-butyl-(R)-2-((*tert*-butoxycarbonyl)amino)-3-(3-(2-hydroxyethyl)phenyl)propanoate (6 – d-type)

According to the procedure as described for **3**; **2** (0.57 g, 1.0 mmol) was transformed into **6** (0.16 g, 0.34 mmol) as a colorless oil in 34% yield.

^1^H-NMR (300 MHz, CDCl_3_) δ: −0.01 (s, 6H, Si-(CH_3_)_2_), 0.87 (s, 9H, Si-*t*Bu -CH_3_), 1.40 (s, 9H, *t*Bu), 1.42 (s, 9H, *t*Bu), 2.78 (t, *J* = 7.5 Hz, 2H, PhCH_2_CH_2_OTBS), 3.03 (d, *J* = 6.0 Hz, 2H, PhCH_2_CH), 3.78 (t, *J* = 7.2 Hz, 2H, PhCH_2_CH_2_OTBS), 4.40–4.47 (m, 1H, CHNH), 4.95 (d, *J* = 7.8 Hz, 1H, NH), 6.95 (s, 1H, H_Phe_), 7.02–7.09 (m, 2H, H_Phe_), 7.19 (t, *J* = 7.8 Hz, 1H, H_Phe_). ^13^C-NMR (75 MHz, CDCl_3_) δ: −5.2 (2C, Si-CH_3_), 18.5 (Si-C-(CH_3_)_3_), 26.1 (3C, Si-C-(CH_3_)_3_), 28.1 (*t*Bu), 28.5 (3C, *t*Bu), 38.5 (PhCH_2_CH), 39.7 (PhCH_2_CH_2_OTBS), 54.9 (CHNH), 64.7 (PhCH_2_CH_2_OTBS), 79.7 (*t*Bu-C-(CH_3_)_3_), 82.1 (*t*Bu-C-(CH_3_)_3_), 127.5, 127.8, 128.3, 130.5, 136.4, 139.2, 155.2 (C=O_Boc_), 171.1 (C=O_ester_). HRMS (ESI): calculated for C_26_H_46_NO_5_Si ([M + H]^+^): 480.3140, found: 480.3183. [α]^20^_D_ = +24.07 (*c* 0.26, CHCl_3_).

#### *tert*-butyl-(S)-2-((*tert*-butoxycarbonyl)amino)-3-(4-(2-hydroxyethyl)phenyl)propanoate (7 – l-type)

**3** (0.098 g, 0.204 mmol) was dissolved in THF (3 mL, 10 mL/mmol SM) and TBAF in THF (1.0 M, 0.23 mL, 0.23 mmol, 1.1 eq.) was added. The resulting mixture was stirred for approximately 1H, and then sat. aq. NH_4_Cl solution was added together with EA. The layers were separated; the water layer extracted twice more with EA. The organic layers were combined and dried over Na_2_SO_4_, filtered and evaporated. The residue was purified by column chromatography (0 → 35% EA/PET) to give **7** (0.062 g, 0.17 mmol) as a colorless oil in 83% yield. ^1^H-NMR (300 MHz, CDCl_3_) δ: 1.41 (s, 18H, *t*Bu), 1.62 (br. s, 1H, OH), 2.84 (t, *J* = 6.6 Hz, 2H, PhCH_2_CH_2_OH), 3.00–3.04 (m, 2H, PhCH_2_CH), 3.84 (t, *J* = 6.6 Hz, 2H, PhCH_2_CH_2_OH), 4.43 (dd, *J* = 14.1, 6.6 Hz, 1H, PhCH_2_CH), 4.98 (d, *J* = 8.1 Hz, 1H, NH), 7.10–7.26 (m, 4H, H_Phe_). HRMS (ESI): calculated for C_20_H_32_NO_5_ ([M + H]^+^): 366.2275, found: 366.2284. [α]^20^_D_ = +42.23 (*c* 0.85, CHCl_3_). Spectral data matched those reported in literature^25^.

#### *tert*-butyl-(R)-2-((*tert*-butoxycarbonyl)amino)-3-(4-(2-hydroxyethyl)phenyl)propanoate (8 – d-type)

According to the procedure as described for **7**; **4** (0.326 g, 0.7 mmol) was transformed into **8** (0.196 g, 0.536 mmol) as a colorless oil in 77% yield. ^1^H-NMR (300 MHz, CDCl_3_) δ: 1.41 (s, 18H, *t*Bu CH_3_), 2.84 (t, *J* = 6.6 Hz, 2H, PhCH_2_CH_2_OH), 2.96–3.08 (m, 2H, PhCH_2_CH), 3.84 (t, *J* = 6.6 Hz, 2H, PhCH_2_CH_2_OH), 4.39–4.46 (m, 1H, CHNH), 4.98 (d, *J* = 8.1 Hz, 1H, NH), 7.10–7.17 (m, 4H, H_Phe_). HRMS (ESI): calculated for C_20_H_32_NO_5_ ([M + H]^+^): 366.2275, found: 366.2282. Spectral data matched those reported in literature^25^.

#### *tert*-butyl (S)-2-((*tert*-butoxycarbonyl)amino)-3-(3-(2-hydroxyethyl)phenyl)propanoate (9 – l-type)

According to the procedure as described for **7**; **5** (0.547 g, 1.14 mmol) was transformed into **9** (0.29 g, 0.80 mmol) as a colorless oil in 71% yield. ^1^H-NMR (300 MHz, CDCl_3_) δ: 1.37 (s, 9H, *t*Bu), 1.42 (s, 9H, *t*Bu), 1.81 (br. s, 1H, OH), 2.82 (t, *J* = 6.3 Hz, 2H, PhCH_2_CH_2_OH), 2.92 (dd, *J* = 13.8, 6.9 Hz, 1H, PhCH_2_CH), 3.10 (dd, *J* = 13.8, 6.0 Hz, 1H, PhCH_2_CH), 3.76–3.90 (m, 2H, PhCH_2_CH_2_OH), 4.47 (dd, *J* = 14.4, 6.9 Hz, 1H, CHNH), 4.98 (d, *J* = 8.7 Hz, 1H, NH), 7.01–7.09 (m, 3H, H_Phe_), 7.22 (t, *J* = 7.8 Hz, 1H, H_Phe_). ^13^C-NMR (75 MHz, CDCl_3_) δ: 28.1 (3C, *t*Bu), 28.4 (3C, *t*Bu), 39.0 (PhCH_2_CH), 39.3 (PhCH_2_CH_2_OH), 55.0 (PhCH_2_CH), 63.7 (PhCH_2_CH_2_OH), 79.8 (*t*Bu-C-(CH_3_)_3_), 82.2 (*t*Bu-C-(CH_3_)_3_), 127.6, 128.0, 128.7, 130.5, 136.8, 138.8, 155.1 (C=O_Boc_), 171.2 (C=O_ester_). HRMS (ESI): calculated for C_20_H_32_NO_5_ ([M + H]^+^): 366.2275, found: 366.2268. [α]^20^_D_ = +25.68 (*c* 0.51, CHCl_3_).

Chiral HPLC: Chiralpak IB, eluent: *n*-Hexane/EtOH(abs.) (97/3), retention time: 12.39 min. *ee*: 99.6%.

#### *tert*-butyl-(R)-2-((*tert*-butoxycarbonyl)amino)-3-(3-(2-hydroxyethyl)phenyl)propanoate (10 – d-type)

According to the procedure as described for **7**; **6** (0.26 g, 0.54 mmol) was transformed into **10** (0.15 g, 0.40 mmol) as a colorless oil in 73% yield. ^1^H - NMR (300 MHz, CDCl_3_) δ: 1.37 (s, 9H, *t*Bu), 1.44 (s, 9H, *t*Bu), 1.94 (br. s, 1H, OH), 2.82 (t, *J* = 6.6 Hz, 2H, Ph-CH_2_CH), 2.92 (dd, *J* = 13.8, 6.9 Hz, 1H, PhCH_2_CH), 3.09 (dd, *J* = 13.8, 5.7 Hz, 1H, PhCH_2_CH), 3.75–3.89 (m, 2H, PhCH_2_CH_2_OH), 4.46 (dd, *J* = 14.4, 6.9 Hz, 1H, CHNH), 4.99 (d, *J* = 8.1 Hz, 1H, NH), 7.00–7.09 (m, 3H, H_Phe_), 7.21 (t, *J* = 7.8 Hz, 1H, H_Phe_). ^13^C-NMR (75 MHz, CDCl_3_) δ: 28.1 (3C, *t*Bu), 28.4 (3C, *t*Bu), 38.9 (PhCH_2_CH), 39.3 (PhCH_2_CH_2_OH), 55.0 (PhCH_2_CH), 63.7 (PhCH_2_CH_2_OH), 79.8 (*t*Bu-C-(CH_3_)_3_), 82.2 (*t*Bu-C-(CH_3_)_3_), 127.6, 127.9, 128.6, 130.5, 136.7, 138.8, 155.1 (C=O_Boc_), 171.1 (C=O_ester_). HRMS (ESI): calculated for C_20_H_32_N_1_O_5_ ([M + H]^+^): 366.2275, found: 366.2281. [α]^20^_D_ = − 26.79 (*c* 0.52, CHCl_3_).

Chiral HPLC: Chiralpak IB, eluent: *n*-Hexane/EtOH(abs.) (97/3), retention time: 10.52 min. *ee*: 98.3%.

#### *tert*-butyl-(S)-2-((*tert*-butoxycarbonyl)amino)-3-(4-(2-fluoroethyl)phenyl)propanoate (11 – l-type)

**7** (0.18 g, 0.49 mmol, 1 eq.) was dissolved in anhydrous DCM (2.5 mL, 10 mL/mmol SM) under argon and cooled in an ice bath at 0 °C. Then, DAST (0.20 mL, 1.5 mmol, 3 eq.) was added dropwise and the resulting solution stirred at ambient temperature for 3–4 h. Then, the mixture was cooled to 0 °C in an ice bath and aq. sat. NaHCO_3_ solution was added dropwise. After the gas evolution ceased, the mixture was transferred into a separatory funnel and more sat. aq. NaHCO_3_ solution was added. The layers were separated, and the water layer was extracted twice more with DCM. The organic layers were combined, dried over Na_2_SO_4_, filtered and evaporated till dryness. The resulting mixture was purified by column chromatography (0 → 12% EA/PET) to give **11** (0.076 g, 0.21 mmol) as a colorless oil in 42% yield. ^1^H-NMR (300 MHz, CDCl_3_) δ: 1.40 (s, 9H, *t*Bu), 1.42 (s, 9H, *t*Bu), 2.94 (t, *J* = 6.6 Hz, 1H, PhCH_2_CH_2_F)^1^*, 3.00–3.06 (m, 3H, PhCH_2_CH_2_F; PhCH_2_CH), 4.43 (q, *J* = 6.9 Hz, 1H, PhCH_2_CH), 4.60 (dt, *J* = 47.1, 6.6 Hz, 2H, PhCH_2_CH_2_F), 4.98 (d, *J* = 6.9 Hz, 1H, NH), 7.10–7.17 (m, 4H, H_Phe_). ^19^F-NMR (282 MHz, CDCl_3_) δ: −214.98 – −215.48 (m, 1F). Spectral data are in accordance with literature values^25^.

#### *tert*-butyl-(R)-2-((*tert*-butoxycarbonyl)amino)-3-(4-(2-fluoroethyl)phenyl)propanoate (12 – d-type)

According to the procedure described for **11**; **8** (0.174 g, 0.476 mmol) was transformed into **12** (0.065 g, 0.18 mmol) as a colorless oil in 37% yield. ^1^H-NMR (300 MHz, CDCl_3_) δ: 1.40 (s, 9H, *t*Bu), 1.42 (s, 9H, *t*Bu), 2.98 (dt, *J* = 23.1, 6.6 Hz, 2H, PhCH_2_CH_2_F), 3.00–3.04 (m, 2H, PhCH_2_CH), 4.43 (dd, *J* = 13.8, 6.3 Hz, 1H, PhCH_2_CH), 4.60 (dt, *J* = 47.1, 6.9 Hz, 2H, PhCH_2_CH_2_F), 4.98 (d, *J* = 8.4 Hz, 1H, NH), 7.10–7.17 (m, 4H, H_Phe_). ^19^F-NMR (282 MHz, CDCl_3_) δ): −214.98 – −215.48 (m, 1 F). Spectral data matched those reported in literature^25^.

#### *tert*-butyl-(S)-2-((*tert*-butoxycarbonyl)amino)-3-(3-(2-(tosyloxy)ethyl)phenyl)propanoate (13 – l-type)

According to the procedure described for **11**; **9** (0.235 g, 0.634 mmol) was transformed into **13** (0.117 g, 0.319 mmol) as a colorless oil in 50% yield. ^1^H-NMR (300 MHz, CDCl_3_) δ: 1.40 (s, 9H, *t*Bu), 1.42 (s, 9H, *t*Bu), 2.98 (dt, *J* = 23.1, 6.6 Hz, 2H, PhCH_2_CH_2_F), 3.03 (dd, *J* = 6.3, 4.2 Hz, 2H, PhCH_2_CHNH), 4.41–4.48 (m, 1H, PhCH_2_CH), 4.61 (dt, *J* = 47.1, 6.6 Hz, 2H, PhCH_2_CH_2_F), 4.98 (d, *J* = 7.8 Hz, 1H, NH), 7.04–7.12 (m, 3H, H_Phe_), 7.22 (d, *J* = 7.8 Hz, 1H, H_Phe_). ^19^F-NMR (282 MHz, CDCl_3_) δ: −215.06 (tt, *J* = 46.8, 22.8 Hz, 1 F). ^13^C-NMR (75 MHz, CDCl_3_) δ: 28.1 (3C, *t*Bu), 28.5 (3C, *t*Bu), 37.0 (d, *J* = 20.6 Hz, 1C, PhCH_2_CH_2_F), 38.6 (PhCH_2_CHNHBoc), 54.9 (PhCH_2_CHNH), 79.8 (*t*Bu-C-(CH_3_)_3_), 82.2 (t-Bu-C-(CH_3_)_3_), 84.1 (d, *J* = 168.2 Hz, 1C, PhCH_2_CH_2_F), 127.6, 128.0, 128.7, 130.4, 136.8, 137.2 (d, *J* = 5.7 Hz, 1C, Ph-C-CH_2_CH_2_F), 155.2 (C=O_Boc_), 171.1 (C=O_ester_). HRMS (ESI): calculated for C_20_H_31_FNO_4_ ([M + H]^+^): 368.2232, found: 368.2231. [α]^20^_D_ = +40.83 (*c* 0.72, CHCl_3_).

#### *tert*-butyl-(R)-2-((tert-butoxycarbonyl)amino)-3-(3-(2-hydroxyethyl)phenyl)propanoate (14 – d-type)

According to the procedure described for **11**; **10** (0.146 g, 0.399 mmol) was transformed into **14** (0.042 g, 0.11 mmol) as a colorless oil in 29% yield.

^1^H-NMR (300 MHz, CDCl_3_) δ: 1.40 (s, 9H, *t*Bu), 1.42 (s, 9H, *t*Bu), 2.95 (dt, *J* = 23.1, 6.6 Hz, 2H, PhCH_2_CH_2_F), 3.01–3.05 (m, 2H, PhCH_2_CHNH), 4.45 (dd, *J* = 14.1, 6.3 Hz, 1H, PhCH_2_CHNH), 4.61 (dt, *J* = 47.1, 6.6 Hz, 2H, PhCH_2_CH_2_F), 4.99 (d, *J* = 8.1 Hz, 1H, NH), 7.04–7.12 (m, 3H, H_Phe_), 7.22 (d, *J* = 7.2 Hz, 1H, H_Phe_). ^19^F-NMR (282 MHz, CDCl_3_) δ: −215.02 (tt, *J* = 46.8, 22.8 Hz, 1 F). ^13^C-NMR (75 MHz, CDCl_3_) δ: 28.1 (3C, *t*Bu), 28.5 (3C, *t*Bu), 37.0 (d, *J* = 20.63 Hz, 1C, PhCH_2_CH_2_F), 38.5 (PhCH_2_CHNH), 54.9 (PhCH_2_CHNH), 79.8 (*t*Bu-C-(CH_3_)_3_), 82.2 (*t*Bu-C-(CH_3_)_3_), 84.1 (d, *J* = 168.3 Hz, 1C, PhCH_2_CH_2_F), 127.6, 128.0, 128.7, 130.4, 136.8, 137.2 (d, *J* = 5.7 Hz, 1C, Ph-C-CH_2_CH_2_F), 155.2 (C=O_Boc_), 171.1 (C=O_ester_). HRMS (ESI): calculated for C_20_H_31_FN_1_O_4_ ([M + H]^+^): 368.2232, found: 368.2228. [α]^20^_D_ = −40.24 (*c* 0.4, CHCl_3_).

#### (S)-2-amino-3-(4-(2-fluoroethyl)phenyl)propanoic acid (15 – l-type – 4FELP)

**11** (0.076 g, 0.21 mmol) was dissolved in DCM (3.0 mL) after which TFA (3.0 mL) was added. The resulting solution was stirred at ambient temperature for approximately 5 hours, after which it was evaporated till dryness. Next, the residue was co-evaporated with MeOH three times. Then, the residue was dissolved in ~2 mL of MeOH and the pH was adjusted to pH 6–7, with diluted aq. NH_3_ solution, after which a white precipitate formed. The mixture was refrigerated overnight, and then the liquid was removed via pipette aspiration. The remaining solid was washed thrice with a minimal amount of ice-cold MeOH and dried by means of oil pump high vacuum to give **15** (0.017 g, 0.081 mmol) as a white powder in 39% yield. ^1^H-NMR (300 MHz, D_2_O) δ: 3.06 (dt, *J* = 27.6, 6.3 Hz, 2H, PhCH_2_CH_2_F), 3.12 (dd, *J* = 14.4, 8.1 Hz, 1H, PhCH_2_CHNH_2_), 3.29 (dd, *J* = 14.7, 5.4 Hz, 1H, PhCH_2_CHNH_2_), 3.99 (dd, *J* = 8.1, 5.4 Hz, 1H, PhCH_2_CHNH_2_), 4.67 (t, *J* = 6.0 Hz, 1H, PhCH_2_CH_2_F), 7.29–7.37 (m, 4H, H_Phe_). ^19^F-NMR (282 MHz, D_2_O) δ: −214.75 (tt, *J* = 49.8, 29.4 Hz, 1 F). HRMS (ESI): calculated for C_11_H_15_FNO_2_ ([M + H]^+^): 212.1081, found: 212.1077.

Chiral CE: retention time: 32.6 min, *ee*: 98.5%.

1 proton signal is missing (PhCH_2_CH_2_F) as it resides under the HDO peak; therefore, the signal for δ = 4.67 ppm is calculated on the visual triplet and not on the actual doublet of triplets (dt).

#### (R)-2-amino-3-(4-(2-fluoroethyl)phenyl)propanoic acid (16 – d-type – 4-FEDP)

According to the procedure as described for **15**; **11** (0.065 g, 0.18 mmol) was transformed into **11** (0.013 g, 0.062 mmol) as a white powder in 35% yield. ^1^H-NMR (300 MHz, D_2_O) δ: 3.06 (dt, *J* = 27.6, 6.0 Hz, 2H, PhCH_2_CH_2_F), 3.12 (dd, *J* = 14.7, 8.1 Hz, 1H, PhCH_2_CH), 3.29 (dd, *J* = 14.7, 5.4 Hz, 1H, PhCH_2_CHNH_2_), 3.99 (dd, *J* = 8.1, 5.4 Hz, 1H, PhCH_2_CHNH_2_), 4.75 (dt, *J* = 47.1, 6.3 Hz, 2H, PhCH_2_CH_2_F), 7.29–7.37 (m, 4H, H_Phe_). ^19^F-NMR (282 MHz, D_2_O) δ): −216.04 (tt, *J* = 44.8, 29.1 Hz, 1 F).

Chiral CE: retention time: 28.4 min, *ee*: 88.8%.

#### (S)-2-amino-3-(3-(2-fluoroethyl)phenyl)propanoic acid.HCl salt (17 – l-type – 3-FELP)

**13** (0.114 g, 0.310 mmol) was dissolved in DCM (3.0 mL) after which TFA (3.0 mL) was added. The resulting solution was stirred at ambient temperature for approximately 5 hours. Then, the mixture was evaporated till dryness. The mixture was co-evaporated with MeOH three times. Then, the residue was neutralized with diluted aq. NH_3_ and refrigerated. As no precipitate formed; the mixture was evaporated till dryness and dissolved in water (~5 mL); and the pH adjusted to pH 6. Then, the mixture was purified by RP-flash chromatography (4 g, C-18 column, Büchi). Gradient: 100% water for 1 min, then linear gradient 0 → 20% EtOH/water in 15 min. Product containing fractions were pooled and most of the water was evaporated *in vacuo*; after which the residue was lyophilized. Then, the white powder was dissolved in water and diluted (~0.5 M) aq. HCl (~0.5 mL) was added and lyophilized again, which gave **17** (0.035 g, 0.14 mmol) as a white powder in 46% yield. ^1^H-NMR (300 MHz, D_2_O) δ: 3.06 (dt, *J* = 27.9, 6.0 Hz, 2H, PhCH_2_CH_2_F), 3.21 (dd, *J* = 14.4, 7.8 Hz, 1H, PhCH_2_CH), 3.35 (dd, *J* = 14.4, 5.7 Hz, 1H, PhCH_2_CH), 4.28 (dd, *J* = 7.8, 5.7 Hz, 1H, PhCH_2_CH), 4.75 (dt, *J* = 46.8, 6.0 Hz, 2H, PhCH_2_CH_2_F), 7.23–7.34 (m, 3H, H_Phe_), 7.41 (t, *J* = 7.5 Hz, 1H, H_Phe_). ^19^F-NMR (282 MHz, D_2_O) δ: −216.11 (tt, *J* = 46.8, 27.6 Hz, 1 F). ^13^C-NMR (75 MHz, D_2_O) δ: 35.6 (PhCH_2_CH), 35.8 (d, *J* = 19.4 Hz, 1C, PhCH_2_CH_2_F), 54.5 (PhCH_2_CH), 85.1 (d, *J* = 160.3 Hz, 1C, PhCH_2_CH_2_F), 127.6, 128.4, 129.3, 129.9, 134.5, 138.7 (d, *J* = 4.6 Hz, 1C, Ph-C-CH_2_CH_2_F), 171.8 (C=O). HRMS (ESI): calculated for C_11_H_15_FNO_2_ ([M + H]^+^): 212.1081, found: 212.1046.

Chiral CE: retention time: 23.74 min, *ee*: 95.4%.

#### (R)-2-amino-3-(3-(2-fluoroethyl)phenyl)propanoic acid.HOAc salt (18 – d-type – 3-FEDP)

**14** (0.072 g, 0.20 mmol) was dissolved in DCM (2.0 mL) after which TFA (2.0 mL) was added. The resulting solution was stirred at ambient temperature for approximately 5 hours. Then, the mixture was evaporated till dryness. The mixture was co-evaporated with MeOH three times. Then, the residue was neutralized with diluted aq. NH_3_ and refrigerated. As no precipitate formed; the mixture was evaporated till dryness and dissolved in water (~4 mL); and the pH adjusted to pH 6. Then, the mixture was purified by RP-flash chromatography (4 g, C-18 column, Büchi). Gradient: 100% water for 1 min, then linear gradient 0 → 20% EtOH/water in 15 min. Product containing fractions were pooled and most of the water was evaporated *in vacuo*; after which the residue was lyophilized. Then, the white powder was dissolved in water and diluted (~0.5 M) aq. HOAc (~0.5 mL) was added and lyophilized again, which gave **18** (0.025 g, 0.092 mmol) as a white powder in 46% yield. ^1^H-NMR (300 MHz, D_2_O) δ: 1.97 (s, 3H, OAc), 3.06 (dt, *J* = 27.9, 6.3 Hz, 2H, PhCH_2_CH_2_F), 3.12 (dd, *J* = 15.0, 5.7 Hz, 1H, PhCH_2_CH), 3.29 (dd, *J* = 14.7, 5.4 Hz, 1H, PhCH_2_CH), 4.00 (dd, *J* = 8.1, 5.1 Hz, 1H, PhCH_2_CH), 4.75 (dt, *J* = 47.1, 6.3 Hz, 2H, PhCH_2_CH_2_F), 7.21–7.32 (m, 3H, H_Phe_), 7.40 (t, *J* = 7.5 Hz, 1H, H_Phe_). ^19^F-NMR (282 MHz, D_2_O) δ: −215.90 (tt, *J* = 46.8, 27.6 Hz, 1 F). ^13^C NMR (75 MHz, D_2_O) δ: 22.5 (CH_3_-acetate), 35.8 (d, *J* = 19.5 Hz, 1C, PhCH_2_CH_2_F), 36.2 (PhCH_2_CH), 55.9 (PhCH_2_CH), 85.1 (d, *J* = 161.48 Hz, 1C, PhCH_2_CH_2_F), 127.5, 128.2, 129.2, 129.8, 135.4, 138.6 (d, *J* = 4.6 Hz, 1C, Ph-C-CH_2_CH_2_F), 173.8 (C=O), 180.2 (C=O, acetate). HRMS (ESI): calculated for C_11_H_15_FNO_2_ ([M + H]^+^): 212.1081, found: 212.1079.

Chiral CE: retention time: 19.3 min, *ee*: 93.8%.

#### Synthesis of 4-bromophenethoxy(*t*-butyl)dimethylsilane (19) 2-(4-bromophenyl)-ethanol

(2.0 g, 10 mmol, 1 eq.) was dissolved in 20 mL anhydrous DMF (2 mL/mmol SM) under argon. Then, imidazole (1.7 g, 25 mmol, 2.5 eq.) and TBSCl (1.8 g, 12 mmol, 1.2 eq.) were added. The resulting solution was stirred at ambient temperature for 3H, after which water and diethylether were added. The layers were separated, and the water layer extracted twice with diethylether. The organic layers were combined, washed with brine and dried over Na_2_SO_4_, filtered and evaporated till dryness. The residue was purified by column chromatography (100% PET → 2.5% EA/PET) to give **19** (3.1 g, 9.8 mmol) as a colorless oil in 98% yield. ^1^H-NMR (300 MHz, CDCl_3_) δ: −0.02 (s, 6H, 2 × CH_3_), 0.87 (s, 9H, *t*Bu CH_3_), 2.77 (t, *J* = 6.9 Hz, 2H, Ph-CH_2_), 3.78 (t, *J* = 6.9 Hz, 2H, CH_2_OSi), 7.06–7.10 (m, 2H, H_Phe_), 7.38–7.41 (m, 2H, H_Phe_). Spectral data matched those reported in literature^48^.

#### Synthesis of 3-bromophenethoxy(*tert*-butyl)dimethylsilane (20)

According to the procedure as described for **19**; **3-(4-bromophenyl)-ethanol** (3.0 g, 15 mmol) was transformed into **20** (4.73 g, 15.0 mmol) as a colorless oil in quantitative yield. ^1^H-NMR (300 MHz, CDCl_3_) δ: −0.03 (s, 6H, Si-(CH_3_)_3_), 0.87 (s, 9H, *t*Bu), 2.78 (t, *J* = 6.9 Hz, 2H, PhCH_2_CH_2_O), 3.79 (t, *J* = 6.9 Hz, 2H, PhCH_2_CH_2_O), 7.12–7.17 (m, 2H, H_Phe_), 7.32–7.35 (m, 1H, H_Phe_), 7.37–7.38 (m, 1H, H_Phe_). ^13^C-NMR (75 MHz, CDCl_3_) δ: −5.32 (2C, Si-CH_3_), 18.45 (Si-C-(CH_3_)_3_), 26.04 (3C, *t*Bu), 39.26 (PhCH_2_CH_2_O), 64.14 (PhCH_2_CH_2_O), 122.35, 127.98, 129.32, 129.86, 132.44, 141.89. Spectral data matched those reported in literature^31^.

#### Synthesis of (4-(2-((*tert*-butyldimethylsilyl)oxy)ethyl)phenyl)boronic acid (21)

**19** (1.9 g, 6.0 mmol, 1 eq.) was dissolved in anhydrous THF (30 mL, 5 mL/mmol SM) in a flame-dried round bottom flask under argon. The resulting solution was cooled to −78 °C. Then, *n*BuLi in hexanes (1.6 M, 4.5 mL, 7.2 mmol, 1.2 eq.) was added dropwise (0.3 mL/min) at −78 °C. After the addition was complete the resulting solution was kept at –78 °C for 1H, after which B(OMe)_3_ (1.0 mL, 9.0 mmol, 1.5 eq.) was added all at once at −78 °C. The solution was stirred at −78 °C for 30 min, after which the cooling bath was removed, and the reaction stirred at ambient temperature for another 2 h. Then, aq. sat. NH_4_Cl solution (18 mL, 3 mL/mmol SM) was added and stirring continued for 5 min. The organic solvents were removed *in vacuo*, and DCM was added. The pH was adjusted till pH 1 with 3 M aq. HCl solution, and the layers were separated. The water layer was extracted twice with DCM. The organic layers were combined, dried over Na_2_SO_4_, filtered and evaporated till dryness. The residue was purified by column chromatography (15 → 50% EA/PET), to give **21** (0.71 g, 2.5 mmol) as a colorless oil in 42% yield. ^1^H-NMR (300 MHz, CDCl_3_) δ: 0.00 (s, 6H, 2 × Si-CH_3_), 0.89 (s, 9H, *t*Bu), 2.91 (t, *J* = 6.9 Hz, 2H, PhCH_2_), 3.87 (t, *J* = 6.9 Hz, 2H, CH_2_-OSi), 7.35 (d, *J* = 7.8 Hz, 2H, H_Phe_), 8.15 (d, *J* = 8.1 Hz, 2H, H_Phe_). HRMS (ESI): calculated for C_15_H_26_BO_5_Si ([M + HCOO]^−^): 325.1648, found: 325.1660. Spectral data matched those reported in literature^49^.

#### (3-(2-((*tert*-butyldimethylsilyl)oxy)ethyl)phenyl)boronic acid (22)

According to the procedure as described for **21**; **20** (1.9 g, 6.0 mmol) was transformed into **22** (0.925 g, 3.30 mmol) as a colorless oil 55% yield. ^1^H-NMR (300 MHz, CDCl_3_) δ: 0.00 (s, 6H, 2 × Si-CH_3_), 0.88 (s, 9H, *t*Bu), 2.95 (t, *J* = 7.2 Hz, 2H, PhCH_2_CH_2_OTBS), 3.80 (t, *J* = 6.9 Hz, 2H, PhCH_2_CH_2_OTBS), 7.20–7.29 (m, 2H, H_Phe_), 7.30–7.35 (m, 2H, H_Phe_). Spectral data matched those reported in literature^31^.

#### *tert*-butyl (S)−3-(2-bromophenyl)-2-((*tert*-butoxycarbonyl)amino)propanoate (23 – l-type)

**(S)-3-(2-bromophenyl)-2-((*tert*-butoxycarbonyl)amino)propanoic acid** (0.69 g, 2.0 mmol, 1eq.) was dissolved in DCM (20 mL, 10 mL/mmol) under argon. Next, *tert*-butyl 2, 2, 2-trichloroacetimidate (1.3 g, 6.0 mmol, 3 eq.) was added. The resulting solution was stirred at ambient temperature overnight, after which water was added. Then, the layers were separated, and the organic layer was washed with sat. aq. NaHCO_3_ solution. The organic layer was dried over Na_2_SO_4_, filtered and evaporated. The residue was pre-adsorbed onto Celite® and purified by column chromatography (8% EA/PET); which gave **23** (0.72 g, 1.8 mmol) as an oil that solidified upon standing in 90% yield. ^1^H-NMR (300 MHz, CDCl_3_) δ: 1.37 (s, 9H, *t*Bu), 1.40 (s, 9H, *t*Bu), 3.06 (dd, *J* = 13.8, 8.7 Hz, 1H, Ph-CH_2_), 3.25 (dd, *J* = 13.3, 6.0 Hz, 1H, Ph-CH_2_), 4.51–4.59 (m, 1H, CH), 5.06 (d, *J* = 7.8 Hz, 1H, NH), 7.06–7.23 (m, 3H, H_Phe_), 7.54 (d, *J* = 7.8 Hz, 1H, H_Phe_). ^13^C-NMR (75 MHz, CDCl_3_) δ: 28.0 (t-Bu, CH_3_), 28.4 (*t*Bu, CH_3_), 39.2 (CH_2_), 54.1 (CHNH), 79.7 (*t*Bu, C-(CH_3_)_3_), 82.2 (*t*Bu, C-(CH_3_)_3_), 125.2, 127.4, 128.5, 131.5, 133.0, 136.7, 155.1 (C=O_Boc_), 171.2 (C=O_ester_). HRMS (ESI): calculated for C_18_H_27_BrNO_4_ ([M + H]^+^): 400.1118, found: 400.1133. [α]^20^_D_ = +6.03 (*c* 0.995, CHCl_3_).

#### *tert*-butyl (R)-3-(2-bromophenyl)-2-((tert-butoxycarbonyl)amino)propanoate (24 – d-type)

According to the procedure as described for **23**; **(R)-3-(2-bromophenyl)-2-((*tert*-butoxycarbonyl)amino)propanoic acid** (1.7 g, 5.0 mmol) was transformed in to **24** (1.4 g, 3.6 mmol) as a white solid in 72% yield. ^1^H-NMR (300 MHz, CDCl_3_) δ: 1.37 (s, 9H, *t*Bu), 1.40 (s, 9H, *t*Bu), 3.06 (dd, *J* = 14.1, 8.7 Hz, 1H, Ph-CH_2_), 3.25 (dd, *J* = 13.8, 6.0 Hz, 1H, Ph-CH_2_), 4.51–4.59 (m, 1H, CH), 5.06 (d, *J* = 8.4 Hz, 1H, NH), 7.07–7.12 (m, 1H, H_Phe_), 7.19–7.26 (m, 2H, H_Phe_), 7.54 (d, *J* = 8.1 Hz, 1H, H_Phe_). ^13^C-NMR (75 MHz, CDCl_3_) δ: 28.0 (*t*Bu, CH_3_), 28.4 (*t*Bu, CH_3_), 39.2 (CH_2_), 54.1 (CHNH), 79.7 (*t*Bu, C-(CH_3_)_3_), 82.2 (*t*Bu, C-(CH_3_)_3_), 125.2, 127.4, 128.5, 131.5, 133.0, 136.7, 155.1 (C=O_Boc_), 171.2 (C=O_ester_). HRMS (ESI): calculated for C_18_H_27_BrNO_4_ ([M + H]^+^): 400.1118, found: 400.1132. [α]^20^_D_ = −5.62 (*c* 1.46, CHCl_3_).

#### *tert*-butyl (S)-2-((*tert*-butoxycarbonyl)amino)-3-(2-vinylphenyl)propanoate (25 – l-type)

**23** (0.20 g, 0.50 mmol, 1 eq.), LiCl (0.065 g, 1.5 mmol, 3 eq.) and Pd(Ph_3_P)_2_Cl_2_ (0.018 g, 0.025 mmol, 0.05 eq.) were added to a flame-dried round bottom flask under argon. The air was removed and refilled with argon. This procedure was repeated for three times in total. Then, under argon, anhydrous degassed DMF (2.5 mL, 5 mL/mmol) was added, followed by vinyl-Sn(*n*Bu)_3_ (0.19 mL, 0.65 mmol, 1.3 eq.). The resulting mixture was stirred at 70 °C overnight. Then, the mixture was evaporated and re-dissolved in DCM and pre-adsorbed onto Celite®. Then, purification by column chromatography (0 → 10% EA/PET) gave **25** (0.089 g, 0.26 mmol) as an oil in 51% yield. ^1^H-NMR (300 MHz, CDCl_3_) δ: 1.36 (s, 9H, *t*Bu), 1.40 (s, 9H, *t*Bu), 3.07–3.16 (m, 2H, CH_2_), 4.39–4.47 (m, 1H, CHNH), 5.00 (d, *J* = 7.8 Hz, 1H, NH), 5.34 (dd, *J* = 11.1, 1.5 Hz, H_vinyl_), 5.67 (d, *J* = 17.4 Hz, 1H, H_vinyl_), 7.04 (dd, *J* = 17.4, 11.1 Hz, 1H, H_vinyl_), 7.11–7.25 (m, 3H, H_Phe_), 7.50 (dd, *J* = 7.8, 1.8 Hz, 1H, H_Phe_). ^13^C-NMR (75 MHz, CDCl_3_) δ: 28.0 (*t*Bu, CH_3_), 28.5 (*t*Bu, CH_3_), 36.3 (CH_2_), 54.9 (CHNH), 79.8 (*t*Bu, C-(CH_3_)_3_), 82.1 (*t*Bu, C-(CH_3_)_3_), 116.3 (vinyl-CH_2_), 126.1 (vinyl-CH), 127.34, 127.7, 130.8, 134.2, 134.6, 137.6, 155.1 (C=O_Boc_), 171.3 (C=O_ester_). HRMS (ESI): calculated for C_20_H_30_NO_4_ ([M + H]^+^): 348.2169, found: 348.2176. [α]^20^_D_ = +10.71 (*c* 0.56, CHCl_3_).

#### *tert*-butyl (R)-2-((*tert*-butoxycarbonyl)amino)-3-(2-vinylphenyl)propanoate (26 – d-type)

According to the procedure described for **25**; **24** (0.605 g, 1.5 mmol) was transformed into **26** (0.38 g, 1.0 mmol) as a yellowish oil in 67% yield. ^1^H-NMR (300 MHz, CDCl_3_) δ: 1.35 (s, 9H, *t*Bu), 1.40 (s, 9H, *t*Bu), 3.07–3.16 (m, 2H, CH_2_), 4.43 (q, *J* = 7.2 Hz, 1H, CHNH), 5.00 (d, *J* = 7.5 Hz, 1H, NH), 5.34 (dd, *J* = 11.1, 1.5 Hz, 1H, vinyl-H), 5.67 (dd, *J* = 17.4, 1.2 Hz, 1H, H_vinyl_), 7.04 (dd, *J* = 17.4, 11.1 Hz, 1H, H_vinyl_), 7.11–7.26 (m, 3H, H_Phe_), 7.50 (dd, *J* = 7.2, 1.8 Hz, 1H, H_Phe_). ^13^C-NMR (75 MHz, CDCl_3_) δ: 28.0 (*t*Bu, CH_3_), 28.4 (*t*Bu, CH_3_), 36.3 (CH_2_), 54.9 (CHNH), 79.8 (*t*Bu-C-(CH_3_)_3_), 82.1 (*t*Bu-C-(CH_3_)_3_), 116.3 (vinyl-CH_2_), 126.0 (vinyl-CH), 127.34, 127.7, 130.8, 134.2, 134.5, 137.6, 155.1 (C=O_Boc_), 171.3 (C=O_ester_). HRMS (ESI): calculated for C_20_H_30_NO_4_ ([M + H]^+^): 348.2169, found: 348.2170. [α]^20^_D_ = −10.21 (*c* 0.48, CHCl_3_).

#### *tert*-butyl (S)-2-((*tert*-butoxycarbonyl)amino)-3-(2-(2-hydroxyethyl)phenyl)propanoate (27 – l-type)

**25** (0.33 g, 0.96 mmol, 1 eq.) was dissolved in anhydrous THF (10 mL, 10 mL/mmol SM) under argon. The resulting solution was cooled to 0 °C in an ice-bath. After 5 min, BH_3_ in THF (1 M in THF; 0.96 mL, 0.96 mmol, 1 eq.) was added. Then, the ice-bath was removed, and the resulting solution stirred at ambient temperature for 1 h. Then, NaBO_3_.H_2_O (0.48 g, 4.8 mmol, 5 eq.) was added, followed by water (10 mL, 10 mL/mmol SM). The mixture was vigorously stirred for 2 h. Next, sat. aq. NH_4_Cl was added together with EA. The layers were separated, and the water layer extracted twice more with EA. The organic layers were combined, dried over Na_2_SO_4_, filtered and evaporated till dryness. The residue was purified by column chromatography (0 → 30% EA/PET) to give **27** (0.23 g, 0.64) as a colorless oil in 66% yield. ^1^H-NMR (300 MHz, CDCl_3_) δ: 1.35 (s, 9H, *t*Bu), 1.38 (s, 9H, *t*Bu), 2.28 (br. s, 1H, OH), 2.92–3.16 (m, 4H, Ph-CH_2_), 3.89 (t, *J* = 6.3 Hz, 2H, CH_2_OH), 4.49 (q, *J* = 7.2 Hz, 1H, CHNH), 5.18 (d, *J* = 7.8 Hz, 1H, NH), 7.13–7.22 (m, 4H, H_Phe_). ^13^C-NMR (75 MHz, CDCl_3_) δ: 28.0 (*t*Bu, CH_3_), 28.4 (*t*Bu, CH_3_), 35.8 (CH_2_), 36.8 (CH_2_), 54.8 (CHNH), 63.6 (CH_2_OH), 80.0 (*t*Bu-C-(CH_3_)_3_), 82.2 (*t*Bu-C-(CH_3_)_3_), 126.4, 127.3, 130.0, 131.0, 135.4, 137.6, 155.2 (C=O_Boc_), 171.4 (C=O_ester_). HRMS (ESI): calculated for C_20_H_32_NO_5_ ([M + H]^+^): 366.2275, found: 366.2278. [α]^20^_D_ = +7.17 (*c* 0.60, CHCl_3_).

#### *tert*-butyl (R)-2-((*tert*-butoxycarbonyl)amino)-3-(2-(2-hydroxyethyl)phenyl)propanoate (28 – d-type)

According to the procedure as described for **27**; **26** (0.34 g, 0.99 mmol) was transformed into **28** (0.22 g, 0.60 mmol) as a colorless oil in 61% yield. ^1^H-NMR (300 MHz, CDCl_3_) δ: 1.35 (s, 9H, *t*Bu), 1.38 (s, 9H, *t*Bu), 2.33 (br. s, 1H, OH), 2.95 (dd, *J* = 13.8, 6.6 Hz, 1H, PhCH_2_), 2.95 (dd, *J* = 13.8, 8.1 Hz, 2H, PhCH_2_), 3.13 (dd, *J* = 13.8, 6.9 Hz, 1H, PhCH_2_), 3.90 (t, *J* = 6.3 Hz, 2H, CH_2_OH), 4.45–4.53 (m, 1H, CHNH), 5.17 (d, *J* = 8.1 Hz, 1H, NH), 7.12–7.23 (m, 4H, H_Phe_). ^13^C-NMR (75 MHz, CDCl_3_) δ: 28.0 (*t*Bu, CH_3_), 28.4 (*t*Bu, CH_3_), 35.8 (CH_2_), 37.0 (CH_2_), 54.8 (CHNH), 63.6 (CH_2_OH), 80.0 (*t*Bu-C-(CH_3_)_3_), 82.3 (*t*Bu-C-(CH_3_)_3_), 126.5, 127.3, 130.0, 131.1, 135.5, 137.6, 155.2 (C=O_Boc_), 171.4 (C=O_ester_). HRMS (ESI): calculated for C_20_H_32_NO_5_ ([M + H]^+^): 366.2275, found: 366.2260. [α]^20^_D_ = -6.67 (*c* 0.48, CHCl_3_).

#### *tert*-butyl (S)-2-((*tert*-butoxycarbonyl)amino)-3-(2-(2-fluoroethyl)phenyl)propanoate (29 – l-type)

**27** (0.090 g, 0.25 mmol, 1 eq.) was dissolved in anhydrous DCM (2.5 mL, 10 mL/mmol SM) under argon and cooled in an ice bath at 0 °C. Then, DAST (0.10 mL, 0.74 mmol, 3 eq.) was added dropwise and the mixture was stirred at ambient temperature for approximately 3–4 h. Then, the mixture was cooled to 0 °C in an ice bath and aq. sat. NaHCO_3_ solution was added dropwise. After the gas evolution ceased, the mixture was transferred into a separatory funnel and more sat. aq. NaHCO_3_ solution was added. The layers were separated, and the water layer extracted twice more with DCM. The organic layers were combined, dried over Na_2_SO_4_, filtered and evaporated till dryness. The residue was purified by column chromatography (0 → 12% EA/PET) to give **29** (0.041 g, 0.11 mmol) as a yellowish oil in 45% yield. ^1^H-NMR (300 MHz, CDCl_3_) δ: 1.37 (s, 9H, *t*Bu), 1.40 (s, 9H, *t*Bu), 3.01–3.17 (m, 4H, PhCH_2_), 4.37–4.45 (m, 1H, PhCHNH), 4.63 (dt, *J* = 46.8, 6.9 Hz, 2H, CH_2_F), 5.03 (d, *J* = 8.4 Hz, 1H, NH), 7.11–7.22 (m, 4H, H_Phe_). ^19^F-NMR (282 MHz, CDCl_3_) δ: −215.01 – −214.52 (m). ^13^C-NMR (75 MHz, CDCl_3_) δ: 28.0 (*t*Bu, CH_3_), 28.4 (*t*Bu, CH_3_), 33.6 (d, *J* = 20.6 Hz, 1C, PhCH_2_CH_2_F), 36.1 (PhCH_2_), 54.9 (CHNH), 79.9 (*t*Bu-C-(CH_3_)_3_), 82.2 (*t*Bu-C-(CH_3_)_3_), 84.0 (d, *J* = 169.4 Hz, 1C, PhCH_2_CH_2_F), 126.9, 127.3, 130.1, 130.8, 135.3 (Ph-C-CH_2_CHNH), 135.9 (d, *J* = 6.9 Hz, 1C, Ph-C-CH_2_CH_2_F), 155.2 (C=O_Boc_), 171.4 (C=O_ester_). HRMS (ESI): calculated for C_20_H_31_FNO_4_ ([M + H]^+^): 368.2232, found: 368.2220. [α]^20^_D_ = +21.87 (*c* 0.48, CHCl_3_).

#### *tert*-butyl (R)-2-((*tert*-butoxycarbonyl)amino)-3-(2-(2-fluoroethyl)phenyl)propanoate (30 – d-type)

According to the procedure as described for **29**; **28** (0.185 g, 0.506 mmol) was transformed into **30** (0.101 g, 0.273 mmol) as a colorless oil in 54% yield. ^1^H-NMR (300 MHz, CDCl_3_) δ: 1.36 (s, 9H, *t*Bu), 1.39 (s, 9H, *t*Bu), 2.97–3.19 (m, 4H, Ph-CH_2_), 4.37–4.44 (m, 1H, PhCHNH), 4.62 (dt, *J* = 47.1, 6.9 Hz, 2H, CH_2_F), 5.05 (d, *J* = 8.4 Hz, 1H, NH), 7.15–7.23 (m, 4H, H_Phe_). ^19^F-NMR (282 MHz, CDCl_3_) δ: −215.02 – −214.53 (m). ^13^C-NMR (75 MHz, CDCl_3_) δ: 28.0 (*t*Bu, CH_3_), 28.4 (*t*Bu, CH_3_), 33.6 (d, *J* = 20.6 Hz, 1C, PhCH_2_CH_2_F), 36.1 (PhCH_2_), 54.9 (CHNH), 79.8 (*t*Bu-C-(CH_3_)_3_), 82.2 (*t*Bu -C-(CH_3_)_3_), 83.9 (d, *J* = 168.3 Hz, 1C, PhCH_2_CH_2_F), 126.9, 127.3, 130.0, 130.7, 135.3 (Ph-C-CH_2_CHNH), 135.9 (d, *J* = 5.7 Hz, 1C, Ph-C-CH_2_CH_2_F), 155.2 (C=O_Boc_), 171.3 (C=O_ester_). HRMS (ESI): calculated for C_20_H_32_NO_5_ ([M + H]^+^): 366.2275, found: 366.2260. [α]^20^_D_ = −22.40 (*c* 0.50, CHCl_3_).

#### *tert*-butyl (S)-2-((*tert*-butoxycarbonyl)amino)-3-(2-(2-(tosyloxy)ethyl)phenyl)propanoate (31 – l-type)

**27** (0.22 g, 0.60 mmol, 1 eq.) was dissolved in anhydrous DCM (6.0 mL, 10 mL/mmol SM), after which Et_3_N (0.252 mL, 1.81 mmol, 3 eq.) was added. Then, a catalytic amount of DMAP followed by tosylchloride (0.138 g, 0.722 mmol, 1.2 eq.) were added, and the resulting solution stirred at ambient temperature for 2 h. Next, water was added, and the layers separated. The water layer was extracted twice with DCM. The organic layers were combined, dried over Na_2_SO_4_, filtered and evaporated. The residue was purified by column chromatography (0 → 20% EA/PET) to give **31** (0.175 g, 0.337 mmol) as a colorless oil in 56% yield. ^1^H-NMR (300 MHz, CDCl_3_) δ: 1.35 (s, 9H, *t*Bu), 1.39 (s, 9H, *t*Bu), 2.43 (s, 3H, CH_3_), 2.87–3.12 (m, 4H, PhCH_2_), 4.12–4.24 (m, 2H, CH_2_OTs), 4.30–4.37 (m, 1H, CHNH), 4.99 (d, *J* = 8.4 Hz, 1H, NH), 7.05–7.16 (m, 4H, H_Phe_), 7.26–7.30 (m, 2H, H_Phe-Ts_), 7.68–7.72 (m, 2H, H_Phe-Ts_). ^13^C-NMR (75 MHz, CDCl_3_) δ: 21.8 (CH_3_), 28.0 (*t*Bu, CH_3_), 28.4 (*t*Bu, CH_3_), 32.1 (PhCH_2_CH_2_OTs), 36.0 (PhCH_2_CH), 54.8 (PhCH_2_CH), 70.3 (PhCH_2_CH_2_OTs), 79.9 (*t*Bu-C-(CH_3_)_3_), 82.3 (*t*Bu-C-(CH_3_)_3_), 127.1, 127.4, 128.0, 129.95, 130.04, 130.8, 133.2, 135.0, 135.3, 144.8, 155.1 (C=O_Boc_), 171.21 (C=O_ester_). HRMS (ESI): calculated for C_27_H_28_NO_7_S ([M + H]^+^): 520.2363, found: 520.2375. [α]^20^_D_ = +11.06 (*c* 1.09, CHCl_3_).

#### (S)-2-amino-3-(2-(2-fluoroethyl)phenyl)propanoic acid (32 – l-type, 2-FELP)

**29** (0.090 g, 0.25 mmol) was dissolved in DCM (3.0 mL) after which TFA (3.0 mL) was added. The resulting solution was stirred at ambient temperature for approximately 5 hours, after which it was evaporated till dryness. Next, the residue was co-evaporated with MeOH three times. Then, the residue was dissolved in ~2 mL of MeOH and the pH was adjusted to pH 6–7, with diluted aq. NH_3_ solution, after which a white precipitate formed. The mixture was refrigerated overnight, and then the liquid was removed via pipette aspiration. The remaining solid was washed thrice with a minimal amount of ice-cold MeOH and dried under high vacuum to give **2-FELP** (0.012 g, 0.057 mmol) as a white powder in 23% yield. ^1^H-NMR (300 MHz, D_2_O) δ: 3.10 (dd, *J* = 14.7, 9.0 Hz, 1H, PhCH_2_CH), 3.15 (dt, *J* = 26.4, 6.0 Hz, 2H, PhCH_2_CH_2_F), 3.43 (dd, *J* = 14.7, 6.0 Hz, 1H, PhCH_2_CH), 3.95 (dd, *J* = 9.0, 6.0 Hz, 1H, PhCH_2_CH), 4.77 (dt, *J* = 47.4, 6.0 Hz, 2H, PhCH_2_CH_2_F), 7.31–7.43 (m, 4H, H_Phe_). ^19^F-NMR (282 MHz, D_2_O) δ: −218.58 (tt, *J* = 46.8, 26.5 Hz, 1 F). ^13^C-NMR (75 MHz, D_2_O) δ: 32.4 (d, *J* = 19.4 Hz, 1C, PhCH_2_CH_2_F), 33.5 (PhCH_2_CH), 55.6 (PhCH_2_CH), 85.0 (d, *J* = 161.4 Hz, 1C, PhCH_2_CH_2_F), 127.3, 128.0, 130.3, 130.4, 134.0, 136.5 (d, *J* = 4.6 Hz, 1C, Ph-C-CH_2_CH_2_F), 173.9 (C=O). HRMS (ESI): calculated for C_11_H_15_FNO_2_ ([M + H]^+^): 212.1081, found: 212.1078.

Chiral HPLC: Astec® Chirobiotic T column (5 µm, 125 mm × 4.6), EtOH(abs.)/H_2_O (80/20), retention time: 4.01 min, ee: 90.4%.

#### (R)-2-amino-3-(2-(2-fluoroethyl)phenyl)propanoic acid.HCl salt (33 – d-type, 2-FEDP)

**30** (0.090 g, 0.25 mmol) was dissolved in DCM (3.0 mL) after which TFA (3.0 mL) was added. The resulting solution was stirred at ambient temperature for approximately 5 hours, after which it was evaporated till dryness. Next, the residue was co-evaporated with MeOH three times. Then, the residue was dissolved in ~2 mL of MeOH and the pH was adjusted to pH 6–7, with diluted aq. NH_3_ solution, after which a white precipitate formed. The mixture was refrigerated overnight, and then the liquid was removed via pipette aspiration. The remaining solid was washed once with a minimal amount of ice-cold MeOH and re-dissolved in MeOH. Then, 1.25 M HCl in MeOH was added and the mixture evaporated till dryness. The residue was dissolved in water and lyophilized, which gave rise to **34** (0.030 g, 0.12 mmol) as a white, amorphous powder in 49% yield. ^1^H-NMR (300 MHz, D_2_O) δ: 3.14 (dt, *J* = 26.7, 6.0 Hz, 2H, PhCH_2_CH_2_F), 3.13–3.21 (m, 1H, PhCH_2_CH), 3.48 (dd, *J* = 14.7, 6.3 Hz, 1H, PhCH_2_CH), 4.13–4.19 (m, 1H, PhCH_2_CH), 4.77 (dt, *J* = 47.1, 6.0 Hz, 2H, PhCH_2_CH_2_F), 7.31–7.44 (m, 4H, H_Phe_). ^19^F-NMR (282 MHz, D_2_O) δ: −216.10 (tt, *J* = 46.8, 26.5 Hz, 1 F). ^13^C-NMR (75 MHz, D_2_O) δ: 32.35 (d, *J* = 19.4 Hz, 1C, PhCH_2_CH_2_F), 33.1 (PhCH_2_CH), 54.4 (PhCH_2_CH), 85.0 (d, *J* = 161.4 Hz, 1C, PhCH_2_CH_2_F), 127.4, 128.2, 130.3, 130.4, 133.4, 136.6 (d, *J* = 4.6 Hz, 1C, Ph-C-CH_2_CH_2_F), 172.4 (C=O). HRMS (ESI): calculated for C_11_H_15_FNO_2_ ([M + H]^+^): 212.1081, found: 212.1085.

Chiral HPLC Astec® Chirobiotic T column (5 µm, 125 mm × 4.6), EtOH(abs.)/H_2_O (80/20), retention time: 5.21 min, *ee*: 75.2%.

### *In vitro* experiments

#### General information

The F98 GB cell line was obtained from ATCC and cultivated as described previously^11^. Cells were tested and authenticated by the provider and were cultured for maximum ten weeks after retrieval from liquid nitrogen.

LAT1 expression was determined using flow cytometry. F98 cells (100,000 per well) were surface stained with a monoclonal antibody against LAT1 (PE-conjugated, Santa Cruz biotechnology, Heidelberg, Germany), which acts as a negative control. As described by De Munter *et al*.^50^, cells were subsequently fixed, permeabilized and submitted for intracellular staining with the same PE conjugated monoclonal antibody against LAT1. Flow cytometric analysis was performed using the LSR II (BD Biosciences).

*In vitro* experiments were carried out in 24-well-plates (VWR, US), using at least three wells for each data-point. The cells were seeded 24 h prior to the experiment at 200,000 cells/well. Influx of radiolabeled AA was studied in a Na^+^ containing buffer (HEPES+ buffer: pH 7.4; 100 mM NaCl (Sigma Aldrich, Belgium), 2 mM KCl (Sigma Aldrich, Belgium), 1 mM MgCl_2_ (VWR, US), 1 mM CaCl_2_ (VWR, US), 10 mM Hepes (Sigma Aldrich, Belgium), 5 mM Tris (VWR, US), 1 g/L glucose (VWR, US) and 1 g/L Bovine Serum Albumin (Sigma Aldrich, Belgium)) and/or a Na^+^ free buffer (HEPES- buffer: pH 7.4; 100 mM Choline-Cl (Sigma Aldrich, Belgium), 2 mM KCl, 1 mM MgCl_2_, 1 mM CaCl_2_, 10 mM Hepes, 5 mM Tris, 1 g/L glucose and 1 g/L Bovine Serum Albumin). The culture medium was removed, and cells were washed twice with 1 mL HEPES+/HEPES- buffer. Dosing solutions were prepared by supplementing the washing buffer with either 11 kBq [2, 3, 4, 5, 6-^3^H]-l-phenylalanine (Perkin Elmer, Massachusetts, USA)/mL or with 37 kBq of the [^18^F]-labelled AA. The incubation process was terminated by cooling the plates on ice and adding 1 mL 1% BSA ice-cold PBS. Cells were washed twice with 2 mL ice-cold PBS. Subsequently, cells were lysed with 250 µL 0.1 M NaOH (VWR, US) and radioactivity stemming from the [^18^F]-isotope was counted by subjecting 150 µL of this solution to an automated gamma-counter (Cobra-inspector 5003, Canberra Packard, Meriden, CT, USA). [^3^H] samples (150 µL) were transferred to a scintillation bottle (Perkin Elmer, Massachusetts, USA) containing 5 mL of scintillation liquid (Ultima Gold, Perking Elmer, Massachusetts, USA) and counted using an automated scintillation counter (TriCarb 2900 TR; Perkin Elmer, Massachusetts, USA). Of each well, 25 µL was subjected to a BCA assay (ThermoFisher Scientific) to determine protein content.

#### *Concentration dependency using* [2, 3, 4, 5, 6-^3^H]-l-phenylalanine

Non-linear curve fitting was performed to determine the Michaelis-Menten kinetics using Graphpad Prism v5.01 (Graphpad software, San Diego, CA, USA). These were employed to study the potential inhibition of the AA analogue towards the [^3^H]-l-Phe/l-Phe couple with varying concentrations (0.01 to 0.5 mM) at 1 min uptake (V_0_ conditions). The apparent K_m_ and corresponding K_i_ values were calculated according to the formula:

$$Ki=[I]/((K{m}_{app}/Km)-1)$$

where [I] is the inhibitor concentration (i.e. the fluorinated AA), K_m_ is the Michaelis-Menten constant of the substrate (i.e. ³H-l-Phe/l-Phe) and K_m,app_ is the apparent value of K_m_ for substrate transport in the presence of the inhibitor.

### Radiochemical synthesis and purification

The radiofluorinated AA analogues, [^18^F]FET and 2-(2-[^18^F]fluoroethyl)-l-phenylalanine (2-[^18^F]FELP) were prepared on a Synthra RN plus module (Synthra GmbH, Hamburg, Germany) with an integrated semi-preparative HPLC, using identical reaction and purification conditions for both tracers, based on the method described by Bourdier *et al*.^51^ (depicted in Supplementary Fig. 1). The precursor solution for radiolabeling was prepared by dissolving 6 mg tert-butyl (S)-3-(4-(2-(tosyloxy)ethoxy)phenyl)-2-(tritylamino)propanoate (ABX, Germany) or 9 mg tert-butyl (S)-2-((tert-butoxycarbonyl)amino)-3-(2-(2-(tosyloxy)ethyl)phenyl)propanoate in 1 mL acetonitrile (Sigma Aldrich, Belgium). The precursor solution was added to a dried [^18^F]F^−^/Kryptofix®222/K^+^ complex, heated at 100 °C for 15 min. Subsequently, deprotection was performed by adding 2 M HCl (1 mL) for 15 min. The solution was then neutralized with 4 M NaOH, followed by adding 0.10 M NH_4_OAc. HPLC purification was performed using a RP symmetry Prep C18 column (5 µm, 10 × 250 mm, Machery-Nagel, Duren, Germany), a 0.01 M NH_4_OAc buffer in ethanol/water 5/95 (V/V) mobile phase, and a flow rate of 5 mL/min. The fraction containing purified [^18^F]FET or 2-[^18^F]FELP eluted at approximately 12 min and 15 min respectively (depicted in Supplementary Figs 2 and 3 for [^18^F]FELP), and was collected for 2 minutes. This procedure yielded in 3.32 ± 1.16 GBq [^18^F]FET or 3.01 ± 0.97 MBq 2-[^18^F]FELP. Quality control of the final formulation was performed by radio-TLC using Polygram TLC stripes (Macherey-Nagel, Germany), a mobile phase consisting of ACN/H_2_O 95/5 (V/V), and miniGITA (Raytest, Germany) analysis software. Radiochemical purities of ≥95% were obtained for [^18^F]FET and for 2-[^18^F]FELP. Chiral purity of [^18^F]FELP was assessed as described for the unlabelled amino acid.

1 MBq of 2-[^18^F]FELP or 2-[^18^F]FET was added to a 1:1 v:v mixture of MilliQ water and n-octanol in a test tube. After vortexing and centrifugation (10 min; 1100 g) of the samples, aliquots were taken from each layer and counted separately in a NaI (Tl) scintillation counter (Capintec; Ramsey, NJ, USA). The partition coefficient was calculated using the ratio of activity detected in n-octanol and in the aqueous layer to obtain the logP_oct_ value.

#### *Concentration and time dependency using* 2-[^18^F]FELP or [^18^F]FET

Concentration dependency was measured at V_0_ conditions with concentrations of the AA varying from 0.01 to 0.5 mM. The data were fitted to the Michaelis-Menten model and the K_m_ was calculated.

The cells were incubated for times ranging from 1–60 min in 250 µL of HEPES+ buffer supplemented with 37 kBq of 2-[^18^F]FELP or [^18^F]FET.

To determine the selectivity of the LAT1 transporter for the fluor-18 labelled AA, the *in vitro* uptake was studied in the presence of Na^+^-rich or Na^+^-free buffer(HEPES+/− buffer), 8 mM of the system L and b^0,+^ inhibitor 2-aminobicycloheptane-2-carboxylic acid (BCH) (Sigma Aldrich, Belgium), 8 mM of the system A inhibitor methylaminoisobutyric acid (MeAiB) (Sigma Aldrich, Belgium) or 5 mM of the LAT1 specific inhibitor JPH-203 (ARK Pharm, USA). The uptake studies supplemented with the transporter specific inhibitors were carried out in HEPES+ buffer.

#### *In vivo* experiments

The study was performed in accordance with the appropriate guidelines and regulations and approved by the Ghent University Ethical Committee on animal experiments (ECD15/39). The GB F98 rat model was developed as described by Bolcaen *et al*.^11^. Six female NIH-Foxn1^rnu^ rats were inoculated with ±20,000 F98 tumor cells in the right frontal lobe. The rats were anesthetized with ketamine/xylazine (4/3; 0.13 ml/100 g) and were kept separately post-inoculation (p.i.).

Tumor growth was evaluated 8 days p.i. using MRI (7T PharmaScan 70/16, Bruker BioSpin, Ettlingen, Germany). The rats were anesthetized with 2% isoflurane mixed with oxygen administered at a flow rate of 0.3 L/min. After fixation, a heated blanket was placed on the animal. A rat brain surface coil (Rapid Biomedical, Rimpar, Germany) was placed around the head, after which the holder was positioned in a 72 mm rat whole body transmitter coil (Rapid Biomedical, Rimpar, Germany). After performing a localizer scan, a T2-weighted spin-echo scan (TR/TE 3661/37.1 ms, 109 μm isotropic in plane resolution, 4 averages, TA 9′45″) was obtained. If a tumor was visible, a gadolinium-containing contrast agent (Dotarem, Guerbet, France; 0.4 mL/kg) was injected intravenously through a 30-Gauge needle connected to a 60 cm long PE tube into the tail vein. A contrast-enhanced T1-weighted spin-echo sequence (TR/TE 1539/9.7 ms, 117 µm isotropic in plane resolution, 3 averages, TA 4′15″) was performed 15 min post injection of the contrast agent.

When the presence of a tumor was confirmed, additional contrast-enhanced T1-weighted spin-echo scans were obtained, as above-mentioned. Post-MRI dynamic PET scans were acquired using 2-[^18^F]FELP, [^18^F]FET and [^18^F]FDG on three consecutive days (day 13, 14 and 15 p.i.). The total acquisition time was 120 min for both dynamic AA PET scans and 60 min for the dynamic [^18^F]FDG PET ([^18^F]FDG_early_). In addition, a static 30 min [^18^F]FDG PET was acquired 240 min post-injection ([^18^F]FDG_late_).

All PET scans were reconstructed into a 200 × 200 × 128 matrix by a 3D Maximum Likelihood Expectation Maximization (MLEM) algorithm (LabPET Version 1.12.1, TriFoil Imaging®, Northridge CA) using 50 iterations and a voxel size of 0.5 × 0.5 × 0.59675 mm. The dynamically acquired PET data were reconstructed into different time frames. Time frames were 6 × 20 s, 3 × 1 min, 3 × 5 min and 2 × 20 min for [^18^F]-FDG scans and 6 × 20 s, 3 × 1 min, 3 × 5 min and 5 × 20 min for the AA PET.

Rigid body PET-MRI co-registration was done using PMOD (PMOD technologies®, Zürich, Switzerland) and volumes of interest (VOI) were manually drawn, including the contrast-enhanced region on T1-weighted MRI. Cubic VOIs of 2 × 2 × 2 mm located in the contralateral region were used as a reference. Tracer uptake in the VOI at each time frame was converted to a standard uptake value (SUV) according to the following formula:

$${\rm{SUV}}=(\text{Radioactivity}\,{\rm{in}}\,\text{VOI}/\text{injected}\,\text{activity})\times {\rm{body}}\,{\rm{weight}}$$

Injected activity was corrected for radioactive decay and residual activity in the syringe. Standardized uptake values (SUV_mean_ and SUV_max_) and tumor-to-background ratios (TBR_mean_ and TBR_max_) were calculated.

### Data analysis

All data are expressed as means ± SD. All curves were constructed using GraphPad Prism 5.0 (GraphPad Software, San Diego, CA, USA). Where applicable, statistical analysis was performed using the Mann-Whitney U test. A probability value of *p* < 0.05 was considered statistically significant.

## Supplementary information

Supporting information: Synthesis and evaluation of new fluoroethyl derivatives of phenylalanine targeting LAT1 transporters in F98 glioblastoma cells: towards improved PET tracers.

## Data Availability

All data generated or analyzed during this study are included in this published article (and its supplementary information file).

## Footnotes

1 *This peak is in fact a doublet of triplets (F-coupling); however, the peak coincides with the PhCH2CH signal and was not sufficiently resolved for adequate calculation.
