# Supplementary material for: New fluoroethyl phenylalanine analogues as potential LAT1-targeting PET tracers for glioblastoma
Source: Sci Rep. 2019 Feb 27;9:2878. doi: 10.1038/s41598-019-40013-x (PMC6393465; doi:10.1038/s41598-019-40013-x)
Supplement: Supplementary file 1 — Supporting information: Synthesis and evaluation of new fluoroethyl derivatives of phenylalanine targeting LAT1 transporters in F98 glioblastoma cells: towards improved PET tracers. [file 41598_2019_40013_MOESM1_ESM.pdf]

Supporting information

New fluoroethyl phenylalanine analogues as potential LAT-1 targeting PET tracers for glioblastoma

Jeroen Verhoeven[1], Fabian Hulpia[2], Ken Kersemans[3], Julie Bolcaen[3], Stef De Lombaerde[1], Jan Goeman[4], Benedicte Descamps[5], Giorgio Hallaert[6], Caroline Van den Broecke[7], Karel Deblaere[8], Christian Vanhove[5], Johan Van der Eycken[4], Serge Van Calenbergh[2], Ingeborg Goethals[3] & Filip De Vos[1]

[1]Laboratory of Radiopharmacy, Ghent University, Ghent, Belgium.

[2]Laboratory for Medicinal Chemistry, Ghent University, Ghent, Belgium.

[3]Ghent University Hospital, Department of Nuclear Medicine, Ghent, Belgium.

[4]Laboratory for Organic and Bio-organic synthesis, Ghent University, Ghent, Belgium.

[5]iMinds-IBiTech-MEDISIP Ghent University, Department of Electronics and Information Systems, Ghent, Belgium.

[6]Ghent University Hospital, Department of Neurosurgery, Ghent, Belgium.

[7]Ghent University Hospital, Department of Pathology, Ghent, Belgium.

[8]Ghent University Hospital, Department of Radiology and Medical Imaging, Ghent, Belgium.

***First author and responsible for correspondence:**

Jeroen Verhoeven

Email: Jeroen.Verhoeven@ugent.be

Table of contents

Chiral purity analysis of compound **3** & **4** **S-5**

Optimization of chromatographic conditions mixture of compound **3** & **4** **S-6**

Chiral HPLC analysis of compound **3** **S-7**

Chiral HPLC analysis of compound **4** **S-9**

Chiral purity analysis of compound **9** & **10** **S-12**

Optimization of chromatographic conditions mixture of compound **9** & **10** **S-13**

Chiral HPLC analysis of compound **9** **S-14**

Chiral HPLC analysis of compound **10** **S-17**

Chiral purity analysis of compound **15** & **16** **S-20**

Optimization of capillary electrophoresis (CE) analysis mixture of compound **15** & **16** **S-21**

Chiral CE analysis of compound **15** **S-23**

Chiral CE analysis of compound **16** **S-26**

Chiral purity analysis of compound **17** & **18** **S-29**

Optimization of capillary electrophoresis (CE) analysis mixture of compound **17** & **18** **S-30**

Chiral CE analysis of compound **17** **S-32**

Chiral CE analysis of compound **18** **S-35**

Chiral purity analysis of compound **32** & **33** **S-38**

Optimization of chromatographic conditions mixture of compound **32** & **33** **S-39**

Chiral HPLC analysis of compound **32** **S-40**

Chiral HPLC analysis of compound **33** **S-42**

Radiosynthesis of compound [^{18}F]-**32** (2-[^{18}F]-FELP): Flow scheme of the custom Synthra RN Module **S-44**

Semi-preparative radiogram of [^{18}F]-**32** (2-[^{18}F]-FELP) **S-45**

Semi-preparative UV-chromatogram of [^{18}F]-**32** (2-[^{18}F]-FELP) **S-46**

Chiral purity analysis of radiolabeled **32** (2-[^{18}F]-FELP) **S-47**

Chiral HPLC analysis of radiolabeled **32** (2-[^{18}F]-FELP) **S-48**

Flow cytometry analysis **S-49**

Michaelis-Menten plots **S-50**

Chiral purity analysis of compound **3** & **4**

Chromatographic conditions for **3** & **4**:

Diacel Chiralpak IA, 250 x 4.6 mm, particle size 5 µm. Column temperature was set at 35 °C. The chromatographic run was performed isocratically with a mixture of *n*-Hexane/EtOH(abs.) (97/3) and a run length of 30 at a flow rate of 1 mL/min.

===

Injection Date	: 12/20/2017 5:45:35 PM	Seq. Line :	14
Sample Name	: G	Location :	Vial 7
Acq. Operator	: Jan Goeman	Inj :	1
		Inj Volume :	Inj prog

Sequence File : C:\HPCHEM\2\SEQUENCE\TEMP2.S
Method : C:\HPCHEM\2\METHODS\AB97-3.M
Last changed : 4/11/2016 11:58:26 AM by Jan Goeman
Chirale kolom ....
30' isocratisch n-Hexaan-EtOH 97:3

±

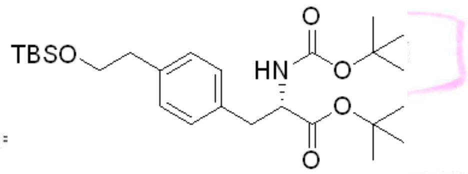

3

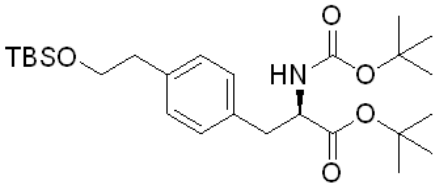

4

===

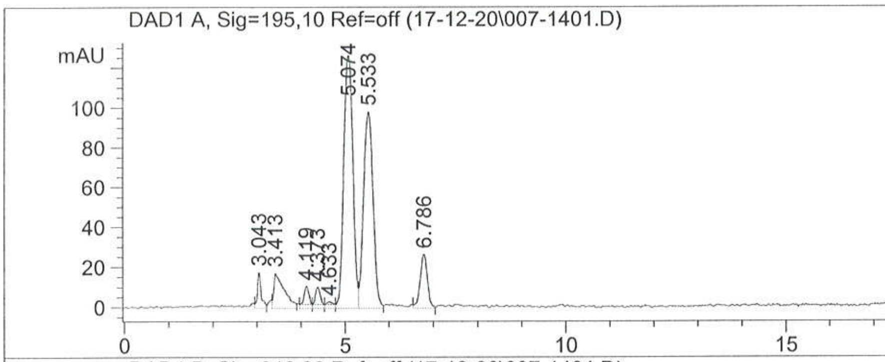

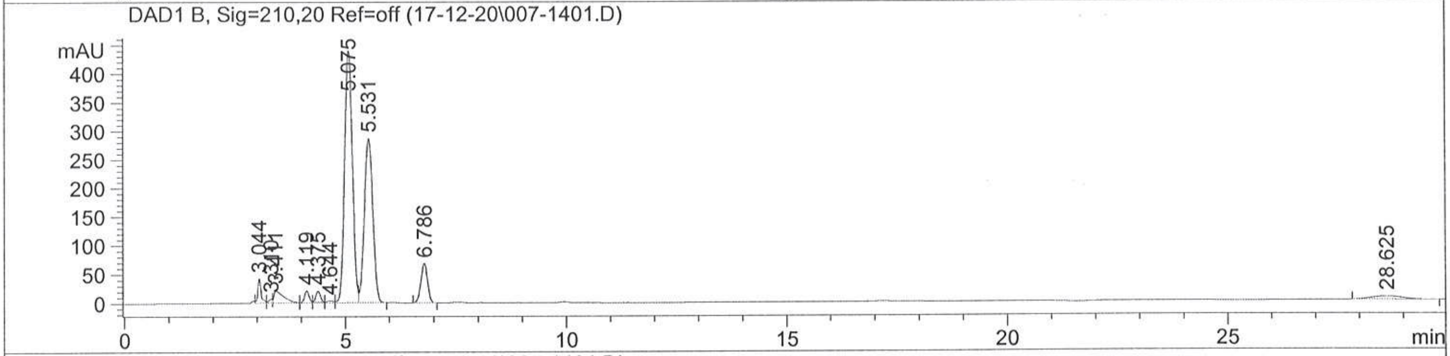

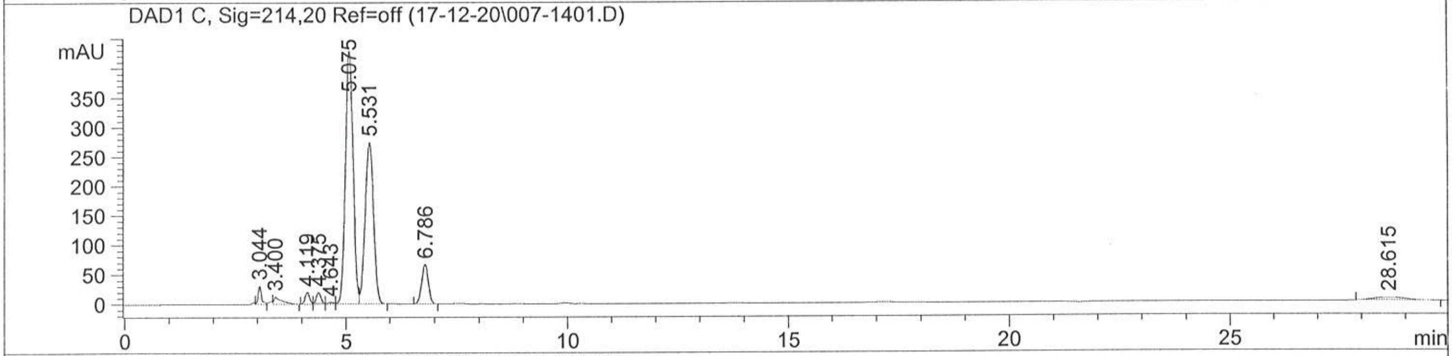

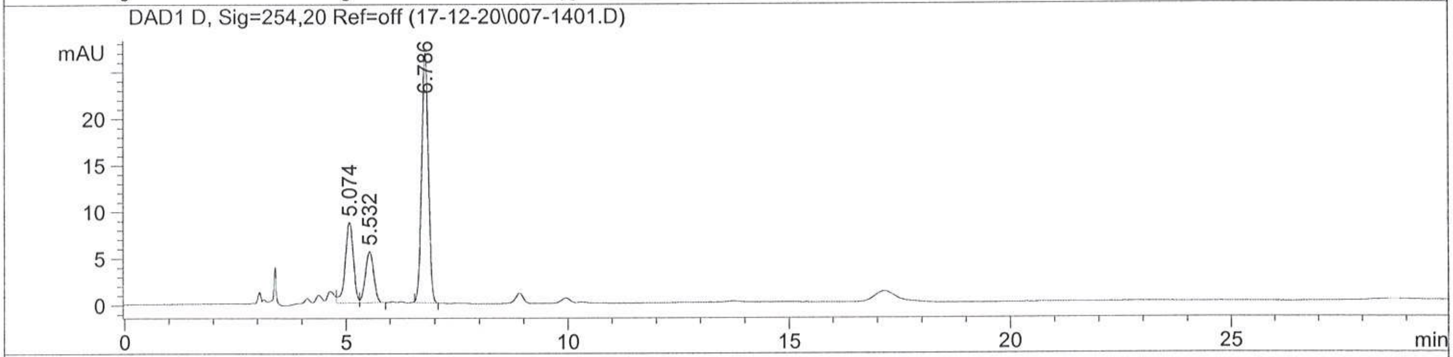

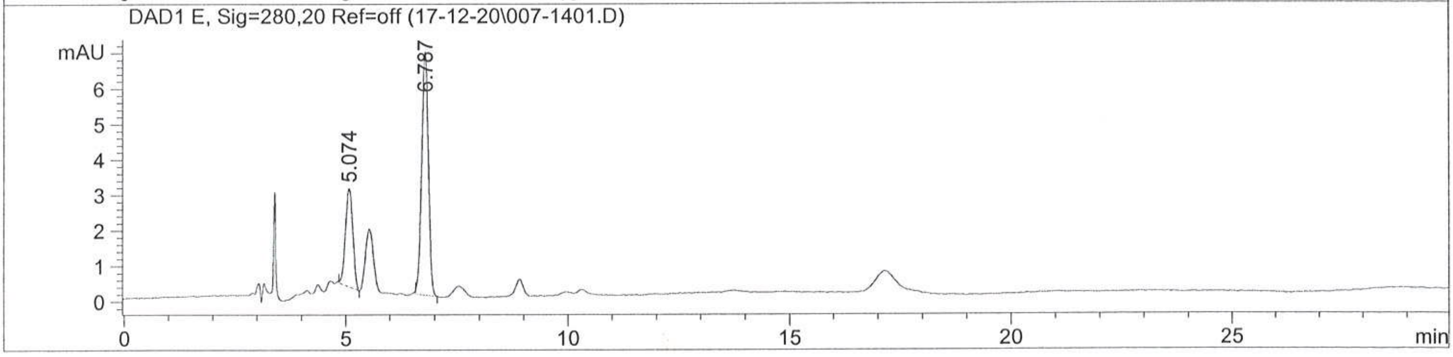

===

Injection Date : 1/30/2018 7:16:41 PM Seq. Line : 2
Sample Name : 13 Location : Vial 75
Acq. Operator : Jan Goeman Inj : 1
Inj Volume : Inj prog

Sequence File : C:\HPCHEM\2\SEQUENCE\TEMP2.S
Method : C:\HPCHEM\2\METHODS\AB97-3.M
Last changed : 4/11/2016 11:58:26 AM by Jan Goeman
Chirale kolom ....
30' isocratisch n-Hexaan-EtOH 97:3

===

Chiral HPLC analysis
Sample: cpd. 3

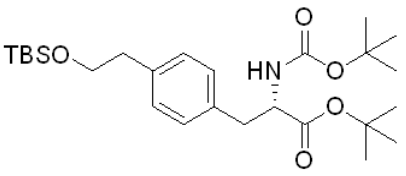

3

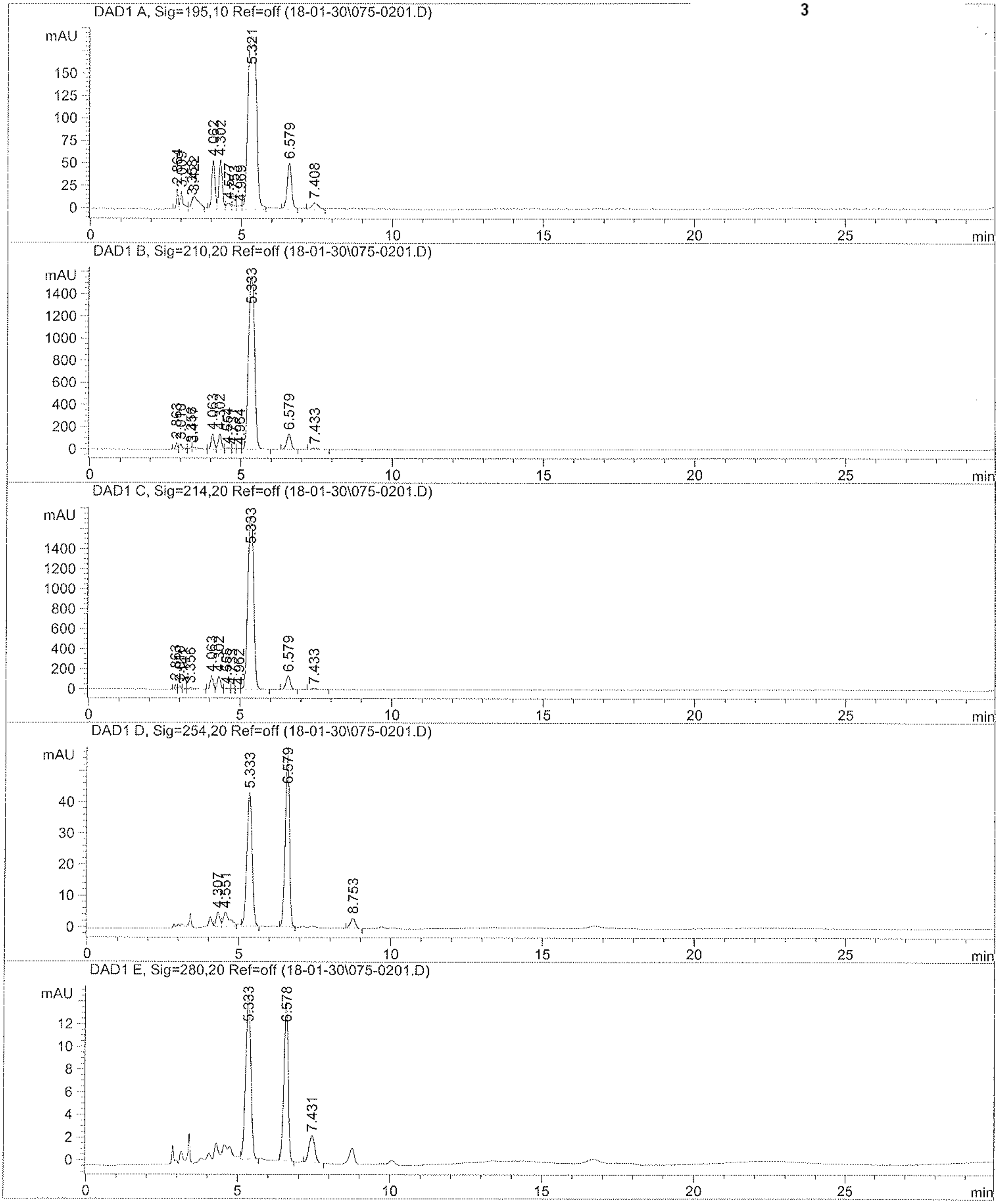

Current Chromatogram(s)

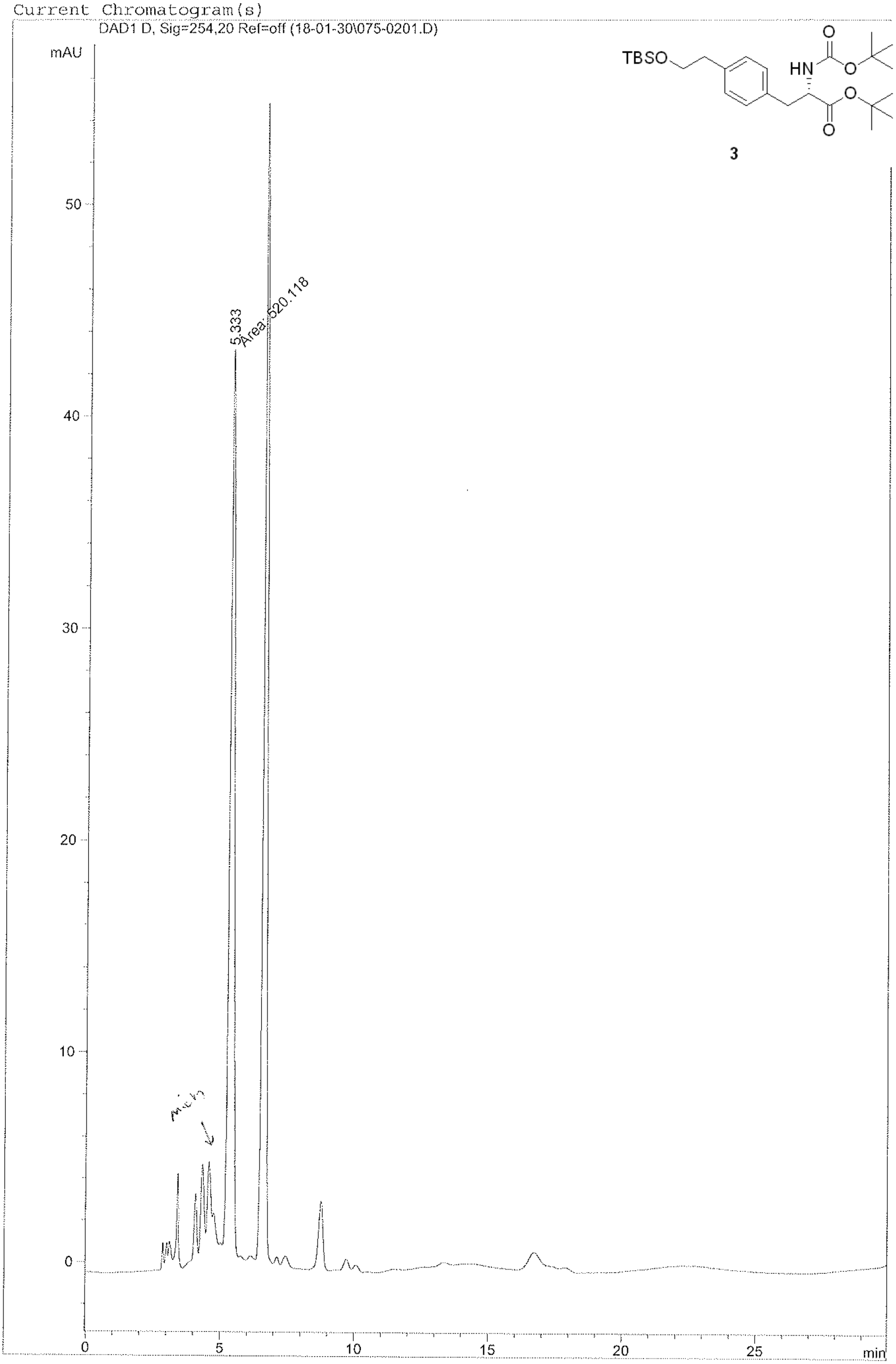

```
=====================================================================
Injection Date  : 1/30/2018 7:51:39 PM          Seq. Line :    3
Sample Name     : 14                             Location : Vial 76
Acq. Operator   : Jan Goeman                          Inj :    1
                                               Inj Volume : Inj prog
Sequence File   : C:\HPCHEM\2\SEQUENCE\TEMP2.S
Method          : C:\HPCHEM\2\METHODS\AB97-3.M
Last changed    : 4/11/2016 11:58:26 AM by Jan Goeman
Chirale kolom ....................
30' isocratisch n-Hexaan-EtOH 97:3
=====================================================================
```

Chiral HPLC analysis:
Sample: cpd. 4

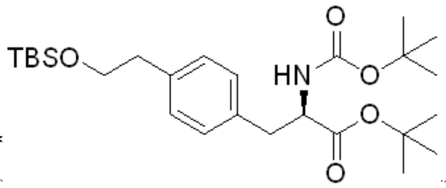

4

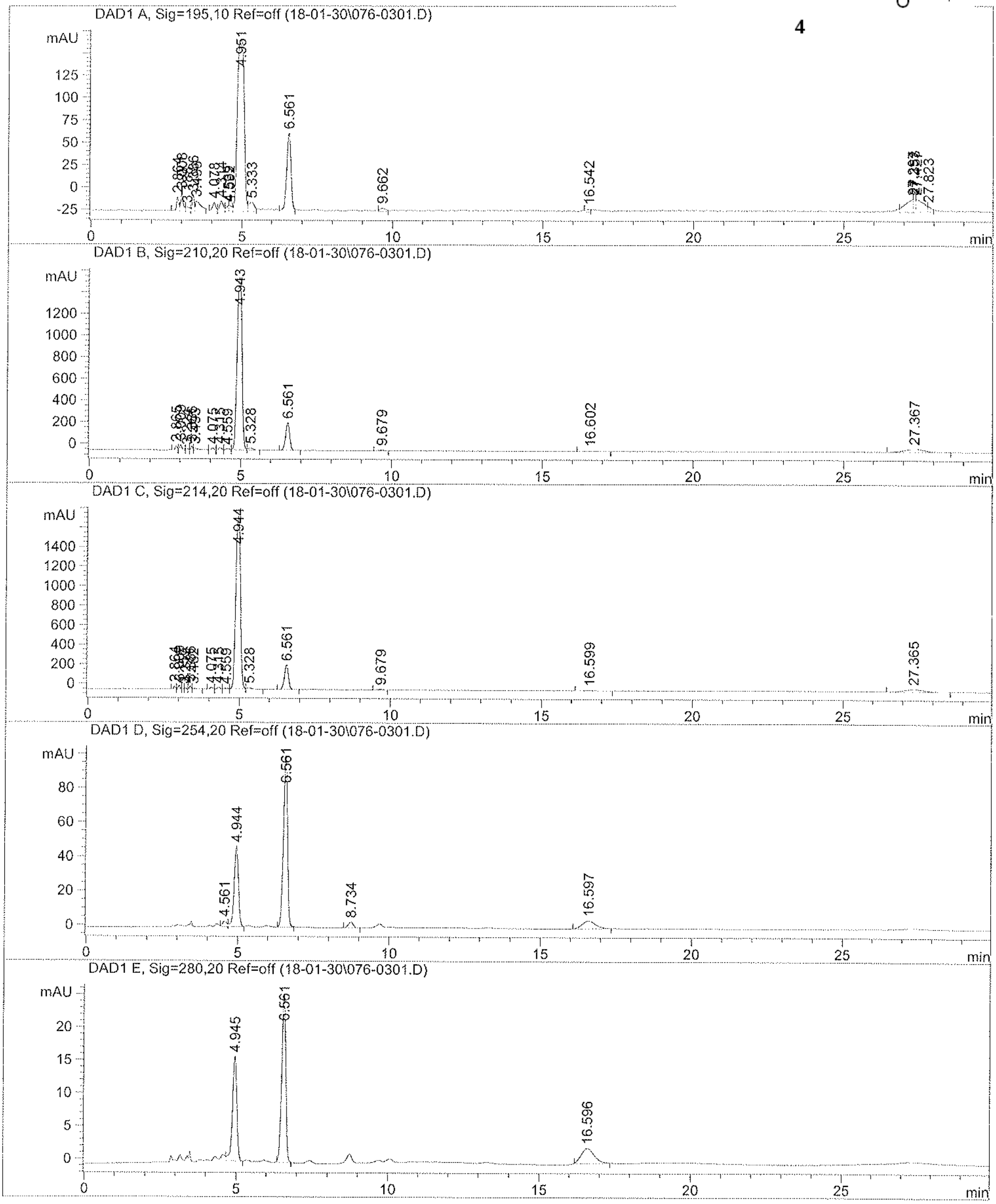

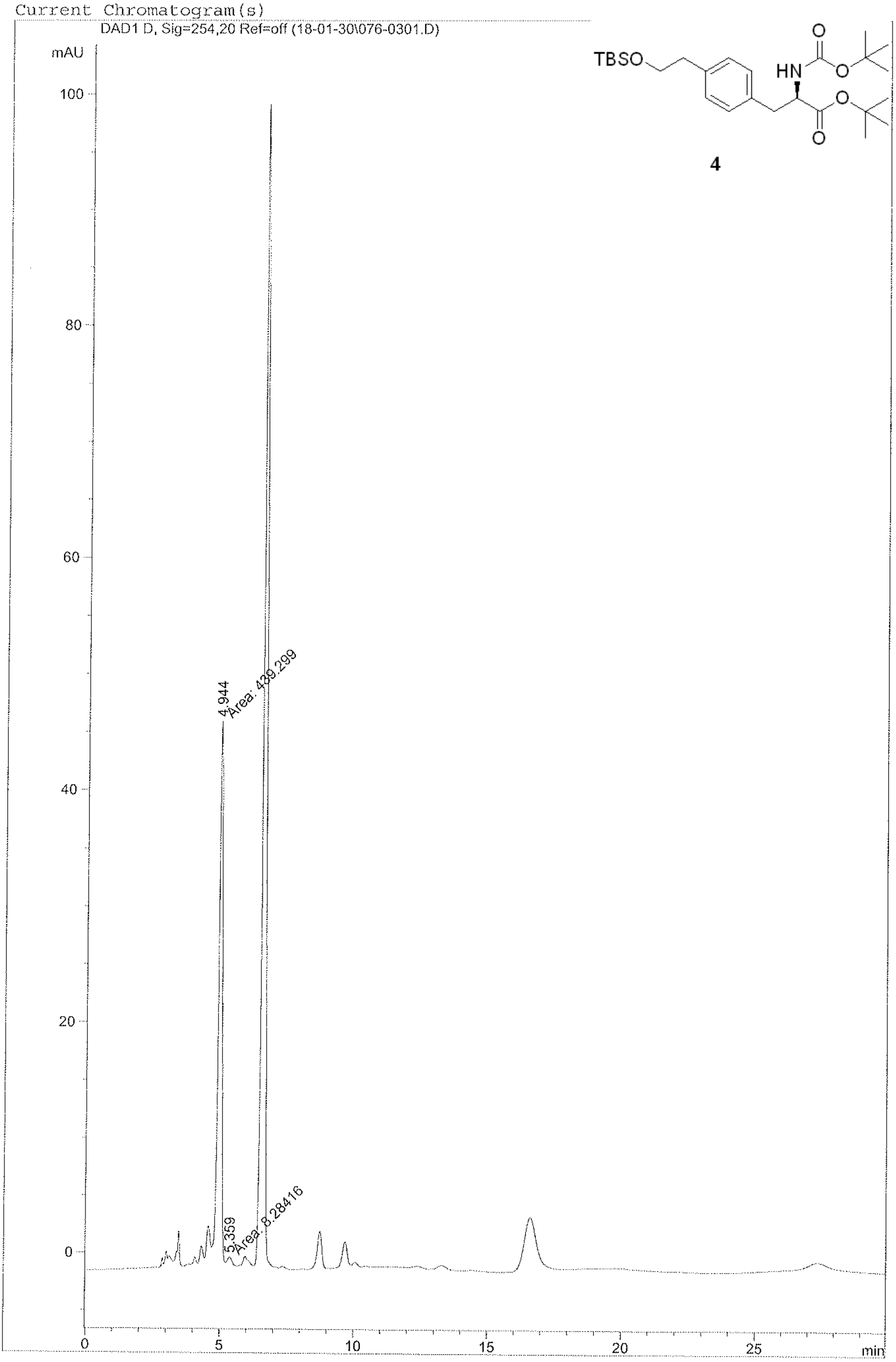
Current Chromatogram(s)
DAD1 D, Sig=254,20 Ref=off (18-01-30\076-0301.D)
mAU
100
80
60
40
20
0
4.944
Area: 439.299
5.359
Area: 8.28416
0
5
10
15
20
25
min
TBSO
HN
O
4

===

Injection Date : 1/30/2018 7:51:39 PM Seq. Line : 3
Sample Name : 14 Location : Vial 76
Acq. Operator : Jan Goeman Inj : 1
Acq. Instrument : Instrument 2 Inj Volume : Inj prog
Acq. Method : C:\HPCHEM\2\METHODS\AB97-3.M
Last changed : 4/11/2016 11:58:26 AM by Jan Goeman
Analysis Method : C:\HPCHEM\1\METHODS\2CD30-70.M
Last changed : 1/26/2018 2:43:53 PM by Jan Goeman
(modified after loading)
kolom 2
10' Spoelen met 30:70
pomp 1ml/min, geen MS

===

Area Percent Report

===

Sorted By : Signal
Multiplier : 1.0000
Dilution : 1.0000

Signal 1: DAD1 A, Sig=195,10 Ref=off

Signal 2: DAD1 B, Sig=210,20 Ref=off

Signal 3: DAD1 C, Sig=214,20 Ref=off

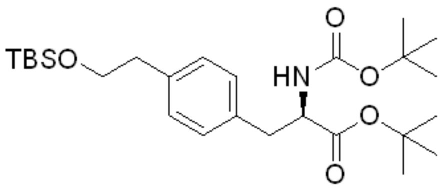

4

Sample: cpd. 4

Signal 4: DAD1 D, Sig=254,20 Ref=off

Peak #	RetTime [min]	Type	Width [min]	Area [mAU*s]	Height [mAU]	Area %
1	4.944	MM	0.1584	439.29868	46.22877	98.1491
2	5.359	MM	0.2010	8.28416	6.86988e-1	1.8509
Totals :				447.58283	46.91576	

Signal 5: DAD1 E, Sig=280,20 Ref=off

===

*** End of Report ***

Chiral purity analysis of compound **9** & **10**

Chromatographic conditions for **9** & **10**:

Diacel Chiralpak IB, 250 x 4.6 mm, particle size 5 µm. Column temperature was set at 35 °C. The chromatographic run was performed isocratically with a mixture of *n*-Hexane/EtOH(abs.) (97/3) and a run length of 30 at a flow rate of 1 mL/min.

Data File D:\DATA\17-12-22\012-0201.D Sample Name: L

===

Injection Date : 12/22/2017 2:06:37 PM Seq. Line : 2
Sample Name : L Location : Vial 12
Acq. Operator : Jan Goeman Inj : 1
Inj Volume : Inj prog

Sequence File : C:\HPCHEM\2\SEQUENCE\TEMP2.S
Method : C:\HPCHEM\2\METHODS\AB97-3.M
Last changed : 4/11/2016 11:58:26 AM by Jan Goeman
Chirale kolom ....
30' isocratisch n-Hexaan-EtOH 97:3

===

Optimization of chiral HPLC
Sample: cpd. 9 & 10

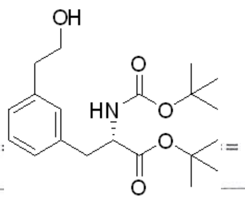

9

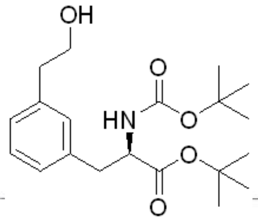

10

DAD1 A, Sig=195,10 Ref=off (17-12-22\012-0201.D)

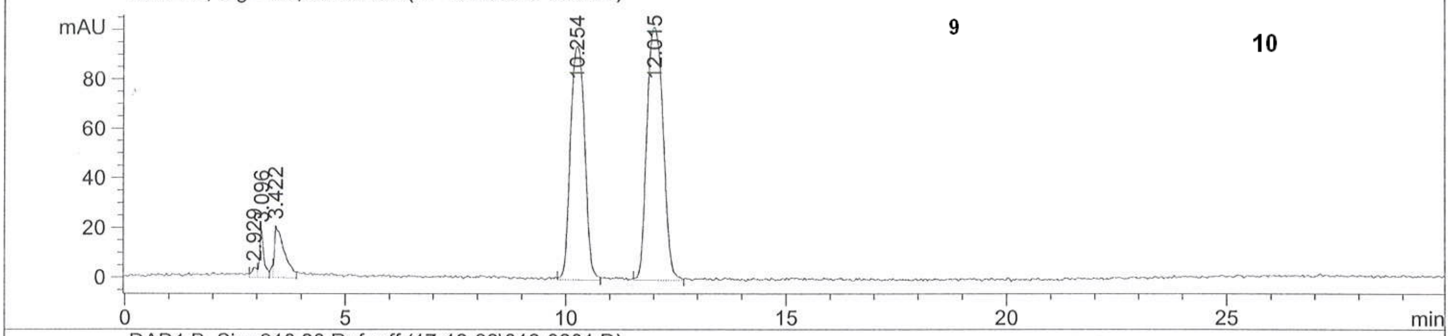

DAD1 B, Sig=210,20 Ref=off (17-12-22\012-0201.D)

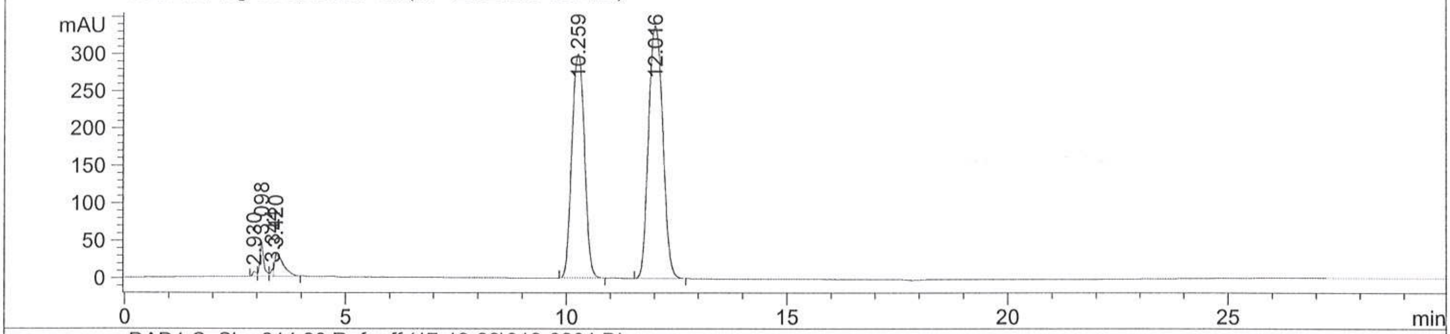

DAD1 C, Sig=214,20 Ref=off (17-12-22\012-0201.D)

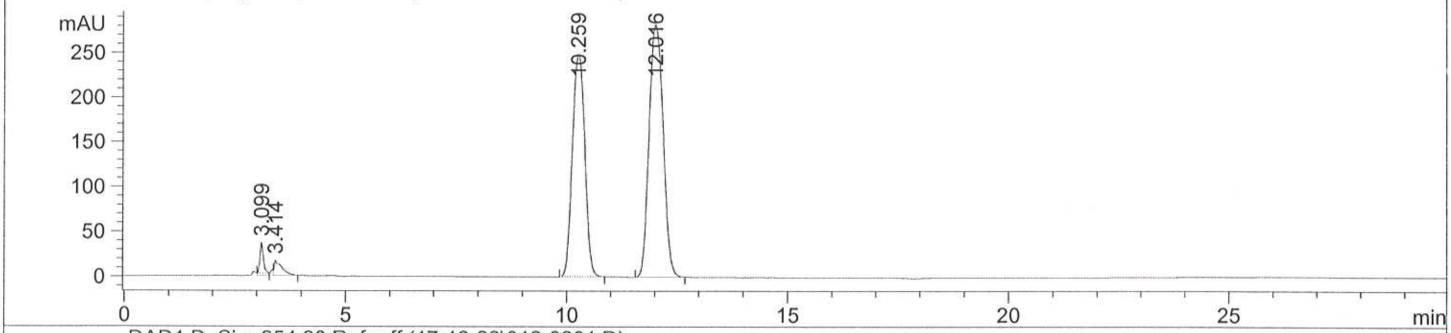

DAD1 D, Sig=254,20 Ref=off (17-12-22\012-0201.D)

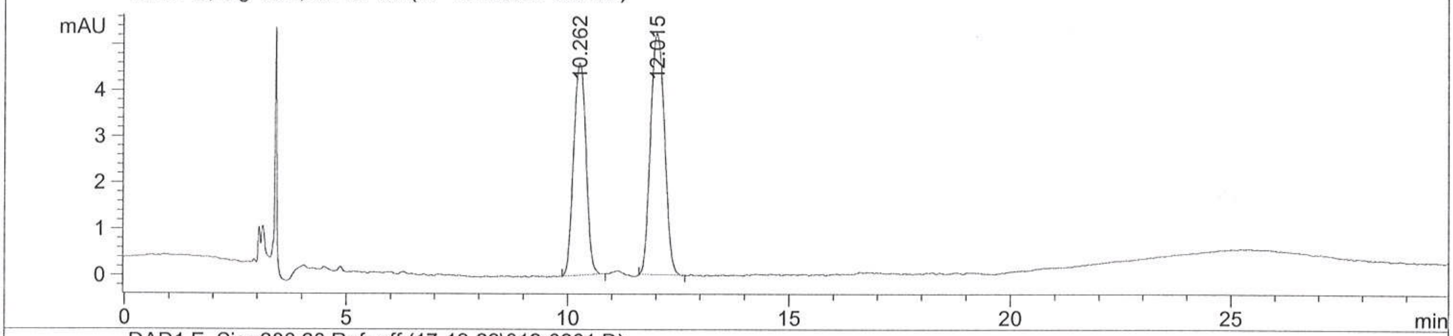

DAD1 E, Sig=280,20 Ref=off (17-12-22\012-0201.D)

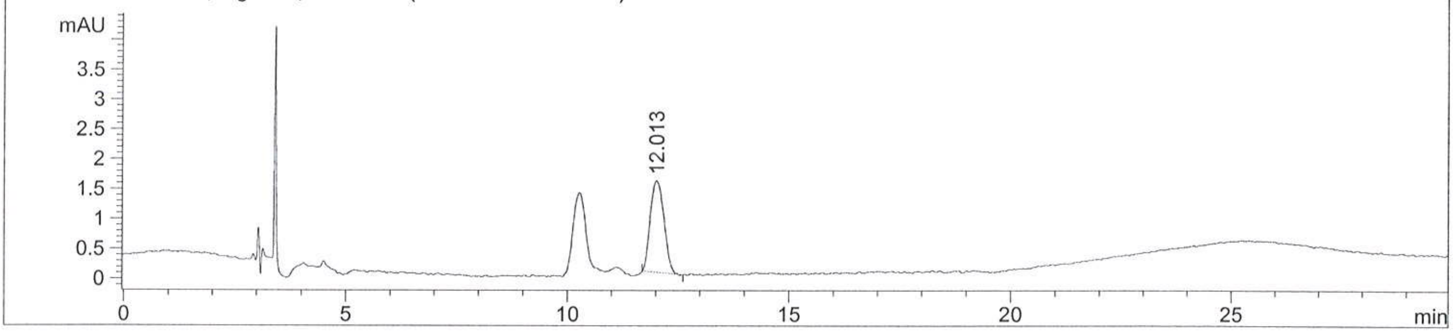

Data File D:\DATA\17-12-22\056-1201.D Sample Name: 23

===

Injection Date : 12/22/2017 7:39:08 PM
Sample Name : 23
Acq. Operator : Jan Goeman
Seq. Line : 12
Location : Vial 56
Inj : 1
Inj Volume : Inj prog

Sequence File : C:\HPCHEM\2\SEQUENCE\TEMP2.S
Method : C:\HPCHEM\2\METHODS\AB97-3.M
Last changed : 4/11/2016 11:58:26 AM by Jan Goeman
Chirale kolom ....
30' isocratisch n-Hexaan-EtOH 97:3

===

Chiral HPLC analysis
Compound 9

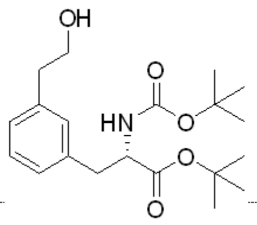

9

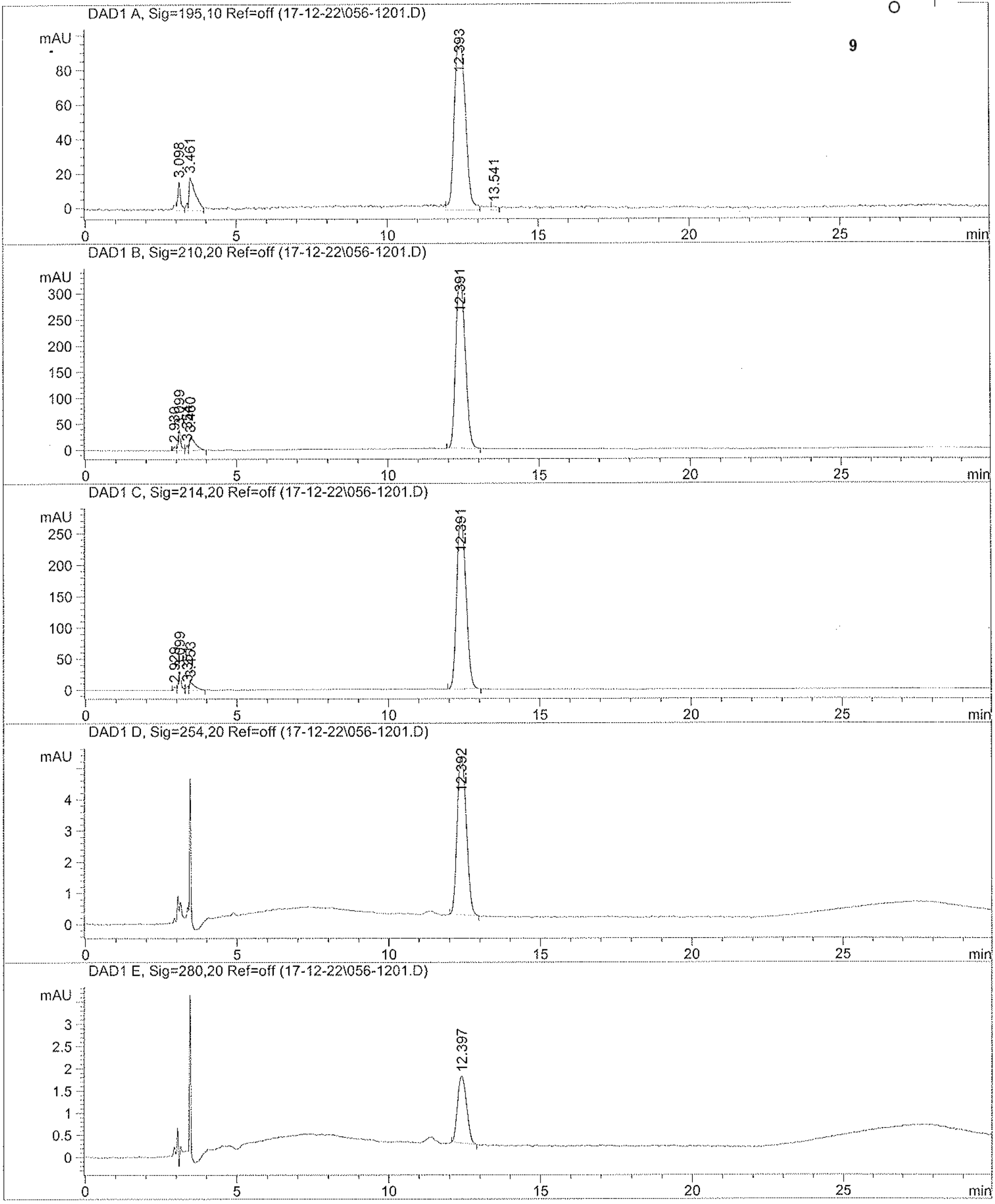

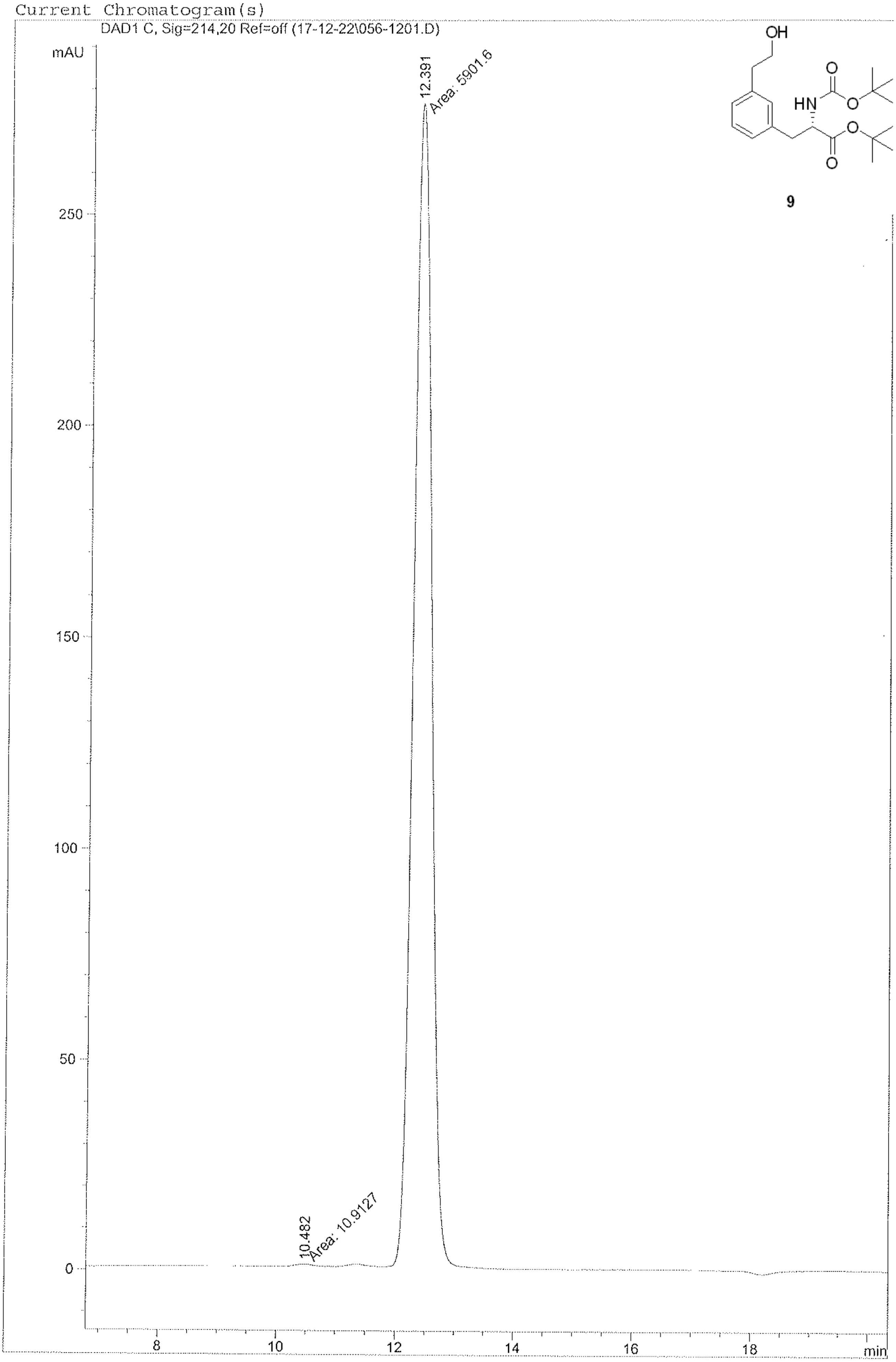
Current Chromatogram(s)
DAD1 C, Sig=214,20 Ref=off (17-12-22\056-1201.D)
mAU
250
200
150
100
50
0
12.391
Area: 5901.6
10.482
Area: 10.9127
8
10
12
14
16
18
min
OH
HN
O
9

===

Injection Date : 12/22/2017 7:39:08 PM Seq. Line : 12
Sample Name : 23 Location : Vial 56
Acq. Operator : Jan Goeman Inj : 1
Acq. Instrument : Instrument 2 Inj Volume : Inj prog
Acq. Method : C:\HPCHEM\2\METHODS\AB97-3.M
Last changed : 4/11/2016 11:58:26 AM by Jan Goeman
Analysis Method : C:\HPCHEM\1\METHODS\2CD30-70.M
Last changed : 1/26/2018 2:43:53 PM by Jan Goeman
(modified after loading)
kolom 2
10' Spoelen met 30:70
pomp 1ml/min, geen MS

===

Area Percent Report

===

Sorted By : Signal
Multiplier : 1.0000
Dilution : 1.0000

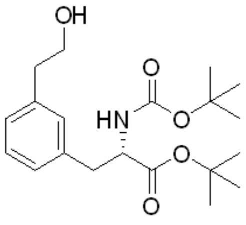

9

Signal 1: DAD1 C, Sig=214,20 Ref=off

Peak #	RetTime [min]	Type	Width [min]	Area [mAU*s]	Height [mAU]	Area %
1	2.929	BV	0.0725	30.39187	6.06415	0.4805
2	3.099	VV	0.0890	181.64526	30.73150	2.8720
3	3.351	VV	0.0742	33.01746	6.85152	0.5220
4	3.453	VB	0.1459	167.21483	14.57481	2.6438
5	10.482	MM	0.3339	10.91270	5.44788e-1	0.1725
6	12.391	MM	0.3567	5901.60303	275.75787	93.3091

Totals : 6324.78515 334.52464

===

*** End of Report ***

```
=====================================================================
Injection Date  : 12/22/2017 8:14:01 PM          Seq. Line :   13
Sample Name     : 24                              Location : Vial 57
Acq. Operator   : Jan Goeman                           Inj :   1
                                                Inj Volume : Inj prog

Sequence File   : C:\HPCHEM\2\SEQUENCE\TEMP2.S
Method          : C:\HPCHEM\2\METHODS\AB97-3.M
Last changed    : 4/11/2016 11:58:26 AM by Jan Goeman
Chirale kolom ....................
30' isocratisch n-Hexaan-EtOH 97:3
=====================================================================
```

Chiral HPLC analysis
Compound 10

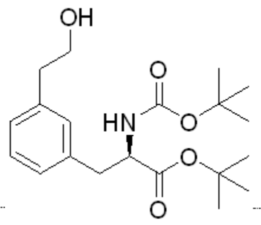

10

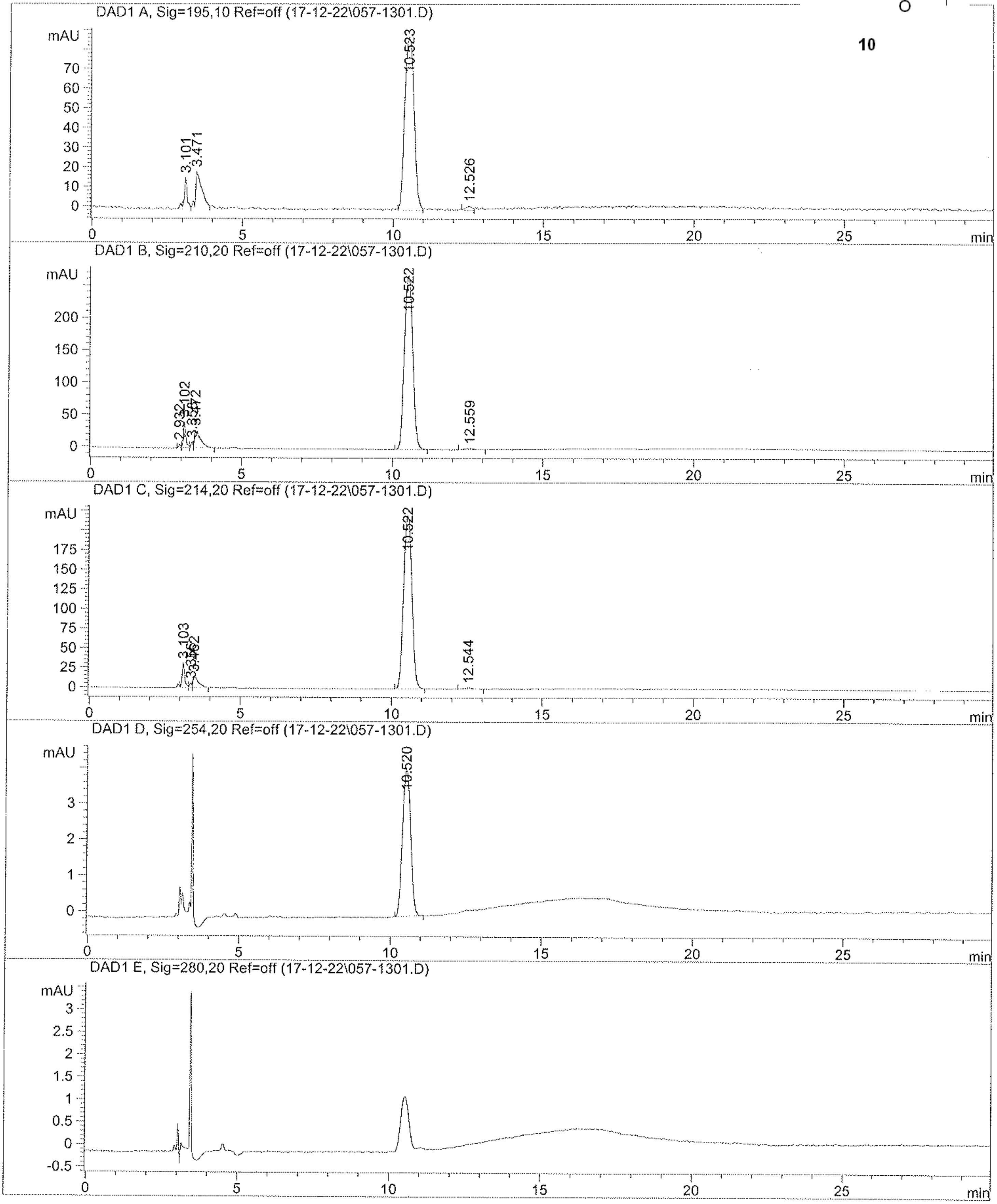

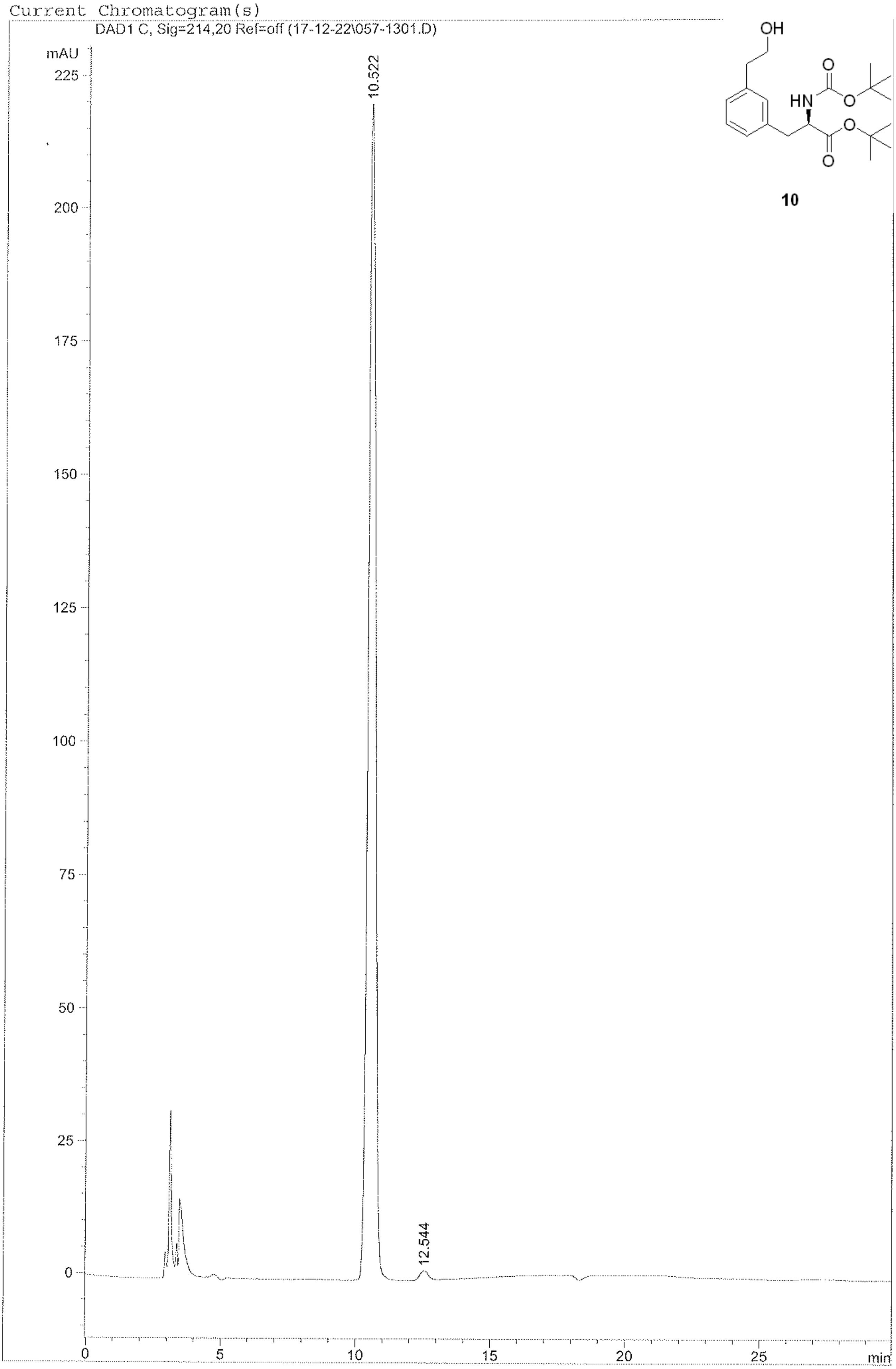
Current Chromatogram(s)
DAD1 C, Sig=214,20 Ref=off (17-12-22\057-1301.D)
mAU
225
200
175
150
125
100
75
50
25
0
10.522
12.544
0
5
10
15
20
25
min
OH
HN
O
O
O
O

10

===

Injection Date : 12/22/2017 8:14:01 PM Seq. Line : 13
Sample Name : 24 Location : Vial 57
Acq. Operator : Jan Goeman Inj : 1
Acq. Instrument : Instrument 2 Inj Volume : Inj prog
Acq. Method : C:\HPCHEM\2\METHODS\AB97-3.M
Last changed : 4/11/2016 11:58:26 AM by Jan Goeman
Analysis Method : C:\HPCHEM\1\METHODS\2CD30-70.M
Last changed : 1/26/2018 2:43:53 PM by Jan Goeman
(modified after loading)
kolom 2
10' Spoelen met 30:70
pomp 1ml/min, geen MS

===

Area Percent Report

===

Sorted By : Signal
Multiplier : 1.0000
Dilution : 1.0000

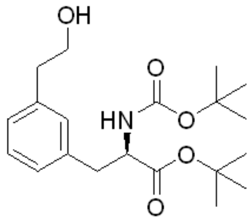

10

Signal 1: DAD1 C, Sig=214,20 Ref=off

Peak #	RetTime [min]	Type	Width [min]	Area [mAU*s]	Height [mAU]	Area %
1	10.522	BB	0.3103	4241.71045	220.65605	99.1330
2	12.544	PB	0.3225	37.09894	1.75574	0.8670

Totals : 4278.80939 222.41179

===

*** End of Report ***

Chiral purity analysis of compound **15** & **16**

Chromatographic conditions for **15** & **16**:

Chiral capillary electrophoresis (CE) employed a 64.5 cm capillary (50 µm diameter). The electrolyte used consisted of 25 mM phosphate buffer pH 2.5 + 5% HS-gammaCD chiral modifier. Analysis time was 40 min at -20 kV.

Data File D:\DATA\18-04-18\039-0401.D

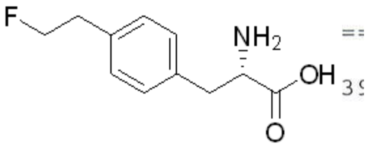

15

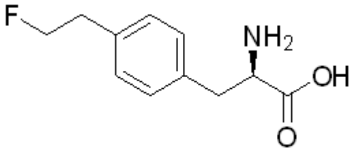

16

```
=====================================================================
Injection Date   : 4/18/18 9:35:04 PM
Sample Name      : 19+21
Acq. Operator    : Jan Goeman
Sequence File    : C:\HPCHEM\1\SEQUENCE\TEMP.S
Method           : C:\HPCHEM\1\METHODS\CE\01-4
Last changed     : 5/11/11 3:19:59 PM by Jan G
CE met buffer 1
L=.64,5.cm / d=..50.um / pH.2,5. .25..mM / SDS..-...mM / HS-γ.CD .5...%
=====================================================================
```

Optimization of CE analysis
Sample: cpd. 15 & 16

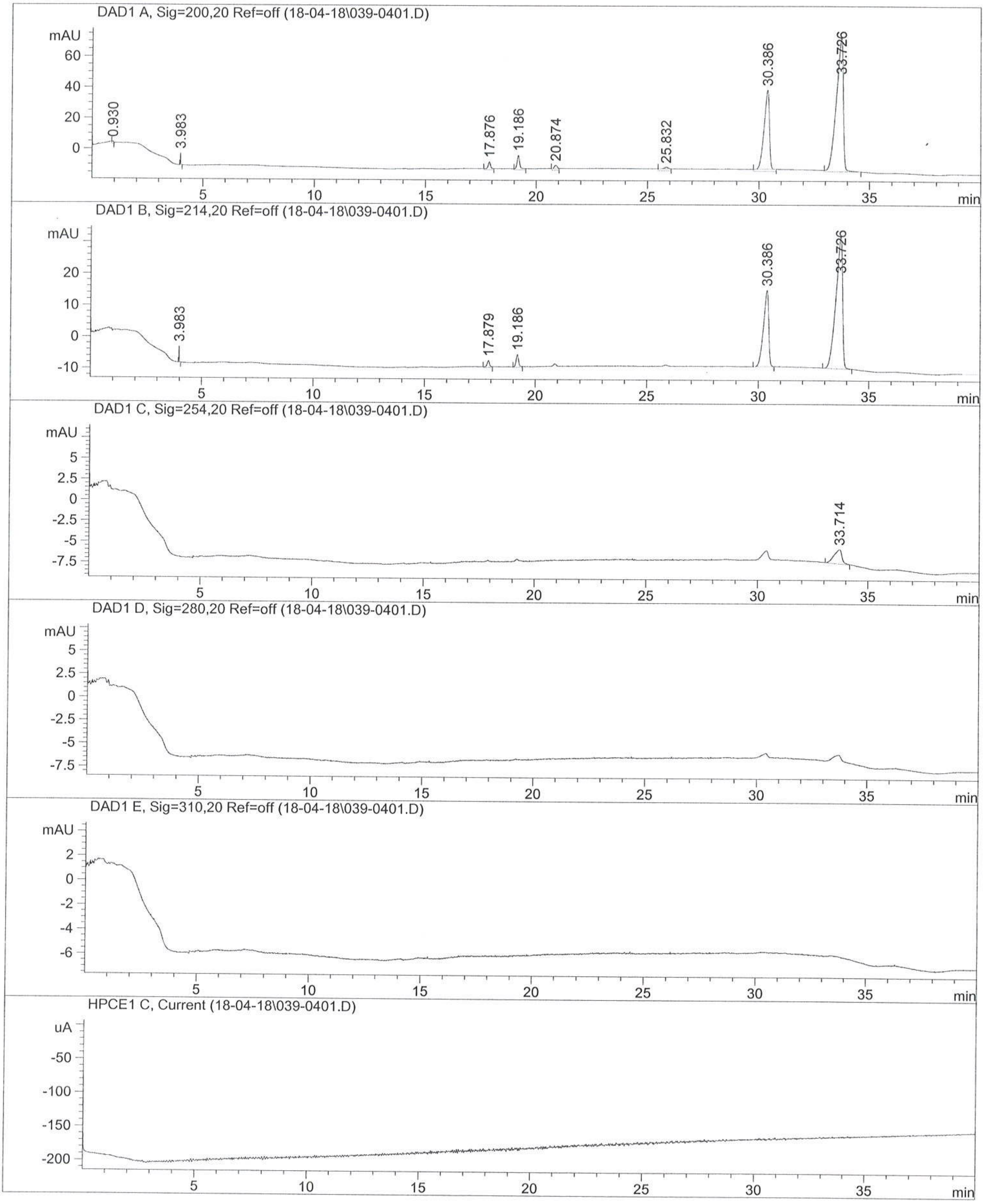

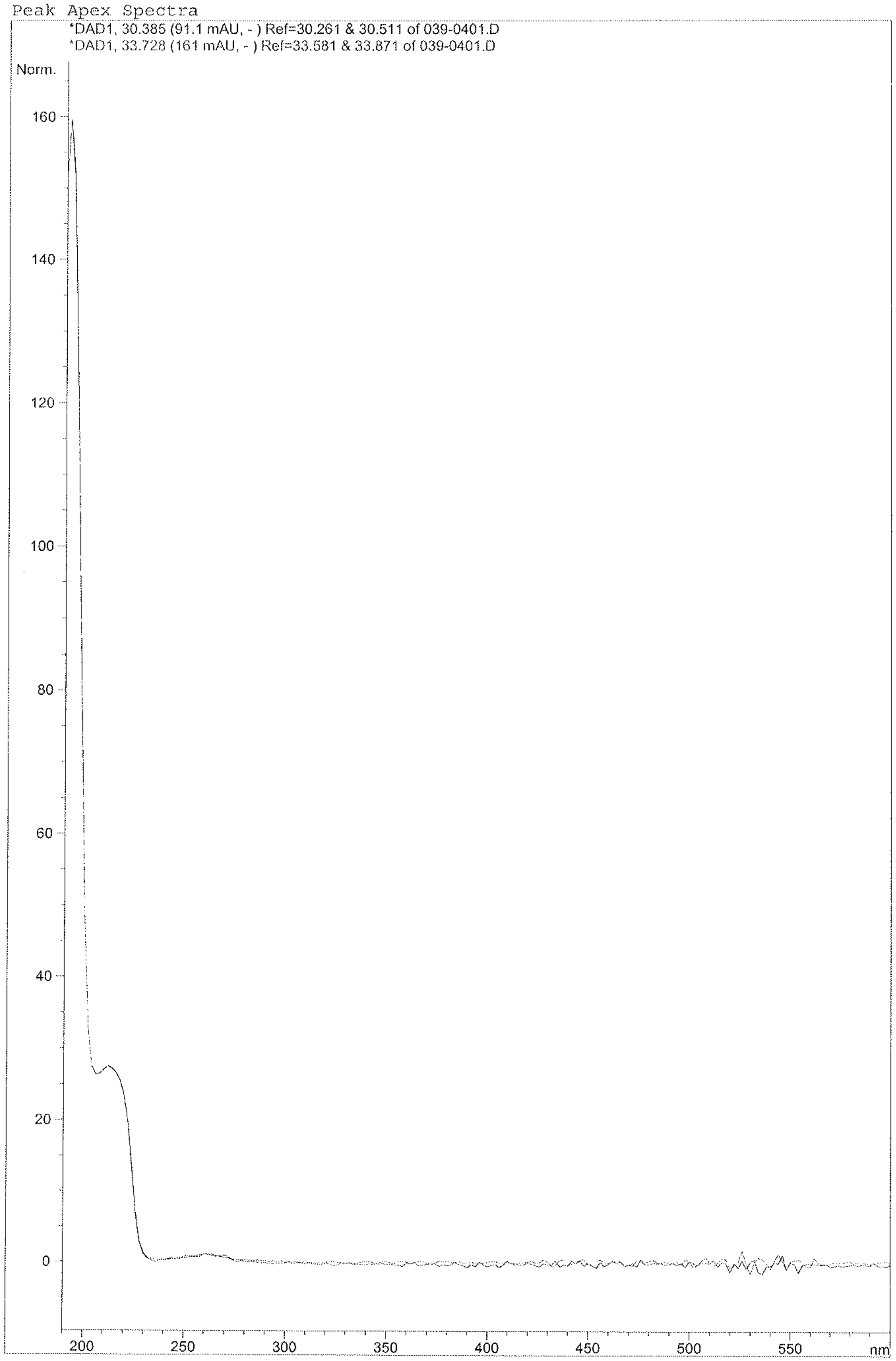
Peak Apex Spectra
*DAD1, 30.385 (91.1 mAU, -) Ref=30.261 & 30.511 of 039-0401.D
*DAD1, 33.728 (161 mAU, -) Ref=33.581 & 33.871 of 039-0401.D
Norm.
160
140
120
100
80
60
40
20
0
200
250
300
350
400
450
500
550
nm

Data File D:\DATA\18-04-18\037-0501.D

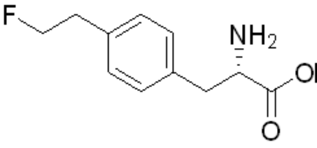

15

Sample: cpd. 15

```
=====================================================================
Injection Date    : 4/18/18 10:19:20 PM            Seq. Line :    5
Sample Name       : 19                             Location : Vial 37
Acq. Operator     : Jan Goeman                          Inj :    1
Sequence File     : C:\HPCHEM\1\SEQUENCE\TEMP.S
Method            : C:\HPCHEM\1\METHODS\CE\01-40N20.M
Last changed      : 5/11/11 3:19:59 PM by Jan Goeman
CE met buffer 1
L=64,5.cm / d=.50.um / pH.2,5. .25...mM / SDS..-..mM / HS.γ.CD ..5...%
=====================================================================
```

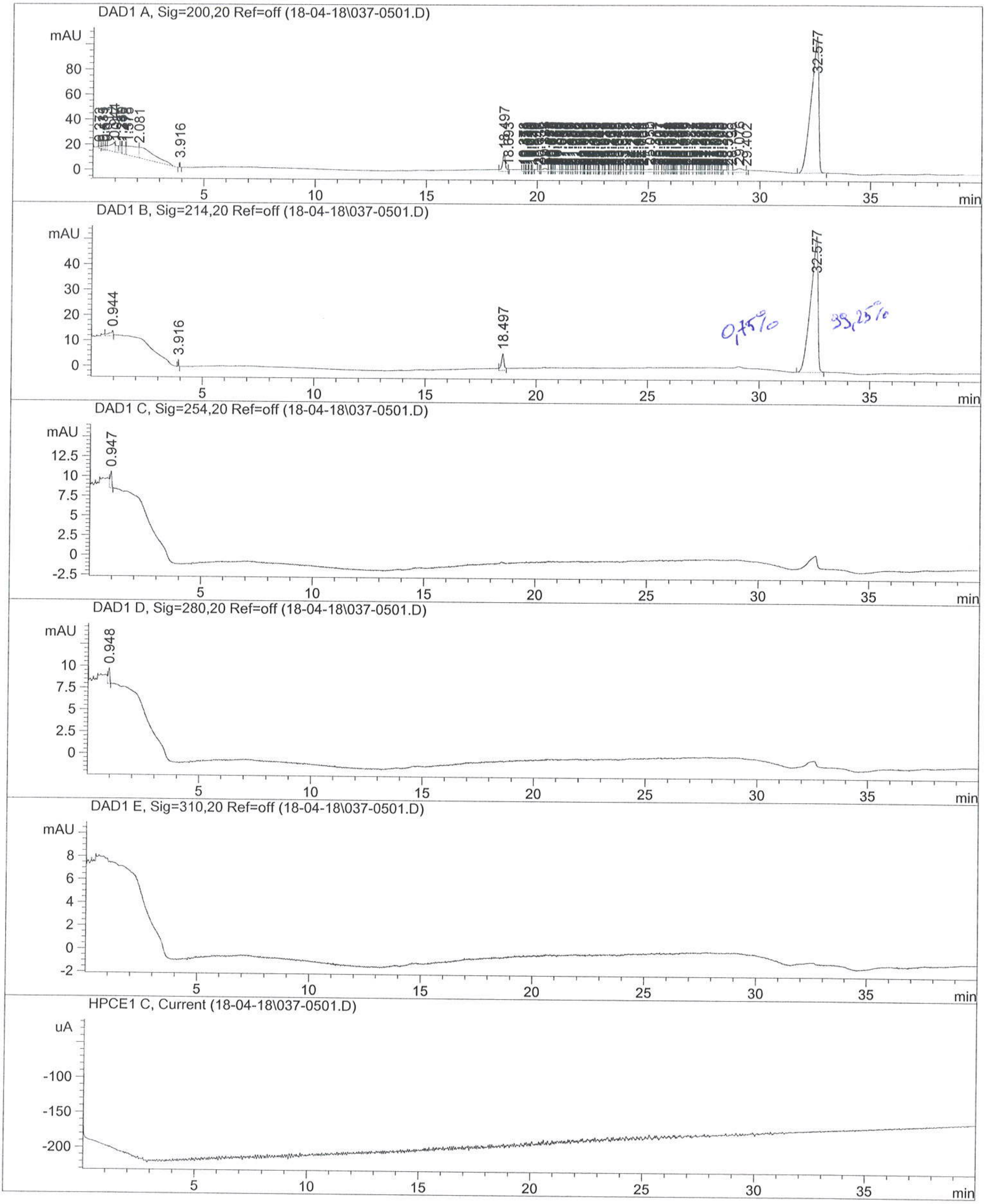

Current Electropherogram(s)

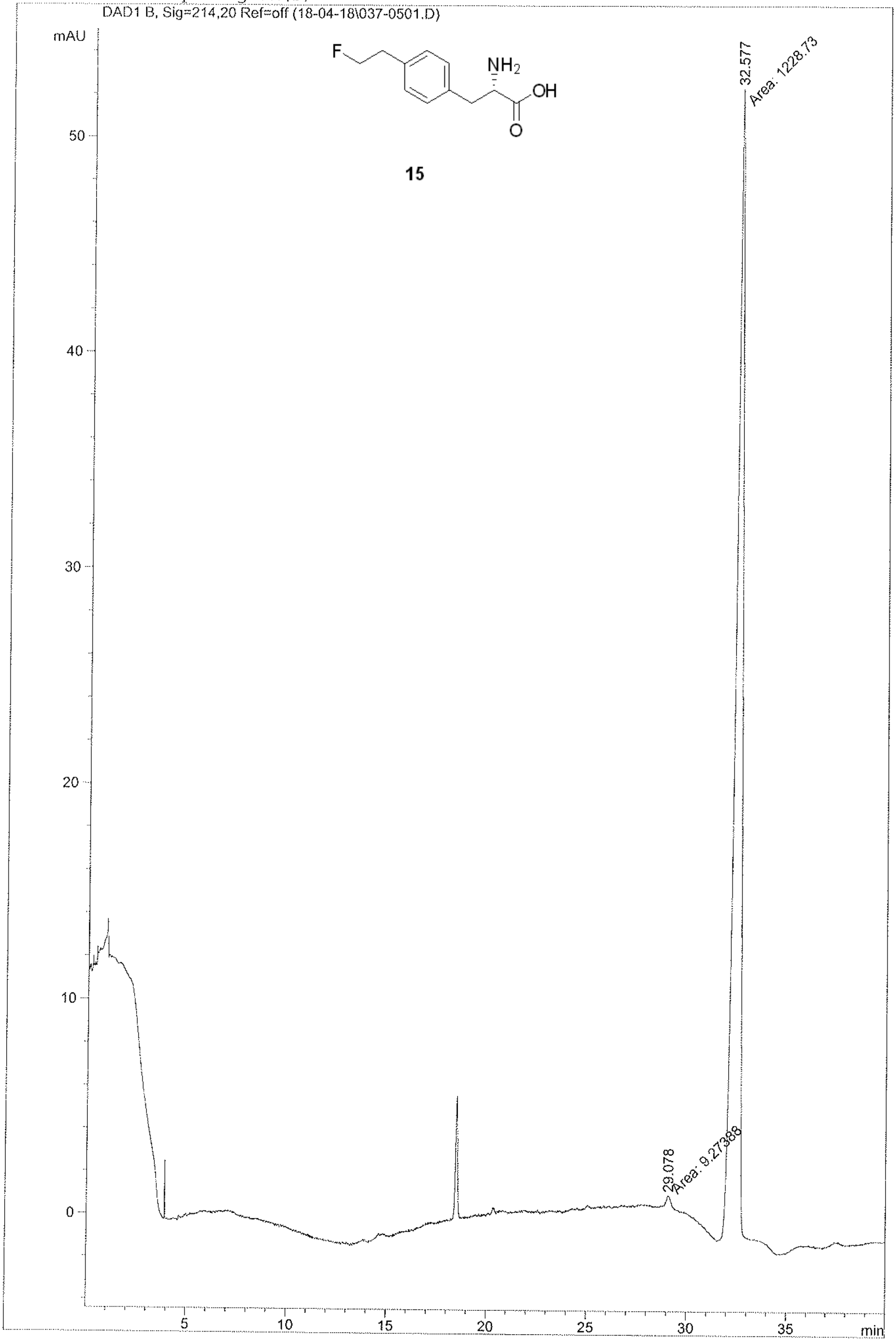

===
Injection Date : 4/18/18 10:19:20 PM Seq. Line : 5
Sample Name : 19 Location : Vial 37
Acq. Operator : Jan Goeman Inj : 1
Acq. Method : C:\HPCHEM\1\METHODS\CE\01-40N20.M
Last changed : 5/11/11 3:19:59 PM by Jan Goeman
Analysis Method : C:\HPCHEM\1\METHODS\CE\DEF_CE.M
Last changed : 12/15/10 2:53:13 PM by Jan Goeman
Default Method (no analysis)
===
Area Percent Report
===

Sorted By : Signal
Area Calculation Mode : Measured Area
Multiplier : 1.0000
Dilution : 1.0000

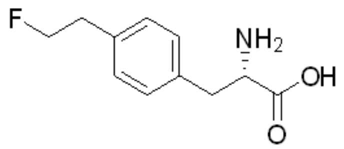

15

Signal 1: DAD1 B, Sig=214,20 Ref=off

Peak #	MigTime [min]	Type	Width [min]	Area [mAU*s]	Height [mAU]	Area %
1	29.078	MM	0.2730	9.27388	5.66264e-1	0.7491
2	32.577	MM	0.3840	1228.72766	53.33382	99.2509

Analysis cpd. 15

Totals : 1238.00154 53.90008

Results obtained with enhanced integrator! (parabolic interpolation)
===
*** End of Report ***

Data File D:\DATA\18-04-18\038-0601.D Sample Name: 20

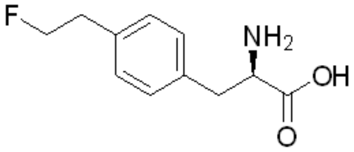

16

Sample: cpd. 16

===

Injection Date	:	4/18/18 11:03:41 PM	Seq. Line	:	6
Sample Name	:	20	Location	:	Vial 38
Acq. Operator	:	Jan Goeman	Inj	:	1
Sequence File	:	C:\HPCHEM\1\SEQUENCE\TEMP.S			
Method	:	C:\HPCHEM\1\METHODS\CE\01-40N20.M			
Last changed	:	5/11/11 3:19:59 PM by Jan Goeman			

CE met buffer 1

L=.64,5.cm / d=.50..um / pH.2,5.. .25..mM / SDS. ∕ ...mM / .HS γ.CD .5....%

===

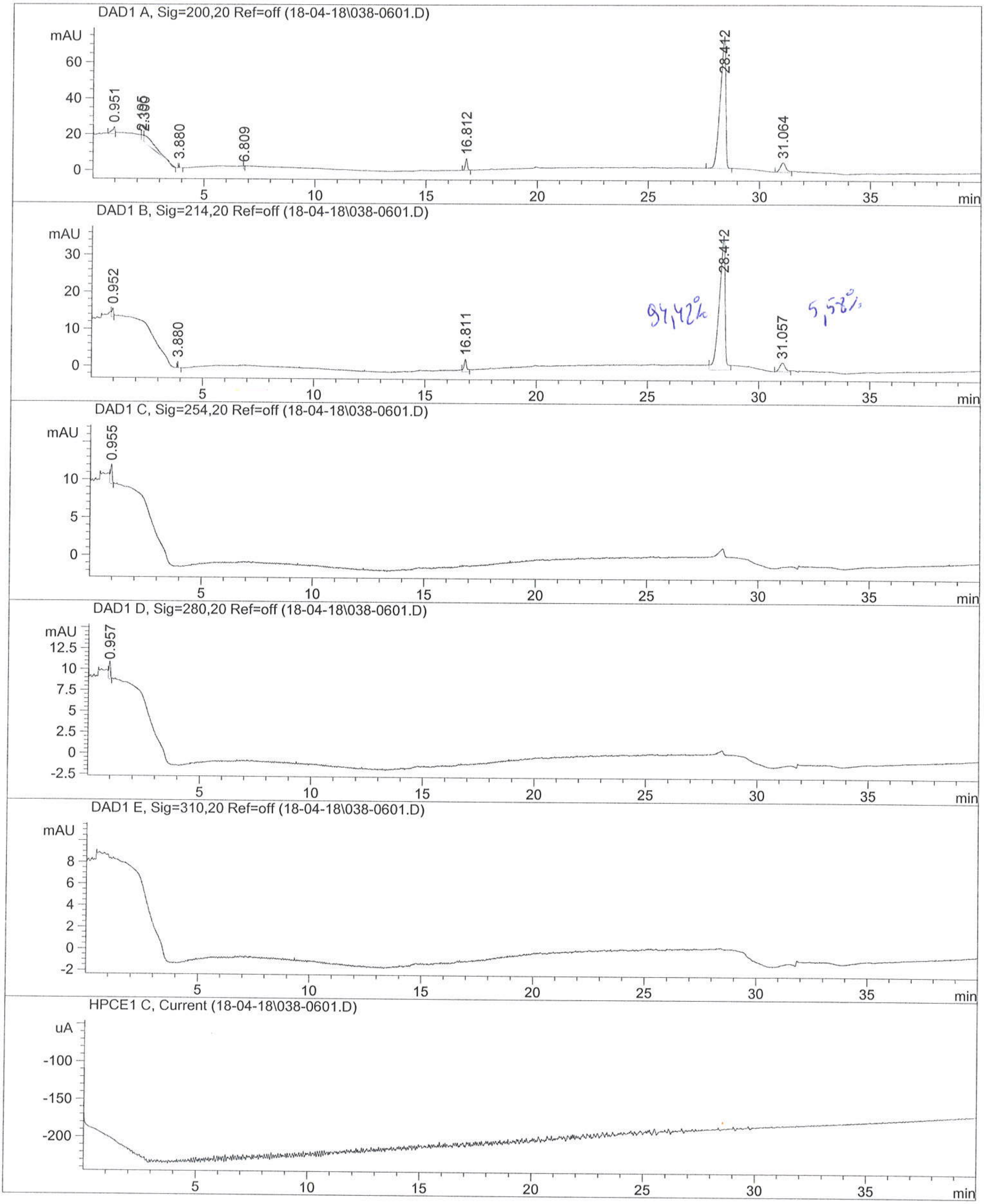

Current Electropherogram(s)

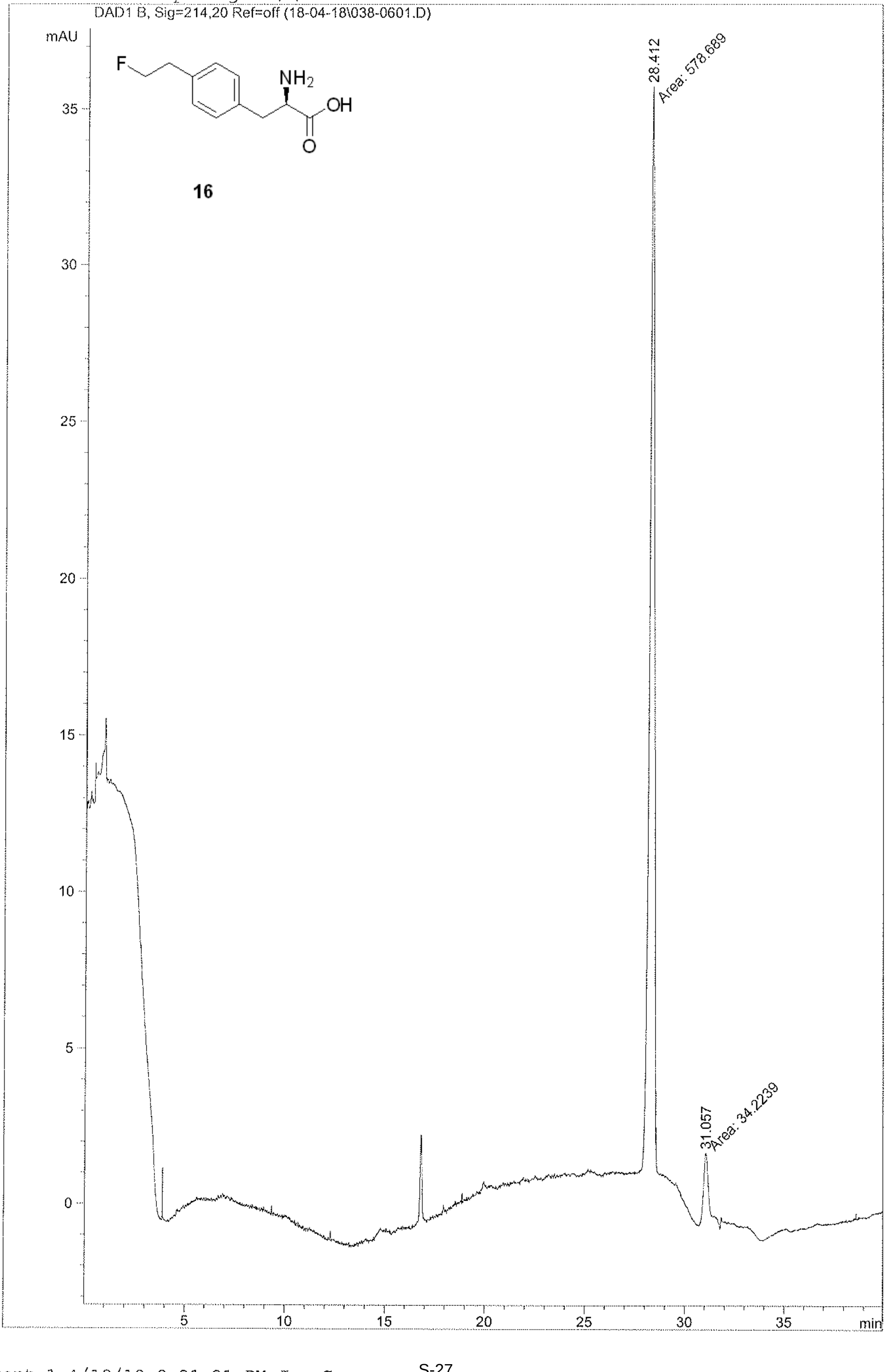

===

Injection Date : 4/18/18 11:03:41 PM Seq. Line : 6
Sample Name : 20 Location : Vial 38
Acq. Operator : Jan Goeman Inj : 1
Acq. Method : C:\HPCHEM\1\METHODS\CE\01-40N20.M
Last changed : 5/11/11 3:19:59 PM by Jan Goeman
Analysis Method : C:\HPCHEM\1\METHODS\CE\DEF_CE.M
Last changed : 12/15/10 2:53:13 PM by Jan Goeman
Default Method (no analysis)

===

Area Percent Report

===

Sorted By : Signal
Area Calculation Mode : Measured Area
Multiplier : 1.0000
Dilution : 1.0000

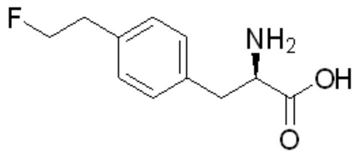

16

Signal 1: DAD1 B, Sig=214,20 Ref=off

Peak #	MigTime [min]	Type	Width [min]	Area [mAU*s]	Height [mAU]	Area %
1	28.412	MM	0.2778	578.68945	34.71428	94.4162
2	31.057	MM	0.2610	34.22391	2.18517	5.5838

Analysis cpd. 16

Totals : 612.91336 36.89945

Results obtained with enhanced integrator! (parabolic interpolation)

===

*** End of Report ***

Chiral purity analysis of compound **17** & **18**

Chromatographic conditions for **17** & **18**:

Chiral capillary electrophoresis (CE) employed a 64.5 cm capillary (50 µm diameter). The electrolyte used consisted of 25 mM phosphate buffer pH 2.5 + 5% HS-gammaCD chiral modifier. Analysis time was 40 min at -20 kV.

Data File D:\DATA\18-04-18\042-0701.D

===

Injection Date	: 4/19/18 11:47:57 PM	Seq. L
Sample Name	: 29+31	Locat
Acq. Operator	: Jan Goeman	
Sequence File	: C:\HPCHEM\1\SEQUENCE\TEMP.S	
Method	: C:\HPCHEM\1\METHODS\CE\01-40N20.M	
Last changed	: 5/11/11 3:19:59 PM by Jan Goeman	

CE met buffer 1

L=.64,5.cm / d=.50.um / pH.2,5. .25..mM / SDS.-...mM / .HS.γCD ..5..%

===

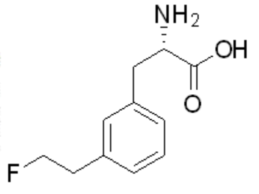

17

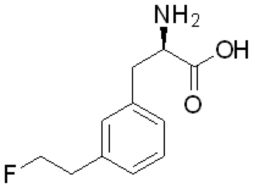

18

Sa

Optimization of chiral CE
Sample: cpd. 17 & 18

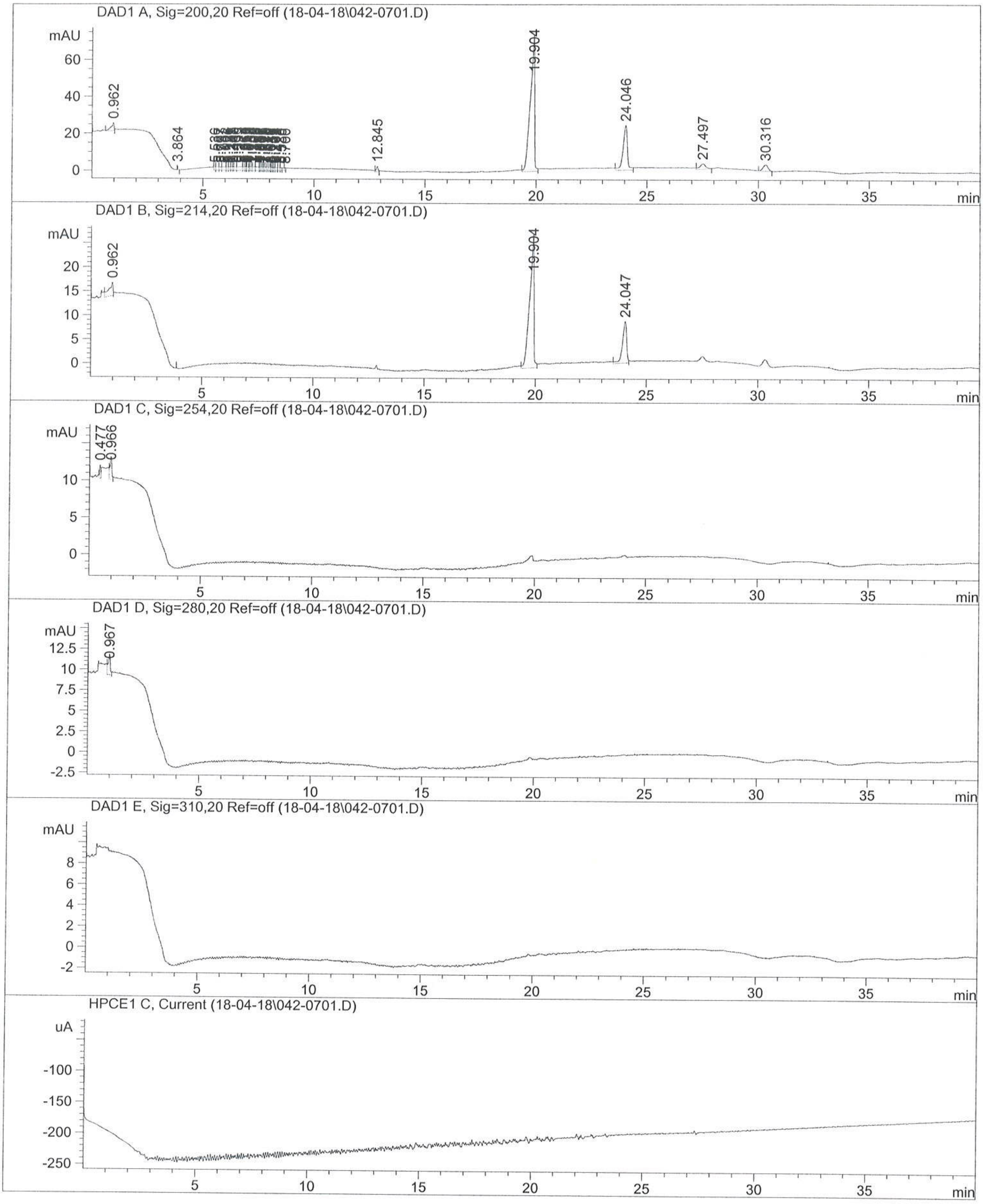

Peak Apex Spectra

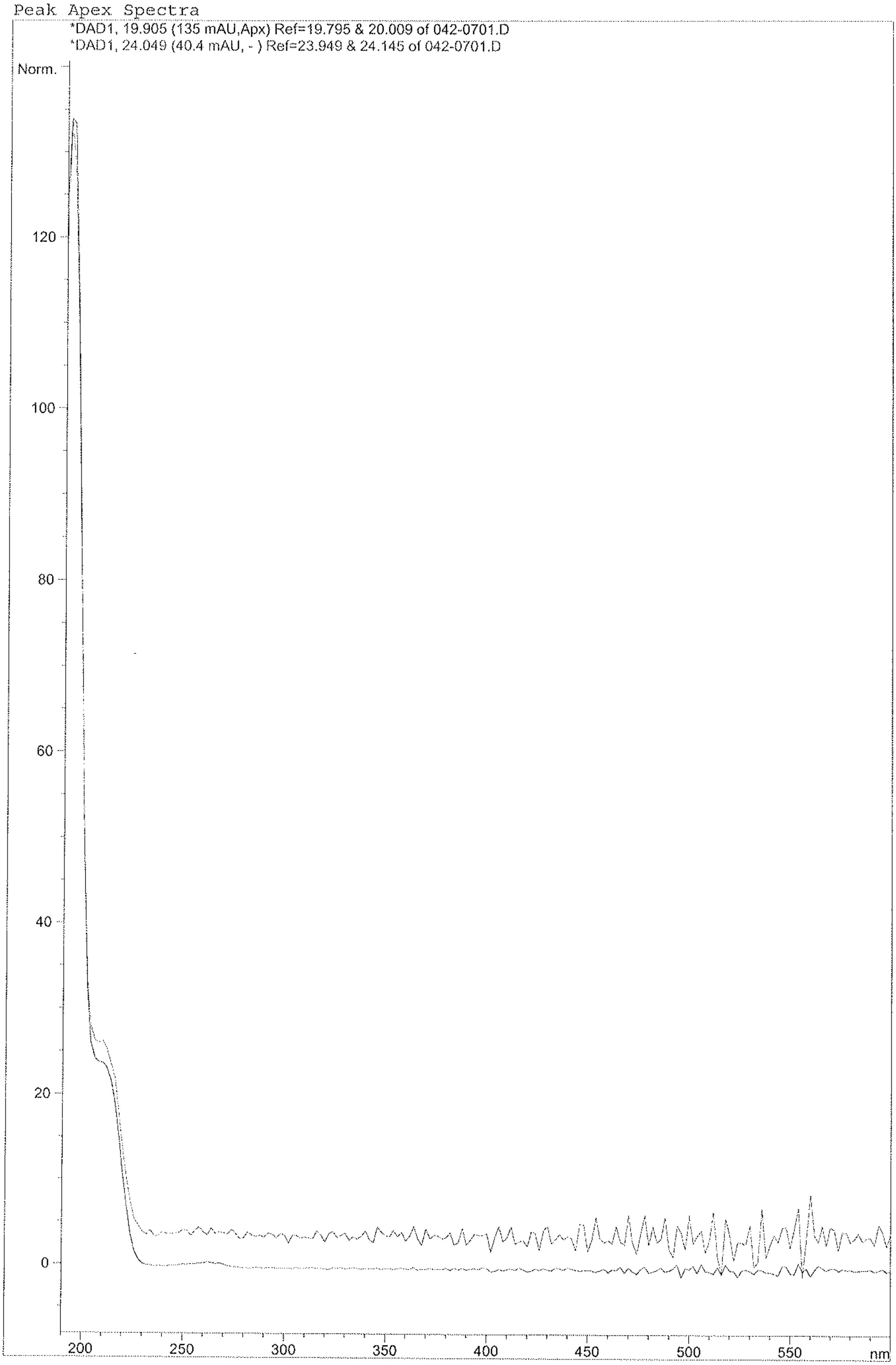

Data File D:\DATA\18-04-18\040-0801.D Sample Name: 29

Injection Date	: 4/19/18 12:32:19 AM	Seq. Line :	8
Sample Name	: 29	Location :	Vial 40
Acq. Operator	: Jan Goeman	Inj :	1
Sequence File	: C:\HPCHEM\1\SEQUENCE\TEMP.S		
Method	: C:\HPCHEM\1\METHODS\CE\01-40N20.M		
Last changed	: 5/11/11 3:19:59 PM by Jan Goeman		

CE met buffer 1
L=.64.5.cm / d=.50.um / pH.2,5. .25..mM / SDS.—...mM / HS γ.CD ..5...%

CE analysis
Sample: cpd. 17

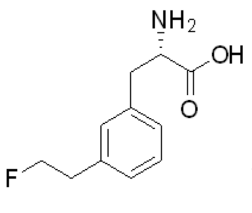

17

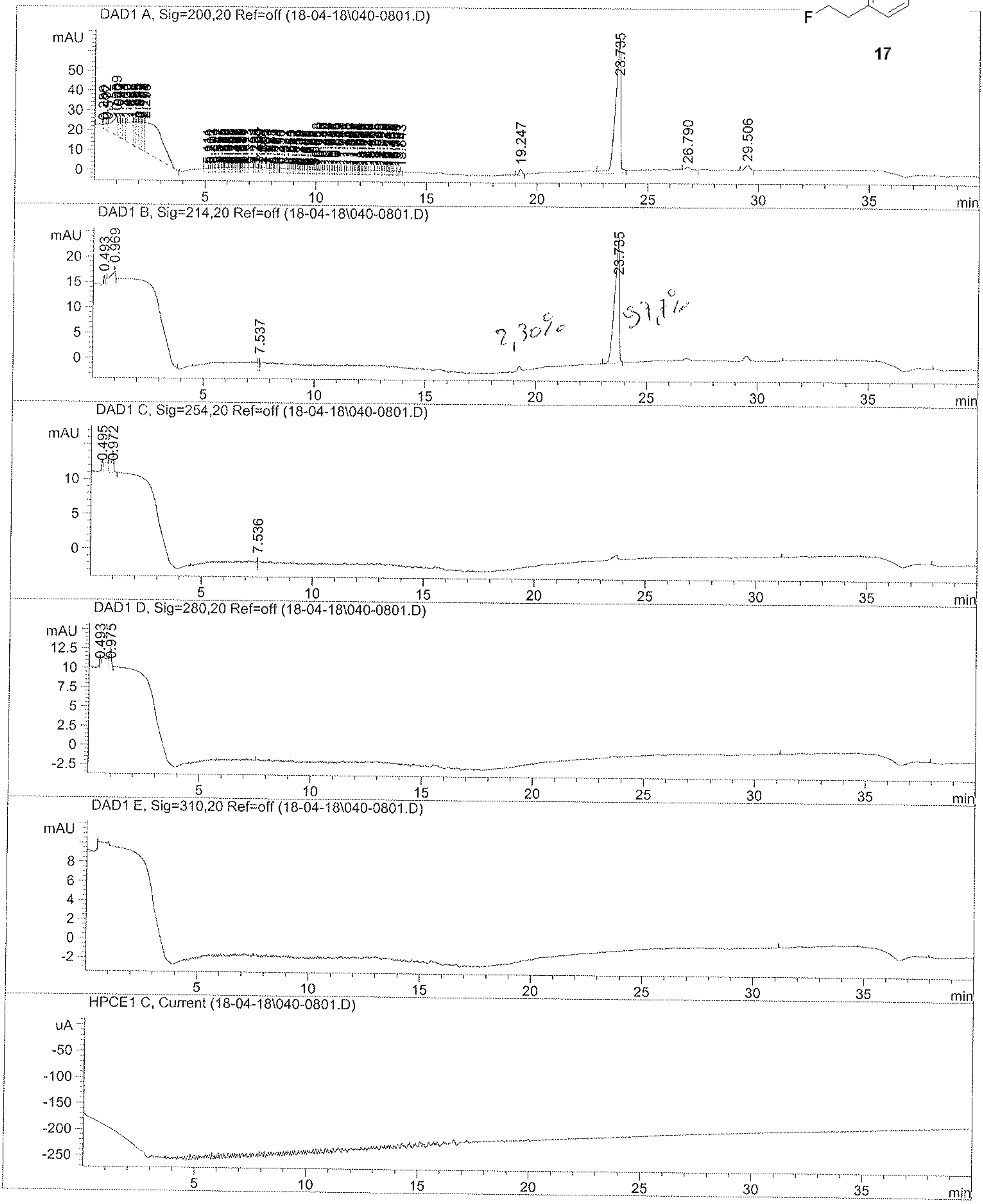

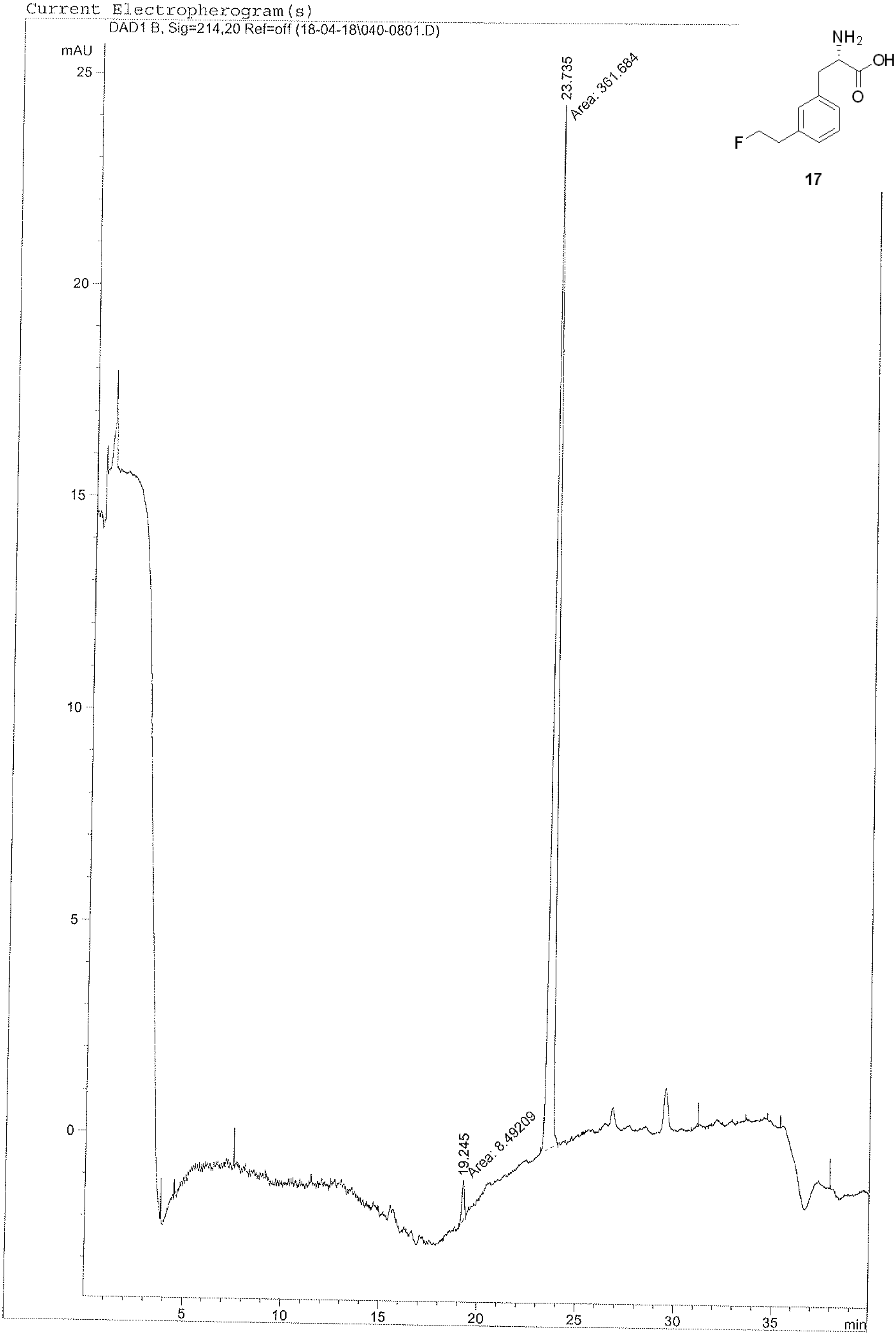
Current Electropherogram(s)
DAD1 B, Sig=214,20 Ref=off (18-04-18\040-0801.D)
mAU
25
20
15
10
5
0
23.735
Area: 361.684
19.245
Area: 8.49209
5
10
15
20
25
30
35
min
NH2
OH
O
F

17

===

Injection Date : 4/19/18 12:32:19 AM Seq. Line : 8
Sample Name : 29 Location : Vial 40
Acq. Operator : Jan Goeman Inj : 1
Acq. Method : C:\HPCHEM\1\METHODS\CE\01-40N20.M
Last changed : 5/11/11 3:19:59 PM by Jan Goeman
Analysis Method : C:\HPCHEM\1\METHODS\CE\DEF_CE.M
Last changed : 12/15/10 2:53:13 PM by Jan Goeman
Default Method (no analysis)

===

Area Percent Report

===

Sorted By : Signal
Area Calculation Mode : Measured Area
Multiplier : 1.0000
Dilution : 1.0000

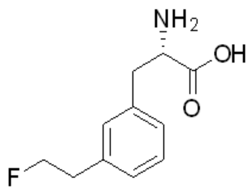

17

Signal 1: DAD1 B, Sig=214,20 Ref=off

Peak #	MigTime [min]	Type	Width [min]	Area [mAU*s]	Height [mAU]	Area %
1	19.245	MM	0.1443	8.49209	9.80606e-1	2.2941
2	23.735	MM	0.2453	361.68427	24.57265	97.7059

Analysis cpd. 17

Totals : 370.17636 25.55326

Results obtained with enhanced integrator! (parabolic interpolation)

===

*** End of Report ***

Data File D:\DATA\18-04-18\041-0901.D

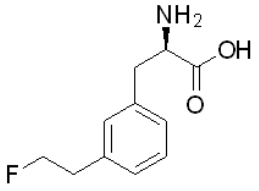

18

Chiral CE analysis
Sample: cpd. 18

```
=====================================================================
Injection Date  : 4/19/18 1:16:34 AM          Seq. Line :    9
Sample Name     : 30                          Location : Vial 41
Acq. Operator   : Jan Goeman                       Inj :    1
Sequence File   : C:\HPCHEM\1\SEQUENCE\TEMP.S
Method          : C:\HPCHEM\1\METHODS\CE\01-40N20.M
Last changed    : 5/11/11 3:19:59 PM by Jan Goeman
CE met, buffer 1
L=.64.5.cm / d=.52.um / pH.2.5.. 25...mM / SDS -....mM / HS-γ.CD ..5...%
=====================================================================
```

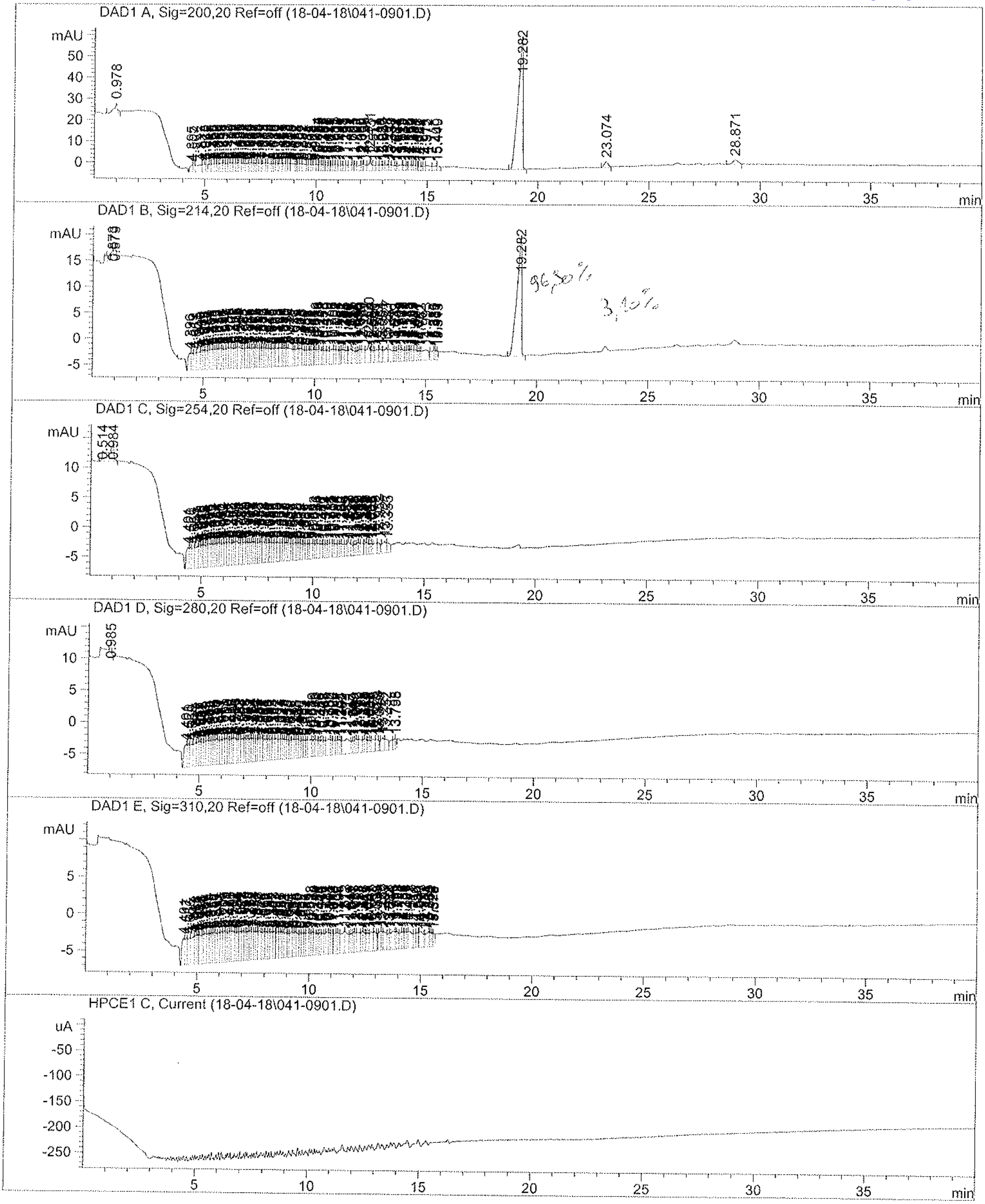

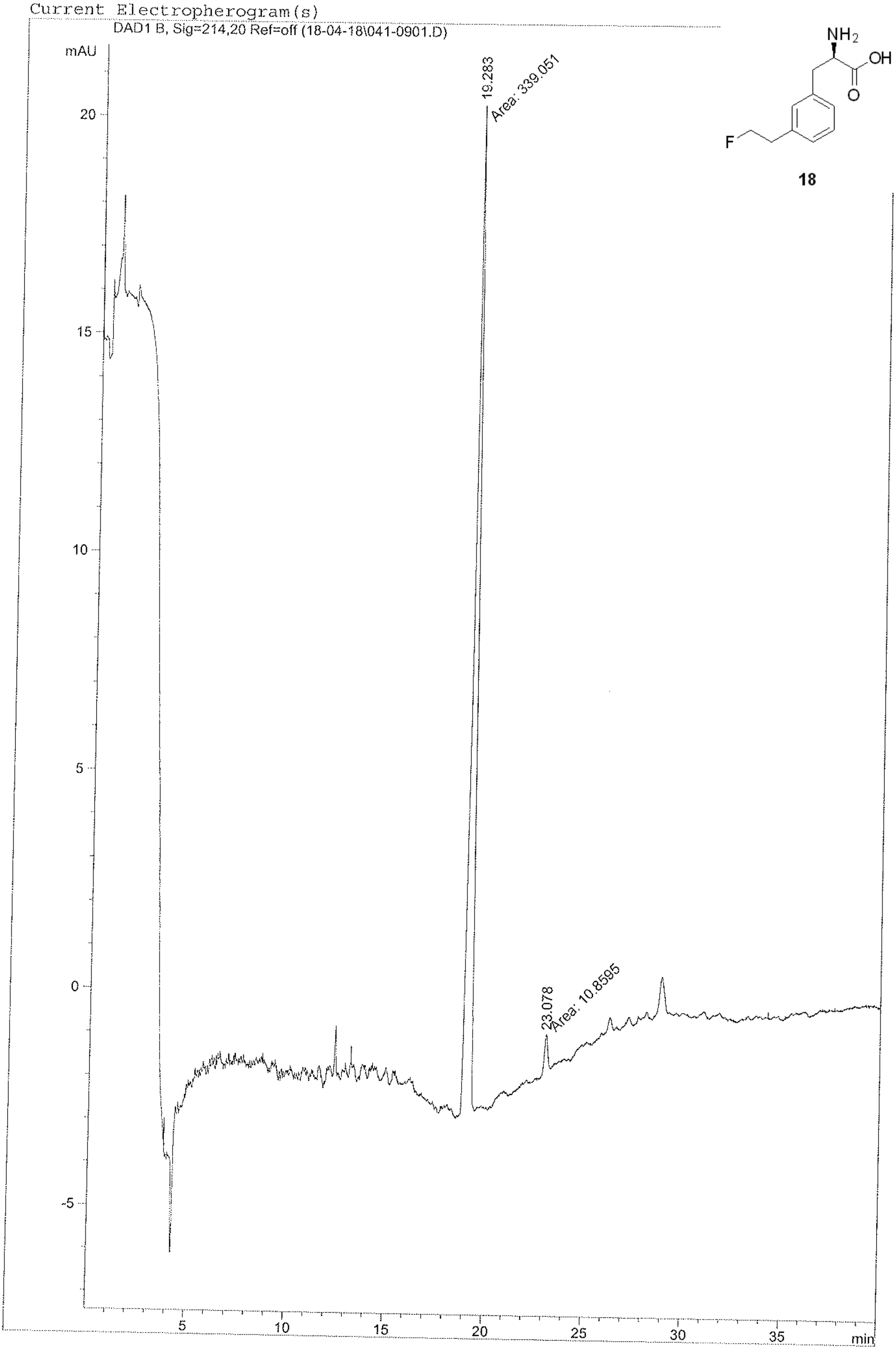
Current Electropherogram(s)
DAD1 B, Sig=214,20 Ref=off (18-04-18\041-0901.D)
mAU
20
15
10
5
0
-5
19.283
Area: 339.051
23.078
Area: 10.8595
5
10
15
20
25
30
35
min
NH2
OH
O
F

18

===
Injection Date : 4/19/18 1:16:34 AM Seq. Line : 9
Sample Name : 30 Location : Vial 41
Acq. Operator : Jan Goeman Inj : 1
Acq. Method : C:\HPCHEM\1\METHODS\CE\01-40N20.M
Last changed : 5/11/11 3:19:59 PM by Jan Goeman
Analysis Method : C:\HPCHEM\1\METHODS\CE\DEF_CE.M
Last changed : 12/15/10 2:53:13 PM by Jan Goeman
Default Method (no analysis)
===
Area Percent Report
===

Sorted By : Signal
Area Calculation Mode : Measured Area
Multiplier : 1.0000
Dilution : 1.0000

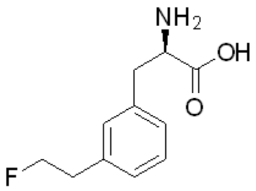

18

Signal 1: DAD1 B, Sig=214,20 Ref=off

Peak #	MigTime [min]	Type	Width [min]	Area [mAU*s]	Height [mAU]	Area %
1	19.283	MM	0.2450	339.05106	23.06212	96.8965
2	23.078	MM	0.1991	10.85953	9.08849e-1	3.1035

Totals : 349.91059 23.97097

Sample: cpd. 18

Results obtained with enhanced integrator! (parabolic interpolation)
===
*** End of Report ***

Chiral purity analysis of compound **32** & **33**

Chromatographic conditions for **32** & **33**:

Astec® Chirobiotic-T column, 125 x 4.6 mm, particle size 5 µm. Column temperature was regulated at ambient temperature. The chromatographic run was performed isocratically with a mixture of EtOH/water (8/2) at a flow rate of 1 mL/min.

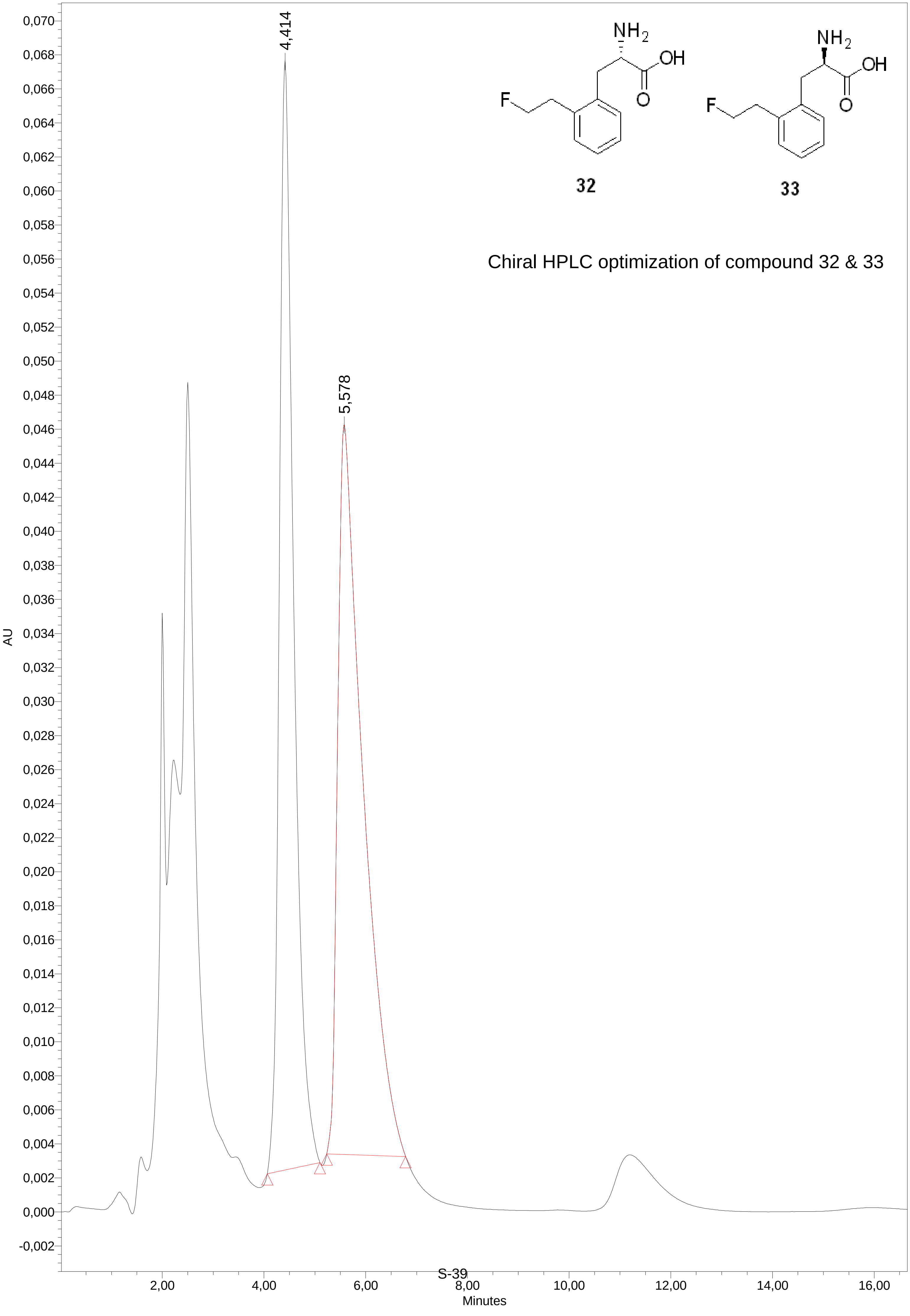
Chiral HPLC optimization of compound 32 & 33
32
33
NH2
OH
O
F
4,414
5,578
AU
Minutes
0,070
0,068
0,066
0,064
0,062
0,060
0,058
0,056
0,054
0,052
0,050
0,048
0,046
0,044
0,042
0,040
0,038
0,036
0,034
0,032
0,030
0,028
0,026
0,024
0,022
0,020
0,018
0,016
0,014
0,012
0,010
0,008
0,006
0,004
0,002
0,000
-0,002
2,00
4,00
6,00
8,00
10,00
12,00
14,00
16,00

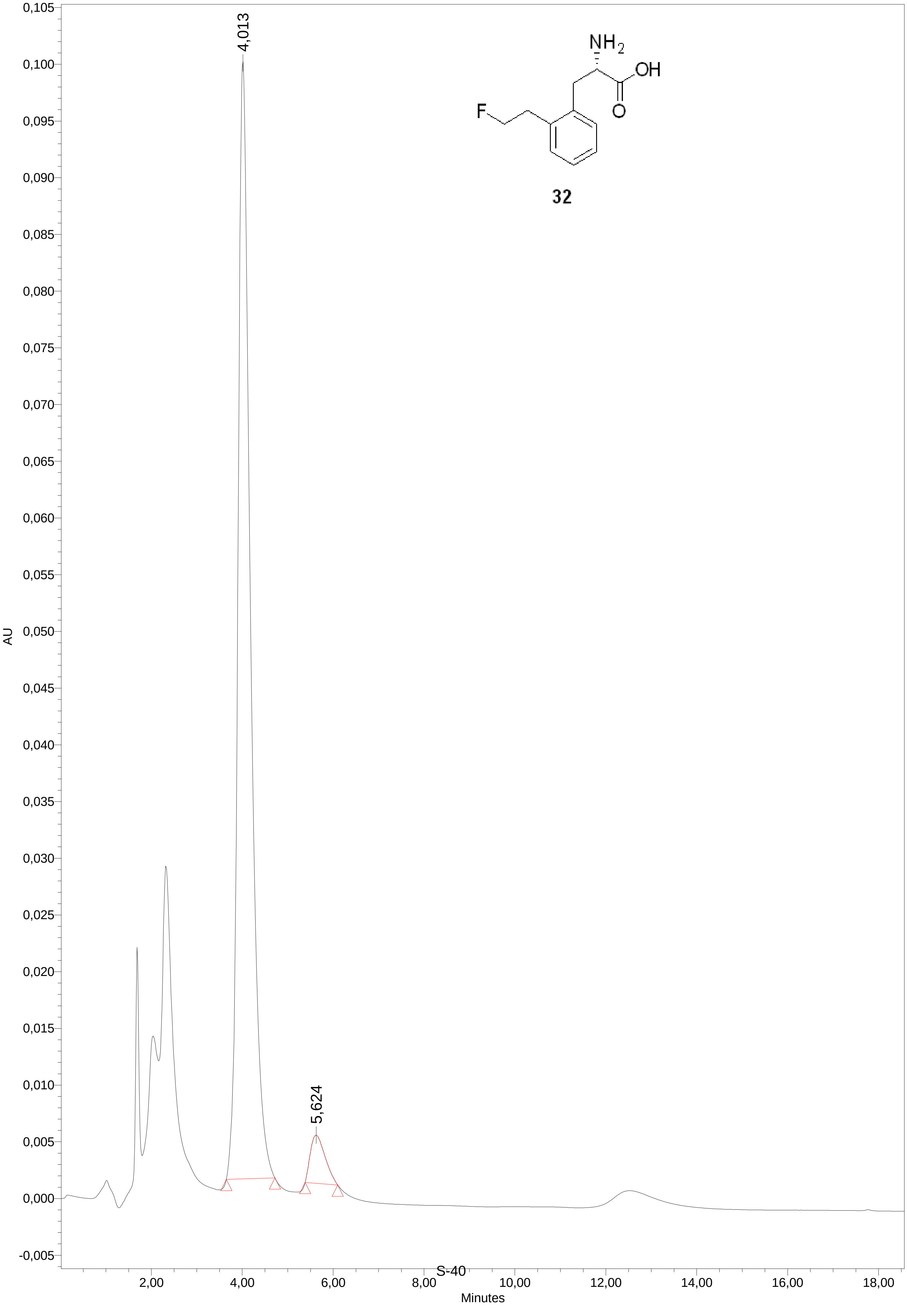
4,013
5,624
NH2
OH
F
O
32
AU
Minutes
0,105
0,100
0,095
0,090
0,085
0,080
0,075
0,070
0,065
0,060
0,055
0,050
0,045
0,040
0,035
0,030
0,025
0,020
0,015
0,010
0,005
0,000
-0,005
2,00
4,00
6,00
8,00
10,00
12,00
14,00
16,00
18,00

	Name	Retention Time (min)	Area (µV*sec)	% Area	Height (µV)	Int Type	Amount	Units	Peak Type	Peak Codes
1		4,013	1896688	95,21	98536	bb			Unknown	
2		5,624	95519	4,79	4250	bb			Unknown	

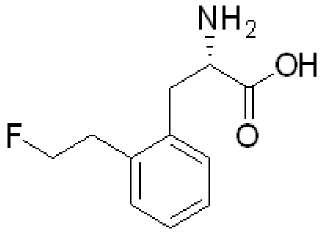

32

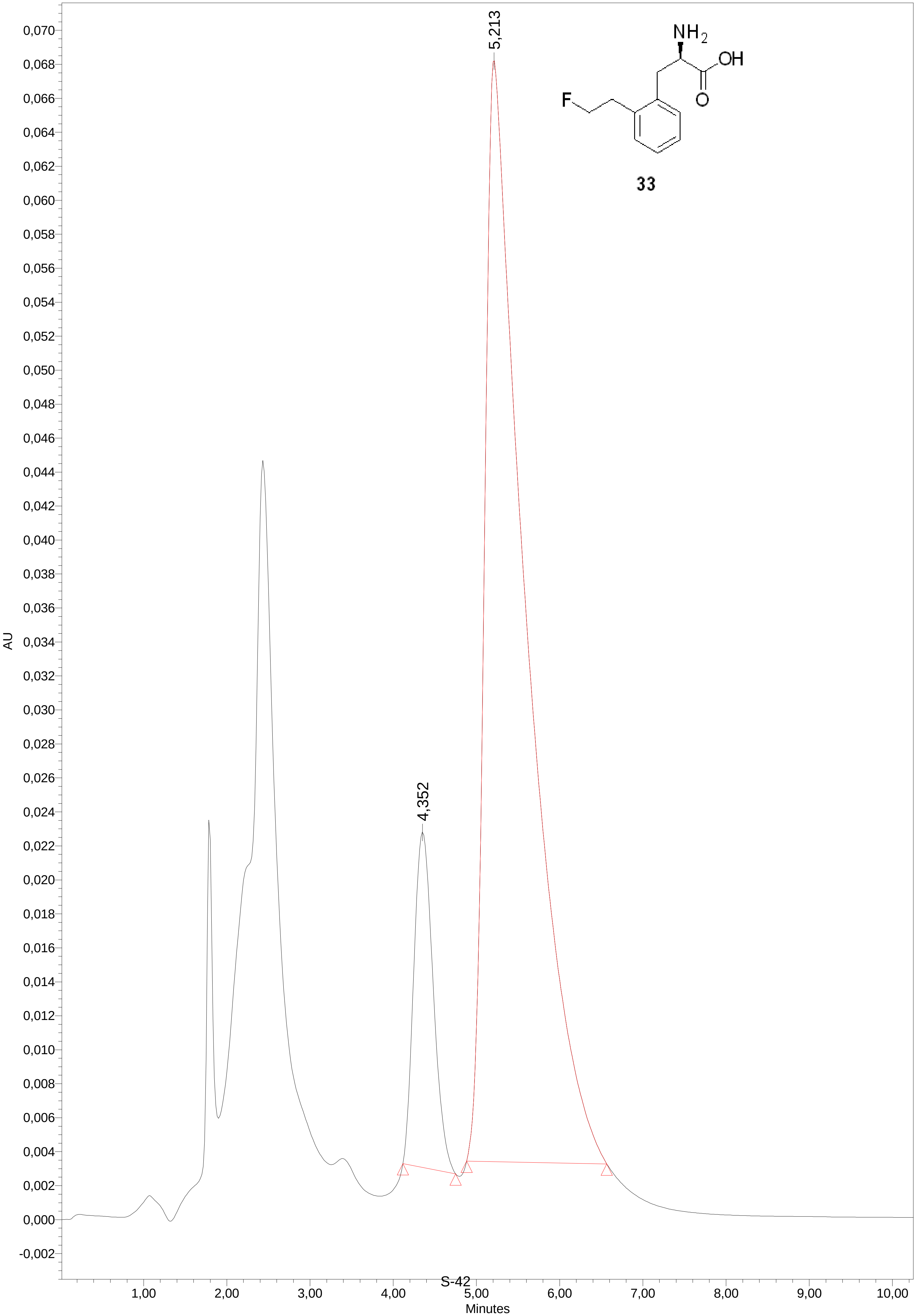

5,213
4,352
NH2
OH
F
O
33
AU
Minutes

	Name	Retention Time (min)	Area (µV*sec)	% Area	Height (µV)	Int Type	Amount	Units	Peak Type	Peak Codes
1		4,352	319629	12,38	19742	bb			Unknown	
2		5,213	2262906	87,62	64814	bb			Unknown	

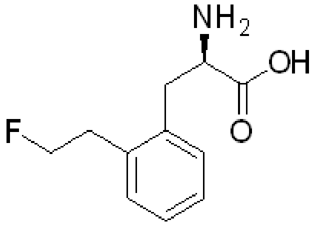

33

Radiosynthesis of [^{18}F]-32 (2-[^{18}F]-FELP): Flow scheme

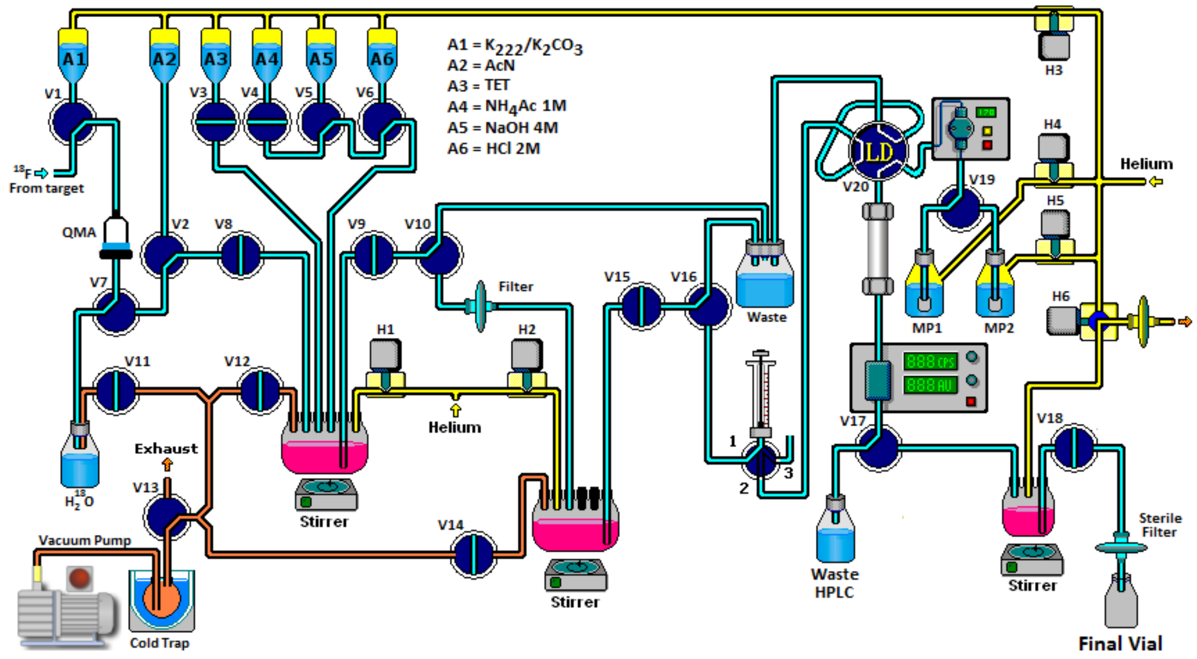

Supplementary Figure 1: Flow scheme of custom Synthra RN plus module (Synthra GmbH, Hamburg,Germany).

Radiogram of [^{18}F]-**32** (2-[^{18}F]-FELP)

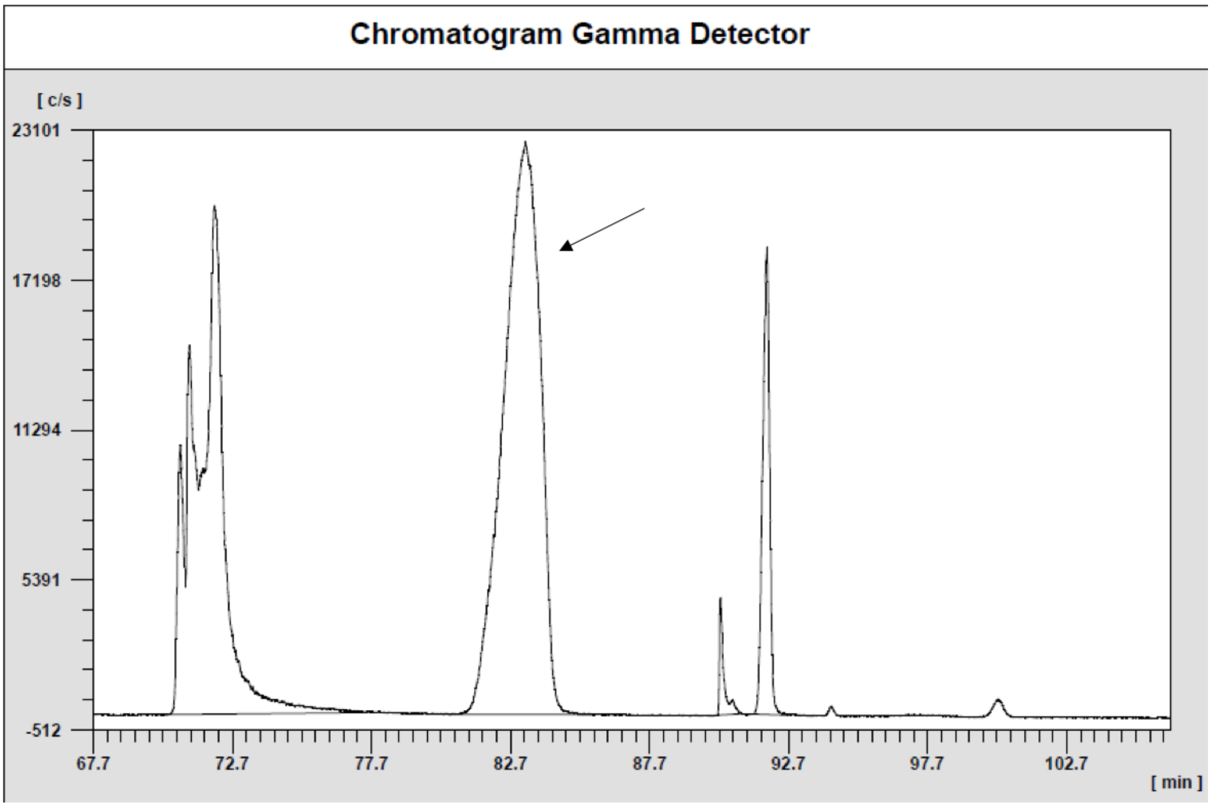

Supplementary Figure 2: Radiogram of radiosynthesized **2-[^{18}F]FELP**. The arrow indicates the **2-[^{18}F]FELP** peak**.**

UV Chromatogram of [^{18}F]-**32** (2-[^{18}F]-FELP)

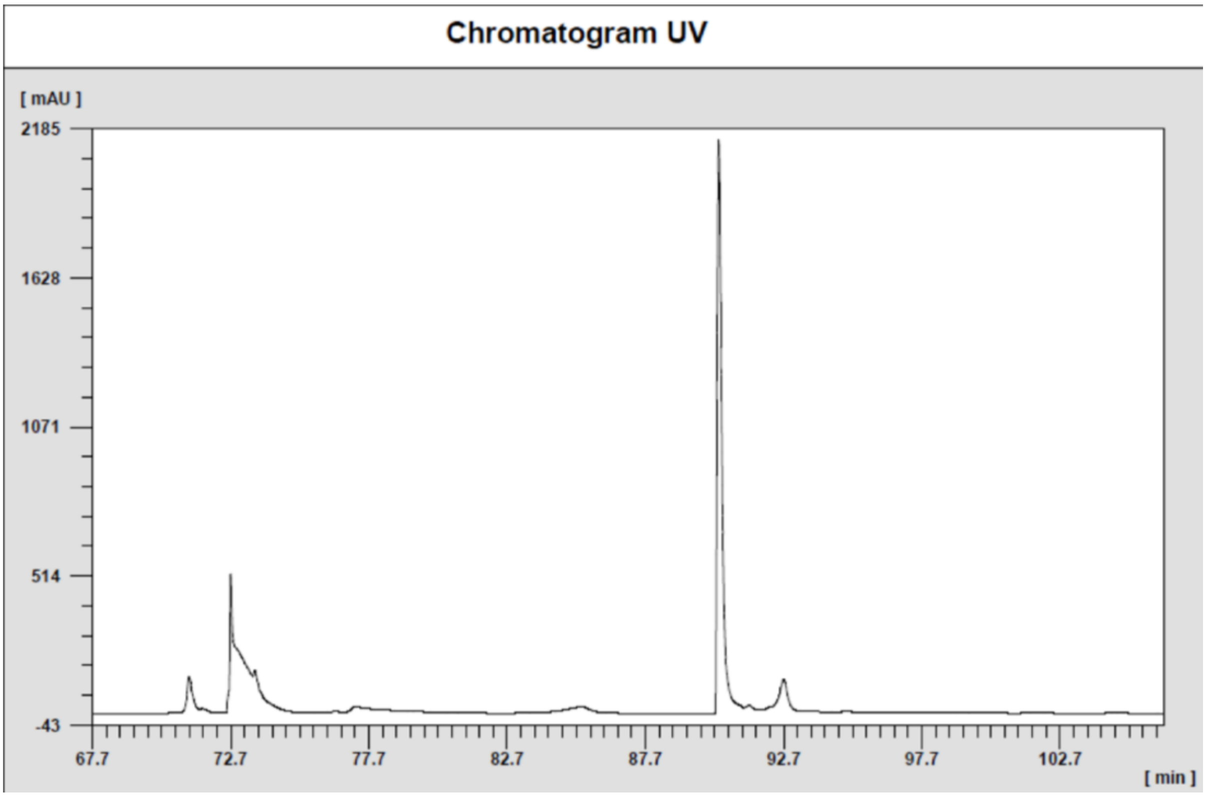

Supplementary Figure 3: UV chromatogram (254 nm) of radiosynthesized **2-[^{18}F]FELP**.

Chiral purity analysis of compound $[^{18}F]$-**32** (2-$[^{18}F]$-FELP)

Chromatographic conditions for 2-$[^{18}F]$-**32**:

Astec® Chirobiotic-T column, 125 x 4.6 mm, particle size 5 µm. Column temperature was regulated at ambient temperature. The chromatographic run was performed isocratically with a mixture of EtOH/water (8/2) at a flow rate of 1 mL/min.

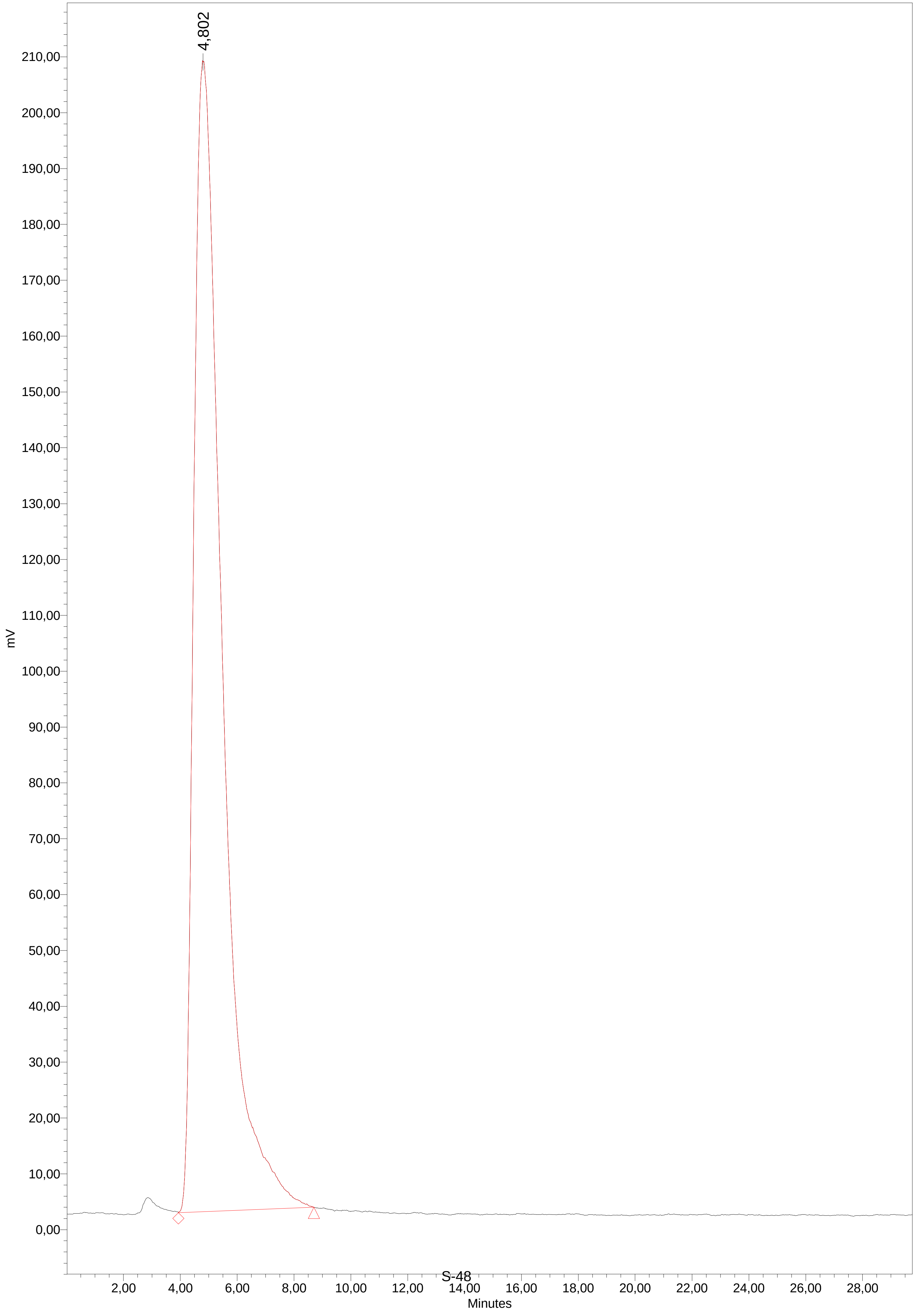
4,802
mV
210,00
200,00
190,00
180,00
170,00
160,00
150,00
140,00
130,00
120,00
110,00
100,00
90,00
80,00
70,00
60,00
50,00
40,00
30,00
20,00
10,00
0,00
S-48
2,00
4,00
6,00
8,00
10,00
12,00
14,00
16,00
18,00
20,00
22,00
24,00
26,00
28,00
Minutes

Flow cytometry

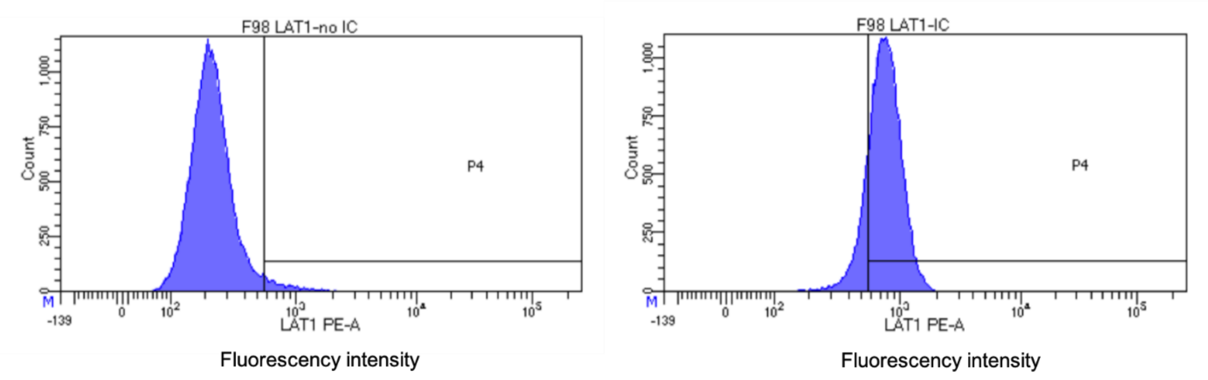

Supplementary Figure 4: Flow cytometry: LAT-1 expression of F98 cells. Left histogram represents surface staining (negative control). Right histogram represents intracellular staining.

Michaelis-Menten plots

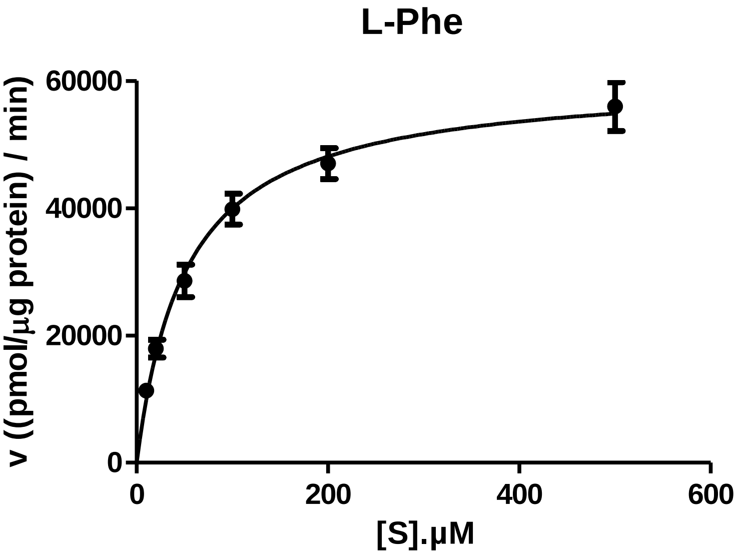

Supplementary Figure 5: Michaelis-Menten plot of [^{3}H]-L-Phe uptake.

Incubation time = 1 min. (*n*=6) K_m = 51.53 ± 7.80 μM

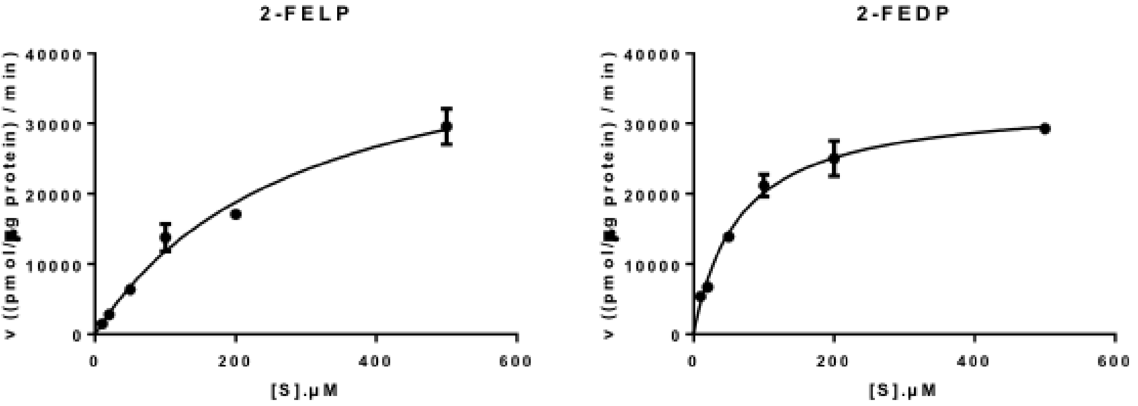

Supplementary Figure 6: Michaelis-Menten plot of [^{3}H]-L-Phe uptake in the presence of **2-FELP** (**32**) (left) or **2-FEDP** (**33**) (right). Incubation time = 1 min. (*n*=6)

K_i **2-FELP** (**32**) = 16.09 ± 3.74 μM. K_i **2-FEDP** (**33**) = 359.4 ± 47.1 μM.

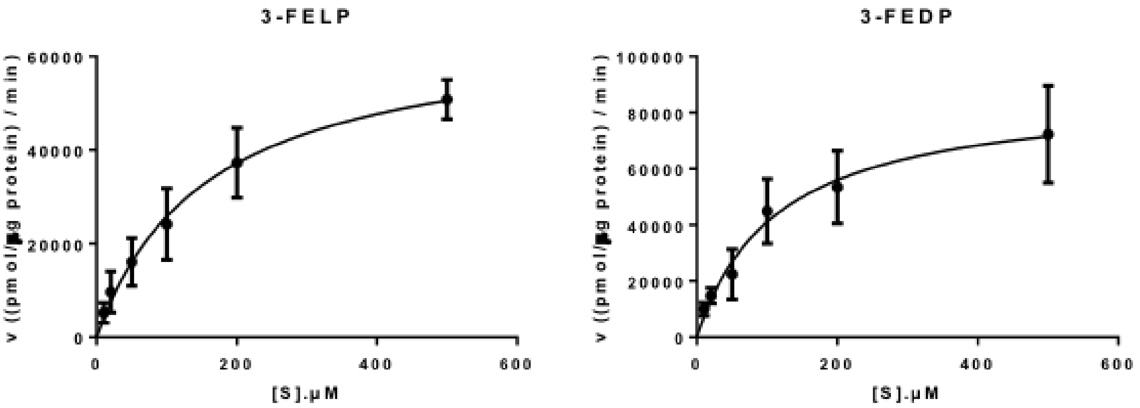

Supplementary Figure 7: Michaelis-Menten plot of [^{3}H]-L-Phe uptake in the presence of **3-FELP** (**17**) (left) or **3-FEDP** (**18**) (right). Incubation time = 1 min. (*n*=6)

K_i **3-FELP** (**17**) = 48.95 $\pm$ 4.92 µM. K_i **3-FEDP** (**18**) = 83.75 $\pm$ 28.1 µM

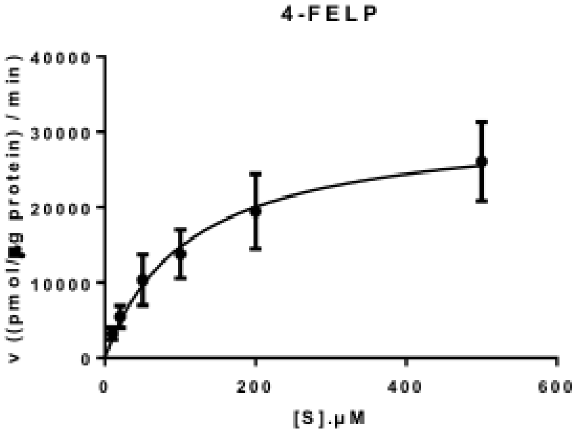

Supplementary Figure 8: Michaelis-Menten plot of [^{3}H]-L-Phe uptake in the presence of **4-FELP** (**15**). Incubation time = 1 min. (*n*=6) K_i **4-FELP** (**15**) = 85.55 $\pm$ 4.40 µM.

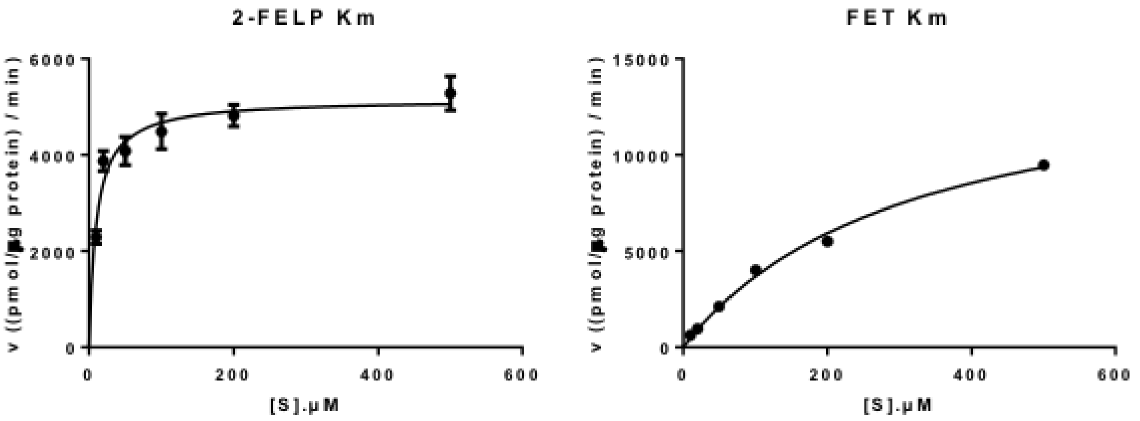

Supplementary Figure 9: Michaelis-Menten plot **2-[^{18}F]FELP (32)** (left) and **[^{18}F]FET** (right) uptake. Incubation time = 1 min. (*n* = 6)

K_m **2-[^{18}F]FELP (32)** = 12.54 ± 3.82 µM. K_m **[^{18}F]FET** = 312.9 ± 25.6 µM

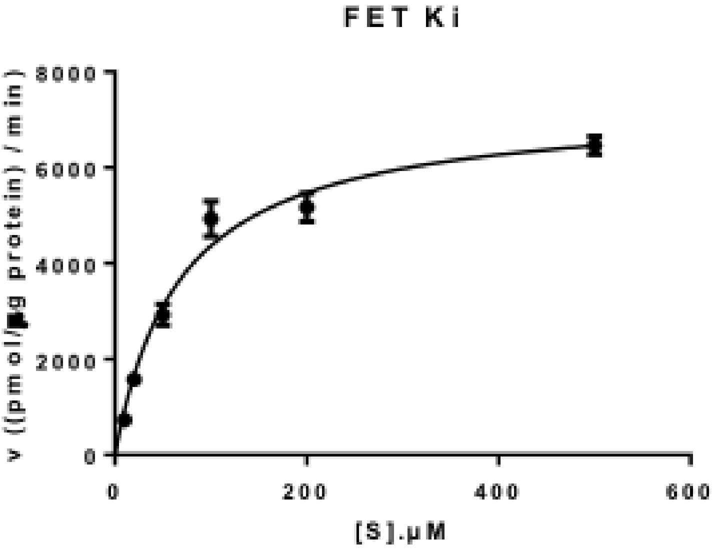

Supplementary Figure 10: Michaelis-Menten plot of [^{3}H]-L-Phe uptake in the presence of **FET**.

Incubation time = 1 min. (*n* = 6) K_i **FET** = 307.2 ± 4.29 µM
